# Comunicaciones LXI Reunión Anual de la SENFC Granada, 29 a 31 de Octubre de 2025

**DOI:** 10.31083/RN50865

**Published:** 2026-04-22

**Caso de Crisis Auditivas Debidas a Epilepsia Focal Refractaria por Displasia Cortical Focal Tipo II: El Valor de la ESI**

Moisés León Ruiz^1^, Álvaro Beltrán Corbellini^2^, Irene Sánchez-Miranda Román^2^, Irene García Morales^2^, Ainhoa Lorenzo Montilla^3^, Antonio Gil-Nagel Rein^2^, Rafael Toledano Delgado^2^

^1^Hospital Universitario Severo Ochoa, 28914 Madrid, España

^2^Hospital Ruber Internacional, 28034 Madrid, España

^3^Hospital Central de la Defensa Gómez Ulla, 28047 Madrid, España

**Introducción y Objetivos**: Las displasias corticales focales (DCF) son la etiología más común de epilepsia focal refractaria (EFR) pediátrica. Reconocer los patrones ictales-interictales con EEEG es clave para delimitar la zona epileptógena (ZE). La electrical source imaging (ESI) localiza de forma más precisa y no invasiva la ZE. La termocoagulación-por-radiofrecuencia guiada por E-EEG (TCG-RF-E-EEG) tiene fines diagnóstico-terapéuticos. Presentamos un caso de EFR estructural, con crisis epilépticas (CEs) auditivas uni/bilaterales, por DCF-II, tratada con TCG-RF-E-EEG. **Material y Métodos**: Paciente de 10 años con EFR con clusters de 1–2 crisis CEs x 3–4 días c/2semanas, con zumbido monoaural izquierdo seguido por acusia completa (a veces binaural) llegando a progresar a CEs generalizadas tónicas. Tratada con LEV 750 mg/12 h y ZNS/12 h VO, con 1 CE/mes. Estudio previo: analítica normal, VEEG (2 focos: no quirúrgica), RM (posible DCF), RM funcional (dominancia-lingüística-hemisférica-izquierda) y exoma-trío (normal). Se realizó evaluación prequirúrgica. **Resultados**: Estudio neuropsicológico (disfunción mnésica-auditivo-verbal), Wada-test+RM-funcional (similar a previo), V-EEGx5días (ZE: región temporal izquierda), ESI (actividad epileptógena): región temporal superior izquierda (TSI), RM3T/PET-cerebral (disgiria+engrosamiento cortical/hipometabolismo temporal izquierdo) y E-EEG (patrón-rítmico-de-descargas-epileptiformes sugerentes de DCF en ZE visualizada por ESI). Se diagnosticó de EFR por DCF-II TSI. Se realizó TCG-RF-E-EEG, manteniendo LEV: 500 mg/12 h, ZNS: 200 mg/12 h y CLB: 5 mg/24 h VO, sin CEs 6 meses, recurriendo con menor frecuencia. Pendiente de Qx vs. TCG-RF-E-EEG. **Discusión/Conclusiones**: Las CEs auditivas suelen iniciarse en la circunvolución TSI, y ser binaurales, por hipersincronía córtico-cuerpocallosocortical. Ante un caso pediátrico con CEs auditivas farmacorrefractarias, debe sospecharse DCF y realizarse evaluación prequirúrgica, incluyendo ESI+E-EEG+PET-cerebral para facilitar el proceso diagnóstico-terapéutico y mejorar el pronóstico.

**Diagnóstico EEG de Muerte Encefálica en Telemedicina: Retos Médico-Legales y Propuesta de Protocolo**

Octavio Jiménez Vega^1^, Daniel Ojeda Boudeling^2^, Giada Buzzacchera^3^, Inmaculada Rodríguez Ulecia^1^, Amanda Labrador Rodríguez^1^, José Juan Rodríguez Betancor^1^, Ayoze Nauzet González Hernández^1^

^1^Hospital Universitario de Gran Canaria Doctor Negrín, 35010 Las Palmas de Gran Canaria, Las Palmas, España

^2^Autónomo

^3^Consejería de Sanidad, Servicio Canario de Salud, 35071 Las Palmas de Gran Canaria, España

**Introducción y Objetivos**: La expansión de la telemedicina en Neurofisiología plantea nuevos retos, especialmente en pruebas que trascienden el ámbito clínico para adentrarse en el contexto legal. En determinadas áreas geográficas, no existe cobertura presencial de Neurofisiólogos Clínicos en todos los territorios, centralizándose la actividad electroencefalográfica (EEG) y realizándose en modalidad telemática. El objetivo de este trabajo es establecer un protocolo seguro para el Neurofisiólogo ante solicitudes de diagnóstico de Muerte Encefálica (ME) en telemedicina, respetando el marco jurídico vigente. **Material y Métodos**: Se realizó un análisis conjunto con un abogado especializado en Derecho Sanitario y un Perito Médico del Servicio Canario de Salud. Se revisaron el Real Decreto 1723/2012, el Código de Deontología Médica y guías técnicas. Se analizaron las solicitudes recibidas y se propuso un protocolo adaptado que garantizara la actuación conforme a los principios legales. **Resultados**: El análisis evidenció que la legislación vigente requiere la certificación por médicos que hayan examinado directamente al paciente. El EEG puede ser realizado y analizado a distancia como apoyo diagnóstico si se cumplen estrictos requisitos clínicos y técnicos, pero no habilita la certificación de ME en modalidad telemática. Se elaboró un protocolo operativo basado en estas premisas. **Discusión/Conclusiones**: La telemedicina facilita el apoyo diagnóstico en territorios sin Neurofisiología presencial, pero su uso inadecuado en la ME puede implicar graves riesgos éticos y legales. Se aconseja que los informes remotos detallen expresamente su carácter de apoyo diagnóstico, sin valor de certificación clínica, reforzando la trazabilidad y seguridad jurídica. Según la legislación vigente, la certificación de Muerte Encefálica no debería realizarse exclusivamente mediante telemedicina. El EEG telemático puede ser un apoyo fiable si se integra en protocolos estrictos. Será necesaria una futura regulación que clarifique su aplicación en procesos médico-legales sensibles.

**Estudio Comparativo Entre dos Tipos de Electrodos Durante el Registro aEEG: Electrodos de gel Líquido y aCUP-E**

Vicenç Pascual Rubio^1^, Vanesa Rius Costa^1^, Enzo Emilio Von Quednow Mannucci^1^, Ángel Rodríguez Ballabriga^1^, Susana Larrosa Capaces^1^, Angès Rigo Vidal^1^, Albert Fabregat Sanjuan^2^

^1^Hospital Universitario Sant Joan de Reus, 43204 Reus, Tarragona, España

^2^Universitat Rovira i Virgili, 43002 Barcelona, España

**Introducción y Objetivos**: La electroencefalografía por integración de amplitud (aEEG) se realiza en las unidades de cuidados intensivos neonatales (UCIN) para detectar encefalopatías o epilepsia. En la práctica clínica, la aEEG, está probablemente infrautilizada por el gran contenido de artefactos de la señal que pueden dificultar la interpretación del trazado, a pie de cuna, por parte del neonatólogo, e incluso conllevar falsos positivos o falsos negativos. Para minimizar los artefactos es necesario utilizar electrodos diseñados específicamente para neonatos como los de gel líquido, ya que los electrodos de aguja subdermal no son recomendados en neonatos. El objetivo del estudio es comparar los electrodos de uso convencional (gel líquido) con un nuevo electrodo diseñado específicamente para utilizar-se en la UCIN, denominado aCUP-E (advanced cup electrode). **Material y Métodos**: Se realiza un estudio prospectivo de 20 neonatos neurológicamente sanos, 10 pretérmino y 10 a término, a los que se les realiza un aEEG en la UCIN, durante un período de tiempo mínimo de 24 h. El montaje utiliza cada tipo de electrodo, aCUP-E y de gel líquido, en un hemisferio determinado del mismo paciente. Los datos del registro se procesan con Matlab para comparar la señal de los diferentes electrodos. Se valora el % de registro artefactado, el número de recambios de electrodos, la estabilidad de los valores de impedancia y la aparición de artefactos que puedan simular epilepsia. **Resultados**: Los electrodos de gel líquido tienen un mayor % de registro artefactado, una menor adherencia y un mayor número de recambios en comparación con aCUP-E. La estabilidad de la impedancia es mayor en los electrodos aCUP-E. Utilizando los electrodos de gel líquido, en varias ocasiones se han detectado patrones aEEG que simulan epilepsia y que podrían confundir al neonatólogo. **Discusión/Conclusiones**: En comparación con los electrodos convencionales, el nuevo electrodo aCUP-E permite un registro aEEG más estable, disminuye la aparición de artefactos y reduce la carga de trabajo en la UCIN.

**El EEG Como Herramienta Fundamental en la Neurotoxicidad Secundaria al Tratamiento con Cefepime**

Pablo Paniagua de Diego^1^, Melany Mesa Pérez^1^, José Alenjandro Díaz-Llanos Díaz^1^, Valeria Pujante Hernández^1^, Waleska Luján^1^

^1^Hospital Universitario de Candelaria, 38110 Tenerife, España

**Introducción y Objetivos**: Cefepime es una cefalosporina utilizada en el tratamiento de infecciones bacterianas graves. Sus efectos neurotóxicos incluyen alteración del estado mental, encefalopatía, afasia, mioclonías, convulsiones y bajo nivel de conciencia, siendo el mayor factor de riesgo la enfermedad renal. El electroencefalograma (EEG) es crucial para detectar la neurotoxicidad, mostrando frecuentemente alteraciones como ondas trifásicas, enlentecimiento generalizado y estatus epiléptico no convulsivo. La identificación temprana mediante EEG permite la intervención rápida, mejorando los Resultados: clínicos. **Material y Métodos**: Caso 1: mujer de 89 años que ingresa para recambio de prótesis de cadera por infección. Tras inicio de cefepime presenta alteración en el lenguaje y mioclonías. Caso 2: varón de 49 años con antecedentes de espina bífida e infecciones urinarias que ingresa por infección de partes blandas, con una enfermedad renal aguda, empezando tratamiento con cefepime. A los 5 días el paciente se muestra afásico y disminución del nivel de conciencia. **Resultados**: Caso 1: Se realiza EEG al 5° día de tratamiento donde se objetivan ondas trifásicas generalizadas de alta prevalencia. Tras la retirada del antibiótico se produce mejoría clínica. Caso 2: Se solicita EEG con hallazgos de un enlentecimiento de la actividad de base y ondas trifásicas generalizadas de alta prevalencia. Tras la retirada del tratamiento mejora progresivamente la sintomatología neurológica. **Discusión/Conclusiones**: La detección temprana de neurotoxicidad por cefepime es clave para solicitar un EEG, que suele mostrar alteraciones como ondas trifásicas o enlentecimiento de la actividad de base, permitiendo una intervención rápida como la suspensión del fármaco y la corrección de factores de riesgo, mejorando el pronóstico del paciente.

**Anomalías Epileptiformes Occipitales en Niños Menores de 6 Años más Allá de la Epilepsia Autolimitada de la Infancia**

Fernando Conde Maciá^1^, Mónica Vicente Rasoamalala^1^, Miquel Raspall Chaure^1^, Julia Sala Coromina^1^, Nuria Raguer Sanz^1^

^1^Hospital Universitario Vall d´Hebron, 08035 Barcelona, España

**Introducción y Objetivos**: La Epilepsia autolimitada de la infancia con crisis autonómicas, (SeLEAS) se caracteriza por crisis focales autonómicas de baja frecuencia y larga duración, de inicio en la infancia (típicamente entre los 3 y 6 años), normalmente activadas durante el sueño, y con una duración media de 3 años. El electroencefalograma (EEG) intercrítico suele mostrar ondas agudas multifocales de alto voltaje y/o complejos punta-onda, frecuentemente en regiones posteriores. **Material y Métodos**: Estudio descriptivo y retrospectivo que pretende describir los hallazgos electroencefalográficos, así como la clínica, en cuatro pacientes en seguimiento por nuestra unidad entre 2014–2025 con patología que se presentó inicialmente con un cuadro electroclínico sugestivo de Panayiotopoulos/SELEAS. **Resultados**: Se presentan 4 pacientes (2 niños y 2 niñas) de edades entre 3 y 5 años en los que inicialmente se detectaron anomalías paroxísticas epileptiformes interictales bioccipitales. Paciente 1: Realizó múltiples cluster de crisis, así como estados epilépticos. Finalmente fue diagnosticado de Sd. MELAS a los 5 años y exitus a los 8 años; Paciente 2: Presentó episodios de visión borrosa/luces de colores y dificultades psicomotrices. Finalmente fue diagnosticado de polimicrogiria perisilviana bilateral; Paciente 3: Desarrolló una encefalopatía epiléptica y del desarrollo no filiada. Se realizó un exoma que detectó una variante patogénica única en CLN8 heredada del padre; Paciente 4: Presentó diversas crisis generalizadas. Se detectó una deleción de novo en los genes 2p24.22p.23.3 que incluye *ASXL2* (Shashi Pena) y *DNMT3A* (Tatton-Brown-Rahman). **Discusión/Conclusiones**: A pesar de que tanto las características iniciales del EEG, como la edad del paciente sugieran una epilepsia autolimitada de la infancia, debemos ser cuidadosos a la hora de clasificarla como tal, ya que en función de la evolución clínica, puede derivar en síndromes de peor pronóstico, como es el caso de nuestros pacientes.

**Random Forest Como Herramienta Para Clasificar Patrones Electroencefalográficos de Epilepsia Generalizada Idiopática**

Juan Manuel Escobar Montalvo^1^, Camila Bautista^2^, Fredy Escobar^3^, Ana María Torres Aranda^4^, Yoel Arroyo^4^, Jorge Mateo Sotos^4^

^1^Servicio de Neurología y Neurofisiología Clínica – Hospital Universitario del Henares, Madrid. Grupo Experto en Análisis Médico (Instituto de Tecnología), Universidad de Castilla-La Mancha (Cuenca, España). Cátedra de Inteligencia Artificial Aplicada a la Salud-Universidad de CastillaLa Mancha, 28822 Cuenca, España

^2^Hospital Universitario La Paz, Madrid,28046 Madrid, España

^3^Servicio de Neurofisiología Clínica – Hospital General Universitario de Ciudad Real, Grupo Experto en Análisis Médico (Instituto de Tecnología), Universidad de Castilla-La Mancha (Cuenca, España), Cátedra de Inteligencia Artificial Aplicada a la Salud-Universidad de Castilla-La Mancha, 28822 Cuenca, España

^4^Grupo Experto en Análisis Médico (Instituto de Tecnología), Universidad de Castilla-La Mancha (Cuenca, España), Cátedra de Inteligencia Artificial Aplicada a la Salud-Universidad de Castilla-La Mancha, Cuenca, España

**Introducción y Objetivos**: La epilepsia generalizada idiopática (EGI) impacta en la salud y la calidad de vida de las personas que la padecen, por lo que su diagnóstico y tratamiento precoz es fundamental. El electroencefalograma (EEG) es esencial en el diagnóstico de EGI y su análisis con modelos de machine learning (ML) podría mejorar el tiempo de diagnóstico y tratamiento. El objetivo fue estudiar la validez del método de random forest (RF) para clasificar patrones EEG de EGI. **Material y Métodos**: Estudio de aprendizaje automático supervizado de clasificación realizado a partir de un dataset de EEGs de EGI y sanos. Tras procesar la señal EEG se construyeron modelos de RF. El rendimiento del modelo de RF y su capacidad para clasificar partrones EEG de EGI se evaluó con la sensibilidad, precisión, F1-score, valor predictivo positivo, área bajo la curva y radar plots. Los Resultados: de RF se compararon con los de otros modelos de ML y la validación externa se realizó con una muestra externa de EEGs. El análisis de datos se realizó con los softwares Matlab y Phyton. **Resultados**: El modelo de RF obtuvo Resultados: óptimos para clasificar patrones EEG de EGI (sensibilidad = 90,5%, precisión = 90,4%, F1score = 90,1% y valor predictivo positivo = 90%), un rendimiento adecuado (índice de Youden = 0,90, AUC = 0,90, índice kappa = 0,80% y coeficiente de correlación de Matheus = 0,80) y mejores Resultados: que otros modelos de machine learning analizados. La validación externa del modelo de RF seleccionado realizada en datos diferentes del dataset de EEGs de entrenamiento mostró Resultados: de precisión, exactitud y valores predictivos de características similares a los obtenidos con el conjunto de datos de entrenamiento. **Discusión/Conclusiones**: En las muestras empleadas de datos EEG de sujetos con EGI y sanos, el algoritmo de random forest presentó una alta validez y precisión, un rendimiento óptimo para clasificar patrones EEG de EGI y métricas de validez externa óptimas y similares a las obtenidas con los datos de entrenamiento.

**Paciente con Síndrome de Lance-Adams Tras COVID-19. A Propósito de un Caso**

Beatriz Rosado Peña^1^, Mariluz Carmona Pérez^1^, Carlos Jarava Luque^1^, Jose Luis Puerto Alonso^1^, Juan-Bosco López Sáez^1^

^1^Hospital Universitario de Puerto Real, 11518 Cádiz, España

**Introducción y Objetivos**: El mioclono posthipóxico crónico, o síndrome de Lance-Adams, fue descrito en 1963 como un cuadro de mioclonías desencadenadas predominantemente por la acción, días o semanas después, de un daño cerebral hipóxico. Se trata de una entidad infrecuente (un 1,5% de casos tras una parada cardiorrespiratoria). El tratamiento habitual son los fármacos antiepilépticos y las mioclonías habitualmente son refractarias a su uso. **Material y Métodos**: Caso clínico. Paciente varón de 61 años, ingresado por neumonía bilateral por COVID-19, con larga estancia en la unidad de cuidados intensivos. Presenta desde la semana anterior al ingreso, tos seca, disnea, malestar general y febrícula, por lo que acude y es diagnosticado con PCR positiva. Dado la mala evolución con mayores requerimientos de oxigenoterapia, se decide IOT con mal manejo de secreciones. Proceso de destete dificultoso con delirio y secreciones muy abundantes. Dentro de este contexto presenta parada cardiorrespiratoria, realizándose RCP avanzada de 10 minutos. A los pocos días, y tras retirarle el rocuronio y el midazolam, presenta mioclonías faciales espontaneas, sospechando inicialmente una encefalopatía postanóxica tras PCR, y cediendo con midazolam. **Resultados**: EEG inicialmente compatible con status parcial, que mejora con midazolam, y levetiracetam. Posteriormente desaparecen las mioclonías en reposo y mejoría del EEG, pero persisten las mioclonías de acción y reflejas, que le generan al paciente una gran incapacidad para actos motores. **Discusión/Conclusiones**: En el caso del mioclono posthipóxico crónico, la presencia de mioclonías tras parada cardiorrespiratoria puede tener diferentes orígenes: cortical, subcortical, reticular, etc. Presentan predominio distal y pueden ser tanto positivas como negativas, y generar caídas. El tratamiento recomendado son los fármacos antiepilépticos, y son de primera línea el valproico, clonacepam o levetiracetam. No suelen tener correlato con el EEG.

**Experiencia con EEG Convencional en Pacientes Ingresados Para Terapia CART en el Hospital Universitario A Coruña (CHUAC)**

Helena Castelo Galván^1^, Catia María Martínez Barjas^1^, Antonio Gómez Rodríguez^1^, Antonio Novoa Vidal^1^, Octavio José Rodríguez Gómez^1^, Víctor Noriega Concepción^1^, Rubén Rubén Fernandez^1^

^1^Complejo Hospitalario Universitario de A Coruña (CHUAC), 15006 A Coruña, España

**Introducción y Objetivos**: El síndrome de neurotoxicidad asociado a células inmunitarias (ICANS) es una posible complicación de la inmunoterapia tipo CART. Junto a la clínica; afasia, alteración de nivel de conciencia, agrafía, etc., el trazado EEG puede alterarse; desde un trazado con predominio de ritmos theta y delta hasta un patrón de status epiléptico no convulsivo (SENC), considerado una urgencia neurológica. La presente revisión describe los hallazgos electroencefalográficos atendidos en el servicio de Neurofisiología Clínica desde la autorización de la terapia CART a finales del año 2022 hasta mayo del 2025 en el CHUAC. **Material y Métodos**: Estudio descriptivo de un total de 21 pacientes ingresados para la infusión de CART que siguiendo el protocolo solicitan la realización de EEG convencional. **Resultados**: Se estudiaron 21 pacientes; uno fue cancelado y otro no se llegó a realizar CART. Un 57,14% de los casos fueron hombres y un 42,86% mujeres. Todos presentaban Linfoma No Hodgkin refractario y el 29% un linfoma de células del manto (LCM). Solo uno tenía EEG previos a la infusión, sin hallazgos relevantes. En cuanto a imágenes, el 38,46% de aquellos que tenían una RM previa presentaban alteraciones frente al 23,07%. En el primer EEG postCART se observó mayoritariamente una encefalopatía moderada con reactividad a estímulo (70%). El 35% presentaban un ICANS 1 en el primer EEG y un 25% un ICANS 3. El 55% alcanzó un ICANS 4. El SENC no se encontró pero sí un patrón Ictal-Interictal Continuum y 2 pacientes presentaron crisis eléctricas. Del total, sobrevivieron 15 y durante el ingreso fallecieron 4 pacientes, uno con crisis epilépticas. Para el servicio de Neurofisiología la terapia CART supuso 74 EEGs adicionales en 30 meses. **Discusión/Conclusiones**: En nuestro centro, el primer EEG es solicitado mayormente con un ICANS 1. El hallazgo EEG predominante es un enlentecimiento difuso del trazado con reactividad a estímulos. En nuestra serie no se encontró ningún SENC. Nuestra experiencia evidencia una baja tasa de crisis, así como de mortalidad.

**Encefalitis por Anticuerpos Anti-Nmda en Paciente de 3 Años de Edad: A Propósito de un Caso**

Guillermo García-Rodríguez Lomas^1^, Raquel López-Carvajal Hijosa^1^, Gabriela Alejandra Naranjo Heredia^1^, Madeleyn Rodríguez Jiménez^1^, María Mercedes Picornell Darder^1^

^1^Hospital Universitario de Móstoles, 28938 Madrid, España

**Introducción y Objetivos**: Introducción: La encefalitis por anticuerpos anti-NMDA es una inflamación del sistema nervioso central caracterizada por la presencia de autoanticuerpos contra subunidades del receptor de NMDA. Produce una amplia variedad de síntomas que abarcan desde alteraciones del comportamiento, alucinaciones visuales, trastornos del sueño, convulsiones o disautonomías. En la literatura no se han descrito casos en pacientes de edad pediátrica. Objetivo: presentación de un caso clínico de encefalitis anti-NMDA en una paciente de 3 años, con el fin de destacar la importancia del diagnóstico precoz y del enfoque clínicovideoEEG poligráfico. **Material y Métodos**: Se trata de una niña de 3 años que debutó con clínica de regresión a nivel motor, regresión del lenguaje, alteraciones del comportamiento y del sueño. Se realizó una revisión de la historia clínica de la paciente atendida en el servicio de neuropediatría del Hospital Universitario de Móstoles, en la que se recogieron datos clínicos, analíticos y de imagen. Las pruebas diagnósticas incluyeron videoEEG de vigilia y sueño, EMG entre otras. **Resultados**: En el video EEG poligráfico realizado previo al tratamiento se objetivaron anomalías focales epileptiformes intercríticas en áreas frontales/fronto-centrales de predominio en hemisferio derecho. Una vez finalizado el tratamiento, se le realizó otro videoEEG poligráfico en el que se observó una disminución de las anomalías focales epileptiformes intercríticas sin registrar descargas críticas. **Discusión/Conclusiones**: Destacar la importancia de la realización de videoEEG poligráfico previo al tratamiento, para apoyar el diagnóstico ante la sospecha clínica de esta patología, y, posterior a ello, para confirmar la mejoría a nivel neurológico.

**Epilepsia Asociada a Procesos Autoinmunes por Anticuerpos Anti-GAD65: A Propósito de un Caso y Revisión de la Literatura**

Estefanía Alonso Sánchez^1^, Adriana Gómez Domínguez^1^, Ana Diez Barrio^2^, Alberto Sáez Marín^1^, Antonio Díaz Negrillo^1^, Beatriz Estrella León^1^, Blanca Patricia Díaz Montoya^1^

^1^Hospital Universitario Infanta Elena, 28342 Madrid, España

^2^Hospital San Pedro de Logroño, 26006 Logroño, La Rioja, España

**Introducción y Objetivos**: La Epilepsia asociada a procesos autoinmunes (EAA) se caracteriza por crisis epilépticas recurrentes en el contexto de disrregulación inmune del sistema nervioso central. Los anticuerpos anti-GAD65 se asocian a epilepsias refractarias con neuroimagen sutil, siendo un desafío diagnóstico y terapéutico. El EEG puede ser útil en la sospecha y monitorización de la actividad epileptiforme. **Material y Métodos**: Se presenta un caso clínico de EAA-GAD65, destacando el papel del EEG en su diagnóstico y seguimiento. **Resultados**: Mujer de 69 años con síndrome poliglandular autoinmune y epilepsia focal de difícil control, con EEGs previos normales. Ante empeoramiento funcional y crisis refractarias, se realizó un videoEEG nocturno que mostró por primera vez puntas en región temporal izquierdo de una escasa persistencia y punta-onda aislada en región fronto-temporal derecha. Estos hallazgos, junto con deterioro cognitivo, ataxia y títulos elevados de anti-GAD65 en suero y LCR, permitieron el diagnóstico de EAA-GAD65. Se inició tratamiento con inmunoglobulinas, con mejoría clínica inicial. Posteriormente, la paciente presentó empeoramiento cognitivo y aumento de crisis, lo que motivó un EEG de control. Este mostró un incremento de la actividad epileptiforme: paroxismos de ondas agudas en regiones fronto-temporales derechas durante el sueño y puntas de bajo voltaje de distribución difusa (predominio hemisférico derecho) con persistencia moderada-alta. Este empeoramiento electroclínico llevó a iniciar rituximab, evidenciando el valor del EEG en la evaluación de la progresión y en la toma de decisiones terapéuticas. **Discusión/Conclusiones**: La EAA-GAD65 es una entidad infrecuente y compleja. En nuestra revisión de la literatura, los hallazgos EEG suelen ser inespecíficos o sutiles, lo que dificulta su reconocimiento. Sin embargo, su análisis integrado con la clínica y los hallazgos inmunológicos puede aportar información clave para orientar el diagnóstico y optimizar el abordaje terapéutico.

**Estudio Observacional Retrospectivo en Epilepsia Refractaria y Trastorno Paroxístico no Epiléptico (TNPE) Concomitante**

María Isabel. Navarrete Páez^1^, A. Nieto Jimenez^1^, R. Chamorro Portilla^1^, M. Cobo Moreno^1^, L. Lorente Remon^1^, C. Cazorla Cabrera^1^, A. Galdón Castillo^1^

^1^Hospital Virgen de las Nieves, 18012 Granada, España

**Introducción y Objetivos**: Analizar características clínico-sociodemográficas en epilepsia refractaria y TNPE concomitantes. **Material y Métodos**: Diseño: estudio observacional/retrospectivo. Muestra: 29 pacientes, de un total de 2888 pacientes ingresados para VEEG.  Variables: edad media, sexo, frecuencia crisis/TPNE, edad inicio epilepsia/TNPE, comorbilidades (física/psíquica), ingresos en UCI, situación laboral, desencadenantes de TNPE, y antecedentes personales de cirugía para la epilepsia. **Resultados**: Edad media: 38,27%. Sexo: Mujeres 65,6%, Hombres 34,4%; Frecuencia crisis epilépticas: diarias 31%, mensuales 17,2%, semanales 20,6%, variable 17,2%. Frecuencia TPNE: Variable 48,8%, diario 44,8%, semanal 3,4%. Edad inicio epilepsia: 1º década vida: 31%, 2º década 13,7%, 3º década 17,2%, 4º década 24,1%, 5º década 6,8%. Aparición TPNE: >5 años 20,6%, 5–15 años 27,5%, >15 años 27,5%, desconocido 3,4%. Comorbilidad psiquiátrica: depresión 44,8%, ansiedad 13,7%, trastorno conversivo 6,8%, TEA 6,8%, trastorno de conducta 3,4%, no comorbilidad 13,7%. Comorbilidad física: no 55,7%, sí 41,3%. Ingreso UCI (estatus): no 93,1%, sí 6,8%. Situación laboral: activo 62%, estudiante 20,6%, pensionista 3,4%, ILT 3,4%, desconocida 6,8%. Desencadenantes TPNE: Desconocido 55,1%, problemas familiares 13,7%, enfermedad propia 10,3%, muerte familiar 3,4%, problemas laborales 3,4%, no 6,8%. Cirugía epilepsia: No 96,5%, Sí 3,4%. **Discusión/Conclusiones**: Los pacientes con diagnóstico de epilepsia que presenta TPNE concomitantes, ocurre con más frecuencia en: mujeres, con una frecuencia de crisis epiléptica diaria, y de TPNE muy variable, edad de inicio de epilepsia más habitual en la 1º década de la vida, con aparición posterior de TPNE >5 años del diagnóstico, mayor comorbilidad de depresión, y sin comorbilidad física, situación laboral activa, la mayoría no precisó ingreso en UCI por estatus, en el conjunto dominante se desconocía desencadenantes de TPNE, ni habían sido sometido a cirugía de epilepsia.

**Ictal-Interictal Continuum: Revisión Bibliográfica a Partir de una Serie de Casos**

Laura Menéndez Rúa^1^, Cielo Mendoza González^1^, Raúl Armas Zurita^1^, Goran Josic^1^, Virginia Russu Russu^2^, Fadi Hallal Peche^1^, Mariano Aguilera Vergara^1^

^1^Hospital Central de La Defensa Gómez Ulla, 28047 Madrid, España

^2^Hospital Puerta de Hierro, 28222 Madrid, España

**Introducción y Objetivos**: El patrón ictal-interictal continuum (IIC) es un concepto electroencefalográfico que describe una actividad “potencialmente ictal” que no cumple criterios de estatus epiléptico no convulsivo (NCSE), situándose en una ¨zona gris¨. En 2021, la Asociación Americana de Neurofisiología Clínica (ACNS) propuso una estandarización de criterios para facilitar su interpretación. El objetivo de este artículo es realizar una revisión bibliográfica del tema a propósito de una serie de casos. **Material y Métodos**: Búsqueda retrospectiva en la base de datos del servicio de neurofisiología del Hospital Gómez Ulla, encontrando 5 pacientes con un patrón compatible con los criterios de IIC (ACNS 2021). **Resultados**: Se analizaron 5 pacientes con patrones de EEG compatibles con IIC. 3 fueron diagnosticados con encefalopatía séptica y 2 con enfermedad de Creutzfeldt-Jakob (ECJ). En todos los casos el patrón IIC fue identificado en el EEG inicial, observándose en todos ellos un patrón de GPDs. Un paciente respondió favorablemente al tratamiento con FAES mostrando mejoría clínica en 24 horas y resolución del patrón EEG, cumpliendo criterios de NCSE. Otro mostró únicamente mejoría del patrón EEG, sin mejoría clínica. **Discusión/Conclusiones**: La etiología del IIC es heterogénea, incluye causas agudas (accidentes cerebrovasculares, encefalitis, sepsis, encefalopatía hipóxico-isquémica, tóxico-metabólicas y traumatismos) y causas crónicas (enfermedades neurodegenerativas, destacando la ECJ). Estos patrones reflejan una disfunción neuronal y su interpretación requiere correlación clínica. El tratamiento con FAEs debe individualizarse, priorizándose en casos con sospecha de actividad ictal, especialmente si se observa fluctuación del patrón EEG, modificadores “plus”, o si hay deterioro del nivel de conciencia. Estos hallazgos subrayan la complejidad diagnóstica del IIC y la necesidad de integrar datos clínicos, electroencefalográficos y terapéuticos para diferenciar entre encefalopatías no epilépticas y NCSE, siendo clave la respuesta al tratamiento.

**Implantación de un Programa de TeleEEG en Territorios sin Neurofisiólogo: Barreras Clínicas, Técnicas y Legales**

Génesis Daniela Arteaga Requena^1^, Jiménez Vega^1^, Ayoze González Hernández^1^, Inmaculada Ulecia Rodríguez^1^, Amanda Labrador Rodríguez^1^, David Cañizo Garcìa^1^

^1^Hospital Universitario de Gran Canaria Doctor Negrín, 35010 Las Palmas de Gran Canaria, España

**Introducción y Objetivos**: La falta de especialistas en Neurofisiología Clínica en zonas periféricas del sistema sanitario dificulta el acceso equitativo a estudios de EEG, especialmente en situaciones urgentes y en población pediátrica. Para paliar esta limitación, se implementó un programa de telemedicina que permite la interpretación remota de EEG, buscando mantener la continuidad asistencial. **Material y Métodos**: Durante los primeros seis meses del programa en una gerencia sin neurofisiólogo presencial, los EEG fueron registrados localmente por técnicos y enviados a un centro de referencia para su análisis diferido. Se evaluaron incidencias técnicas y de conectividad, calidad de los registros, tiempos de respuesta diagnóstica, satisfacción del equipo clínico e implicaciones legales. **Resultados**: Se detectaron diversas dificultades, destacando artefactos por colocación inadecuada de electrodos o interferencias no corregidas, lo cual genero numerosas repeticiones de estudios. Se observó falta de comunicación efectiva entre técnicos y el especialista remoto, así como ausencia de protocolos para priorizar EEG urgentes. En el ámbito legal, se plantearon dudas sobre la validez de los informes en situaciones críticas, como el diagnóstico de Estatus Epiléptico o decisiones de limitación terapéutica. **Discusión/Conclusiones**: La teleinterpretación de EEG es una herramienta útil para reducir desigualdades territoriales, pero conlleva riesgos si no se acompaña de formación adecuada, estandarización de procedimientos y respaldo legal. La calidad diagnóstica requiere de un enfoque multidisciplinar y mejora continua. En conclusión, el teleEEG es una opción viable de forma transitoria en contextos donde no hay especialistas disponibles, siempre que se aseguren estándares de calidad, trazabilidad y responsabilidad médica, actuando como un complemento al sistema de salud y no como un reemplazo definitivo.

**Status Epiléptico Superrefractario**

Ramón Josep Gorgues Torres^1^, Shijia Li Chen^1^, Jesús David Jaramillo Álvarez^1^, Ernesto Sánchez Garrigós^1^, Jorge de Francisco Moure^1^, Berenice Abreu Rodríguez^1^

^1^Hospital Universitario Miguel Servet de Zaragoza, 50009 Zaragoza, España

**Introducción y Objetivos**: El estatus epiléptico superrefractario (SESR) es una forma grave de crisis epiléptica que persiste o recurre más allá de 24 horas tras el inicio de anestesia general, a pesar de tratamiento adecuado, representando una emergencia neurológica con alta morbimortalidad. Objetivo: Describir un caso clínico de SESR de origen estructural. Revisar los criterios diagnósticos clínicos y electroencefalográficos del estatus epiléptico. Exponer el abordaje terapéutico, complicaciones y evolución del paciente. Comentar las bases fisiopatológicas, etiologías y pronóstico del SESR. **Material y Métodos**: Varón de 71 años sin dependencia funcional previa, encontrado con hemiparesia izquierda. TC cerebral mostró hematoma lobar temporo-parietal derecho. Se realizaron EEG seriados, tratamiento antiepiléptico escalonado y coma farmacológico. Finalmente, se evacuó quirúrgicamente el hematoma. **Resultados**: El paciente desarrolló crisis focales con actividad epileptiforme en hemisferio derecho, refractarias a tratamiento médico y sedación. Fue trasladado a centro terciario donde se realizó cirugía. EEG posteriores mostraron mejoría parcial, aunque persistía encefalopatía moderada con déficits neurológicos residuales. **Discusión/Conclusiones**: Este caso representa un estatus epiléptico superrefractario secundario a hematoma lobar, en el que fue necesario un enfoque terapéutico agresivo incluyendo tratamiento antiepiléptico escalonado, sedación farmacológico y cirugía. El SESR se caracteriza por mecanismos fisiopatológicos propios que explican su difícil control y su impacto neurológico. Las causas estructurales, como los eventos cerebrovasculares, representan una parte relevante de las etiologías, y su pronóstico depende de factores como la edad, la duración del SE y el daño cerebral subyacente. Este caso ilustra la importancia de una monitorización EEG continua, el trabajo multidisciplinar y el manejo prolongado en UCI como pilares fundamentales para el control del SESR y la reducción de secuelas.

**Neurotoxicidad por Cefepime: Una Alerta Clínica Necesaria**

Claudia Perdomo Rubio^1^, Iratxe Toña Zuazua^1^, María Lourdes Guerra Martín^1^, Jose Luis Macia Cerda^1^

^1^Hospital Universitario Araba, 01009 Vitoria, España

**Introducción y Objetivos**: El aumento de infecciones nosocomiales graves ha impulsado el uso creciente de antibióticos de amplio espectro como el cefepime. Este fármaco, aunque eficaz, conlleva un riesgo relevante de neurotoxicidad, especialmente en pacientes con disfunción renal. Las manifestaciones incluyen alteración del nivel de conciencia, mioclonías, confusión, bradipsiquia, inatención o afasia. La detección precoz es clave, ya que suele ser reversible. El electroencefalograma (EEG) en este contexto, es muy sensible para identificar patrones compatibles con neurotoxicidad como descargas periódicas generalizadas (GPDs) de aspecto trifásico, facilitando decisiones terapéuticas eficaces y rápidas. Describir los hallazgos electroencefalográficos y la cronología de aparición de síntomas neurológicos en pacientes tratados con cefepime, resaltando la utilidad diagnóstica del EEG. **Material y Métodos**: Se estudiaron 6 pacientes (67% mujeres, edad media 78,7 años) ingresados en el Hospital Universitario de Araba entre 2020 y 2025, tratados con cefepime y con sospecha de neurotoxicidad, a los que se solicitó EEG. Se analizó el intervalo entre el inicio del antibiótico, la aparición de síntomas y realización del EEG, junto con comorbilidades, función renal y evolución clínica. **Resultados**: Todos los pacientes desarrollaron síntomas neurológicos. El tiempo medio transcurrido entre el inicio del antibiótico y la clínica fue de 3 días, y entre la clínica y EEG, de 5 días. En cuatro pacientes se hallaron GPDs: tres con morfología trifásica y uno con ondas bifásicas. Un paciente presentó actividad delta rítmica generalizada (GRDA), y otro mostró enlentecimiento difuso sugestivo de encefalopatía tóxica. Cuatro pacientes mejoraron tras suspender cefepime. **Discusión/Conclusiones**: Los datos coinciden con la literatura global. El EEG es una herramienta clave para su detección temprana, especialmente en casos de estatus epiléptico no convulsivo con clínica sutil. Su solicitud precoz, integrada al contexto clínico, puede ser decisiva para mejorar el pronóstico.

**Más Allá del Despiste: La Sospecha del Estatus de Ausencia Infantil**

Justo Marín Noguera^1^, Karina Henríquez Díaz^1^, Víctor Hugo Rubio Suárez^1^, Pilar Rosario Martínez Martínez^1^, Julián Vázquez Lorenzo^1^

^1^Hospital Clínico Universitario Virgen de la Arrixaca, 30120 Murcia, España

**Introducción y Objetivos**: El estatus epiléptico de ausencias (EEA) es una crisis generalizada prolongada, caracterizada por alteración del nivel de conciencia, con o sin automatismos, fenómenos autonómicos o alteración del lenguaje. Se clasifica en EEA típico, vinculado a epilepsias genéticas, y EEA atípico, a encefalopatías del desarrollo (Lennox-Gastaut, Dravet). Puede presentarse además como debut epiléptico (EEA “de novo”). Su expresión clínica puede ser sutil, dificultando el diagnóstico, especialmente en niños. El electroencefalograma (EEG) es clave para su identificación. **Material y Métodos**: Paciente de 9 años, sano, sin antecedentes personales ni familiares de epilepsia. Derivado por episodios mensuales de ensimismamiento de hasta 12 h, descritos como inatención, lentitud en respuestas y párpados entornados, conservando funciones básicas. En la escuela mostraba desorganización y déficit de memoria reciente. Se realizó vídeo-EEG prolongado. **Resultados**: Se registraron descargas generalizadas continuas de complejos punta-onda y polipunta-onda (2,5–3,5 Hz, hasta 100 µV) durante >90% del trazado. El paciente mostraba párpados entornados, lenguaje enlentecido, obedecía a órdenes, era capaz de contar y multiplicar. Se administró midazolam intranasal (10 mg), con resolución clínica y electroencefalográfica. Se inició valproato (carga 800 mg, mantenimiento 200 mg/6 h). El EEG de control a las 2 semanas evidenció escasa actividad epileptiforme en áreas fronto-temporales y una crisis de ausencia típica. Se añadió etosuximida, con evolución favorable y mejoría académica. **Discusión/Conclusiones**: El EEA “de novo” resulta particularmente desafiante de identificar en población pediátrica debido a la sutileza de sus síntomas que pueden confundirse con trastornos de atención, alteraciones conductuales o simplemente con cambios emocionales. Su diagnóstico precoz mediante EEG permite instaurar un tratamiento eficaz. El pronóstico suele ser favorable con adecuado manejo farmacológico.

**Hallazgos Video-EEG en Paciente con Leucoencefalopatía por VIH a Propósito de un Caso**

Gabriela Alejandra Naranjo Heredia^1^, Guillermo García – Rodríguez Lomas^1^, Raquel López-Carvajal Hijosa^1^, Madeleyn Rodríguez Jiménez^1^, María Mercedes Picornell Darder^1^

^1^Hospital Universitario de Móstoles, 28938 Madrid, España

**Introducción y Objetivos**: Introducción: La leucoencefalopatía multifocal progresiva (LMP) es una enfermedad desmielinizante grave del sistema nervioso central causada por la reactivación del virus JC (John Cunningham), en pacientes inmunocomprometidos, como por VIH, se presenta en estadios avanzados de la infección, cuando los niveles de linfocitos CD4 están significativamente disminuidos. La LMP se caracteriza por síntomas neurológicos focales de progresión subaguda, como, hemiparesia, alteraciones visuales, afasia o ataxia. Las convulsiones pueden presentarse hasta en un 18–44% de los casos, especialmente cuando hay afectación cortical y subcortical cercana. Generalmente son focales aunque pueden generalizarse y el riesgo aumenta en pacientes con mayor carga viral del virus JC o con rápida progresión de la enfermedad. Objetivos: Describir los hallazgos registrados (estatus focal y anomalías epileptiformes) en el estudio video-EEG poligráfico en un paciente con LMP. **Material y Métodos**: Varón de 56 años con antecedente de VIH con buen control inmunovirológico, leucoencefalopatía con secuelas de epilepsia focal, disartria, deterioro cognitivo y virus de la hepatitis C, que es traído por el SUMMA por presentar numerosas crisis epilépticas tónico clónicas, bajo nivel de conciencia y además mioclonías en miembro superior derecho, por lo que está en tratamiento con varios fármacos antiepilépticos sin clara mejoría del cuadro. **Resultados**: El elemento que caracteriza el estudio video EEG poligráfico es el registro de crisis focales del lóbulo Frontal izquierdo, que pone de manifiesto un estado de mal epiléptico con crisis focales. La electrogénesis cerebral de vigilia y sueño NREM muestra la existencia de algunas anomalías focales epileptiformes intercríticas (paroxismos de punta-onda) en áreas Frontales/Fronto-Centrales del hemisferio izquierdo. **Discusión/Conclusiones**: La importancia de realizar estudios video-EEG poligráficos de vigilia y sueño para el correcto diagnóstico y seguimiento de estos pacientes.

**Encefalitis antiNMDAR: Evolución del Patrón EEG en Función del Tiempo, Clínica y Tratamiento. A Propósito de un Caso**

Gloria Moreno García^1^, Paloma Balugo Bengoechea^1^, Lorena Iglesias Alonso^1^, Adela Fraile Pereda^1^, Guillermo Ceide García^1^, Gianna Doménika Bozano Subía^1^, Andrea Mier San Juan^1^

^1^Hospital Clínico San Carlos, 28040 Madrid, España

**Introducción y Objetivos**: La encefalitis por anticuerpos antiNMDAR es una de las principales causas de encefalitis autoinmune e indirectamente de NORSE (estado epiléptico refractario de nueva aparición). Clínicamente se caracteriza por alteración del comportamiento, trastornos del movimiento, crisis, disminución del nivel de conciencia y disfunción autonómica. El inicio de tratamiento inmunosupresor e inmunomodulador habitualmente se administra antes de tener la confirmación inmunológica en LCR. El patrón EEG es variable en la literatura, siendo el patrón “extreme delta brush” (EDB) el más específico aunque poco frecuente. Objetivos: describir retrospectivamente la evolución de patrones EEG en una paciente con diagnóstico confirmado de encefalitis antiNMDAR y su papel en el manejo terapéutico y pronóstico. **Material y Métodos**: Se describe el caso de una paciente de 17 años sin antecedentes de interés que presenta un cuadro subagudo de alteración del comportamiento y síntomas de disautonomía con la necesidad de ingreso en UCI. Se realiza EEG en las primeras horas, así como monitorización vídeo-EEG prolongada y EEGs seriados. Con la sospecha clínica y registro EEG se inicia de forma precoz tratamiento corticoideo y FAEs (fármacos antiepilépticos) por sospecha de encefalitis. **Resultados**: En los diferentes estadíos evolutivos de la enfermedad se observa variabilidad en el registro EEG (incluido EDB). Además, se objetivan crisis eléctricas con y sin correlato clínico motor y disautonómico, así como episodios paroxísticos no epilépticos, que guiaron el ajuste farmacológico. **Discusión/Conclusiones**: El registro EEG es una prueba crucial para el manejo y pronóstico de encefalitis antiNMDAR, así como para la identificación de la naturaleza de los eventos paroxísticos epilépticos y no epilépticos, pudiendo presentar una rápida evolución hacia distintos patrones a pesar de un tratamiento temprano. Se deben tener en cuenta los fármacos administrados, como benzodiacepinas, para evitar sobreinterpretar patrones patológicos que pudieran simular un EDB.

**Epilepsia Piridoxina Dependiente: A Propósito de un Caso**

Shijia Li Chen^1^, Ramón Josep Gorgues Torres^1^, Jesús David Jaramillo Álvarez^1^, Isabel Dolz Zaera^1^, Jorge de Francisco Moure^1^

^1^Hospital Universitario Miguel Servet, 50009 Zaragoza, España

**Introducción y Objetivos**: La epilepsia dependiente de piridoxina (PDE) es un trastorno metabólico raro, de herencia autosómica recesiva, causado por deficiencia de la enzima ALDH7A1. Suele manifestarse en las primeras horas de vida con crisis epilépticas refractarias a fármacos antiepilépticos (FAEs) convencionales. El diagnóstico y tratamiento precoz con piridoxina son fundamentales para evitar daños neurológicos irreversibles. **Material y Métodos**: Se presenta el caso de una recién nacida a término que, a las 4 horas de vida, desarrolló rigidez, movimientos de flexo-extensión de extremidades, distrés respiratorio y acidosis metabólica. Ingresó en UCI neonatal con soporte vital y tratamiento con FAEs (fenobarbital, levetiracetam, fenitoína), así como antibióticos y antivirales ante sospecha de encefalopatía hipóxicoisquémica o infecciosa. **Resultados**: Los EEGs iniciales mostraron un patrón de brote-supresión y actividad de punta/onda aguda a alta frecuencia de breve duración, sin mejoría con FAEs. Las pruebas de imagen mostraron un pequeño hematoma subdural parietooccipital derecho, sin signos de daño hipóxico-isquémico. El LCR fue normal, por lo que se suspendieron antibióticos y antivirales. Ante la sospecha de encefalopatía metabólica, se inició un cóctel multivitamínico incluyendo piridoxina y se retiraron los FAEs, observándose una mejoría clínica y electroencefalográfica. Los estudios metabólicos mostraron niveles elevados de ácido pipecólico en sangre. La paciente fue dada de alta con triple terapia (piridoxina, arginina y dieta baja en lisina) y levetiracetam. Meses más tarde, el diagnóstico de PDE se confirmó por estudio genético, identificándose mutaciones en ALDH7A1. **Discusión/Conclusiones**: El EEG constituye una herramienta de gran valor para orientar el diagnóstico y la toma de decisiones clínicas, así como la evaluación de la respuesta farmacológica. En neonatos con crisis epilépticas de inicio precoz y trazado EEG patológico sin etiología clara, debe contemplarse PDE.

**Crisis Epilépticas Neonatales Refractarias: Papel del EEG en el Diagnóstico y el Manejo Terapéutico**

Patricia Orive Rodríguez^1^, Alicia Silva Cátedra^1^, Esteban Nieto Jiménez^1^, Gloria Moreno Madueño^1^, Rocío Vázquez Rodríguez^1^

^1^Hospital Universitario Virgen del Rocío, 41013 Sevilla, España

**Introducción y Objetivos**: Las crisis epilépticas refractarias en neonatos constituyen un reto diagnóstico y terapéutico, especialmente cuando se asocian a malformaciones cerebrales como la hemimegalencefalia. Presentamos un caso clínico de epilepsia farmacorresistente en neonato de tres días de vida con malformación del desarrollo cerebral, en el que el EEG ejerció un papel crucial en el diagnóstico, seguimiento y toma de decisiones. **Material y Métodos**: Se evaluó a un neonato de tres días de vida con crisis convulsivas refractarias a múltiples anticonvulsivantes y RM cerebral que mostró hemimegalencefalia y displasia cortical derecha. Se realizaron estudios seriados de vídeo-EEG en vigilia y sueño para caracterizar la actividad interictal e ictal y correlacionarla con manifestaciones clínicas. Se decidió la intervención quirúrgica. **Resultados**: El EEG inicial evidenció actividad basal bilateral asimétrica, con una disfunción cerebral focal y grafoelementos agudos en el hemisferio derecho. Los estudios videoEEG sucesivos mostraron actividad de fondo lentificada en el hemisferio derecho con anomalías epileptiformes intercríticas frecuentes que se propagaban al hemisferio contralateral. Se registraron crisis electroclínicas caracterizadas por una actividad theta rítmica ictal seguida de descargas rítmicas de gran amplitud, concordantes con estatus epiléptico focal hemisférico derecho. Se realizó cirugía consistente en hemisferotomía funcional derecha con desconexión completa tras la cual el EEG mostró persistencia de descargas interictales y crisis eléctricas focales en hemisferio derecho. **Discusión/Conclusiones**: El EEG resultó fundamental para identificar y monitorizar la actividad epileptiforme en este caso, permitiendo optimizar el tratamiento y apoyar la indicación quirúrgica. La hemisferotomía logró reducir la frecuencia de crisis, aunque persistió la actividad ictal; evidenciando la complejidad del manejo y la necesidad de vigilancia electroencefalográfica continua para el ajuste terapéutico y la mejora del pronóstico funcional.

**Encefalopatía Epiléptica y del Desarrollo (EED) por Mutación en *KCNQ2*. A Propósito de 4 Casos**

Julián Vázquez Lorenzo^1^, Sofía Ortigosa Gómez^1^, Carmen María Garnés Sánchez^1^, Pilar Rosario Martínez Martínez^1^, Victor Hugo Rubio Suárez^1^, Reetika Baharani Baharani^2^, Justo Marín Noguera^1^

^1^Hospital Universitario Virgen de La Arrixaca, 30120 Murcia, España

^2^Hospital Morales Meseguer, 30008 Murcia, España

**Introducción y Objetivos**: Las mutaciones en el gen *KCNQ2* son la segunda causa genética más frecuente de epilepsia infantil y la primera en neonatos. **Material y Métodos**: Descripción de cuatro pacientes con mutación en *KCNQ2*. **Resultados**: Tres pacientes mujeres y un varón, con inicio de crisis precoz (entre 1 y 18 días). Semiología crisis: Tónicas asimétricas alternantes de alta frecuencia, y uno de los pacientes desarrolló espasmos epilépticos. EEG: Actividad de fondo anormal en todos los pacientes (exceso de discontinuidad, lentificación y/o pobre estructuración). Afectación multifocal intercrítica e hipsarritmia en dos de los pacientes. Todos los pacientes evolucionaron a cuadro de discapacidad grave/moderada. Tres precisaron combinación de varios FAC, siendo la introducción de FAC bloqueantes del sodio determinante. Sólo un paciente está libre de crisis. **Discusión/Conclusiones**: El EEG de los pacientes *KCNQ2* se caracterizan por un patrón discontinuo (a veces tipo brote-supresión) o lentificado sobre el que se imponen anomalías epileptiformes multifocales en un contexto de crisis neonatales precoces que suelen ser tónicas asimétricas originadas de forma alternante o síncrona en ambos hemisferios. La literatura describe pobre control de crisis, con una respuesta parcial o total en algunos pacientes tras el inicio de FAC bloqueantes del sodio y ACTH; todos los pacientes presentan alteraciones graves del desarrollo psicomotor, al igual que en nuestra serie. Las características EEG de nuestros pacientes concuerdan con lo publicado, remarcando la presencia de patrón de hipsarritmia y espasmos epilépticos en dos pacientes. La EED por mutaciones *KCNQ2* presenta unas características fenotípicas y electroencefalográficas que, si bien no específicas, permiten la sospecha clínica precoz. Es importante de identificar, pues el tratamiento precoz con FAC bloqueantes del sodio podría favorecer una mejor evolución. Nuestra serie tiene características similares a las publicadas, subrayando la presencia de hipsarritmia en dos de los pacientes.

**OIRDA Como Factor de Buen Pronóstico**

Pedro García Frutos^1^, Luis Santoveña González^1^, Alexandra Desiree Dávila Andrade^1^, Alejandro José Soriano García^1^, Eva Rey Pérez^1^, Maruvic Boruska Ramírez Arvelo^1^, Jesús González Rato^1^

^1^Hospital Universitario Central de Asturias (HUCA), 33011 Oviedo, España

**Introducción y Objetivos**: La actividad delta rítmica occipital intermitente (OIRDA) definida por ritmos delta de hasta 4 Hz, normalmente en brotes cortos (5 segundos) en regiones posteriores, se considera una actividad electroencefalográfica de buen pronóstico, propia de epilepsias infantiles, como las ausencias típicas. Este ritmo se considera parte de la actividad epileptiforme de estas epilepsias, apareciendo en un porcentaje de los estudios vEEG de estos niños asociada a dicha actividad y posteriormente desaparecen junto con esta. **Material y Métodos**: Revisión de 249 estudios v-EEG con actividad occipital patológica, de la base de datos de vEEG del Hospital Universitario Central de Asturias de más de 12.000 estudios (desde mayo de 2019 a mayo 2025). Se identifica OIRDA en 16 de 31 EEGs realizados a 11 pacientes. De estos, se revisan los informes clínicos de seguimiento y se valora el pronóstico en base a persistencia de crisis, actividad crítica e intercrítica, uso de antiepilépticos y estado basal de estos pacientes. **Resultados**: Los valores demográficos de estos pacientes nos presentan una población infantil con una media de edad de 9 años, 6 niños y 5 niñas, diagnosticados con ausencias típicas, síndrome de Panayiotopoulos, síndrome de Gastaut y epilepsia generalizada idiopática. De estos, el 73% permanecen libres de crisis (de los cuales más del 50% continúan tomando antiepilépticos). La actividad OIRDA, junto con la actividad intercrítica, había desaparecido conjuntamente en el 45% de los EEGs de control de estos pacientes. **Discusión/Conclusiones**: La presencia de OIRDA en el EEG es una actividad poco frecuente propia de la edad pediátrica, presente en un pequeño porcentaje de los síndromes epilépticos infantiles y parece estar relacionada con la actividad epileptiforme de estos. Si bien pueden ser una actividad de un buen pronóstico, son necesarios más estudios, especialmente debido a que las patologías de predominio occipital (como la epilepsia tipo Gastaut) pueden ocasionar interpretaciones erróneas en su identificación.

**Enfermedad de Creutzfeldt-Jakob, Variante de Heidenhain. A Propósito de un Caso**

Natali Faouzi Aboul Hosn Tahmouche^1^, Cielo González Mendonza^1^, Cristina Moreno Jiménez^1^, Raúl Armas Zurita^1^, Fadi Hallal Peche^1^, Mariano Aguilera Vergara^1^, Juan Sebastián Rodríguez Quinchanegua^1^

^1^Hospital Central de la Defensa Gómez Ulla, 28047 Madrid, España

**Introducción y Objetivos**: La enfermedad de Creutzfeldt-Jakob (ECJ) es una encefalopatía transmisible de origen priónico, rápidamente progresiva y letal. Un 80–85% de casos son esporádicos y el 10–15% son genéticos. La variante Heidenhain, se manifiesta inicialmente con síntomas visuales aislados como diplopía, alucinaciones visuales o pérdida visual cortical, lo cual puede retrasar el diagnóstico correcto. **Material y Métodos**: Se presenta el caso de un varón de 60 años que inicia con pérdida visual binocular súbita y visión de destellos amarillos, atribuida inicialmente a cataratas en su país de origen (Mauritania). Evoluciona en el último mes con deterioro neurológico progresivo: inestabilidad de la marcha, lenguaje reducido, disfagia para sólidos y líquidos, incontinencia urinaria, rigidez de extremidades fluctuante y fasciculaciones. A su llegada a España, presentaba bajo nivel de conciencia y rigidez generalizada. **Resultados**: Las pruebas iniciales (TC, angio-TC, analítica sanguínea, tóxicos en orina, radiografía de tórax, ECG) fueron normales. En el primer registro EEG se objetivó un patrón compatible con ictalinterictal continuum (IIC) mostrando GPDs (descargas periódicas generalizadas a <2,5 Hz). El mismo día, tras instauración de tratamiento antiepiléptico, se observa enlentecimiento generalizado y cambio de la persistencia de los GPDs, sin cambios clínicos. Se realizan en total 11 registros de EEG durante los 14 días de ingreso, en los que se observó un patrón establecido de IIC arreactivo a estímulos, que no presenta modificaciones eléctricas ni clínicas pese a tratamiento intensivo en UCI. Tras descartar causas infecciosas, tóxicas, neoplásicas y metabólicas, el análisis del LCR reveló proteínas normales y presencia de proteína 14-3-3, lo que confirmó el diagnóstico de ECJ esporádica probable, variante Heidenhain. **Discusión/Conclusiones**: La variante Heidenhain, caracterizada por síntomas visuales iniciales, puede retrasar el diagnóstico de ECJ y simular entidades tratables. Su reconocimiento precoz permite orientar el estudio.

**Encefalitis Anti NMDA con Catatonia: Resolución con Terapia Electroconvulsiva**

Rosa Altagracia Beriguete Alcántara^1^, Erika Herráez Sánchez^1^, Erika Rolando Agudo Herrera^1^, Miguel Pintor Zamora^1^, Juana Catalina Martínez Ramos^1^, Irene Renovell Bueno^1^, Elena Escario Méndez^1^

^1^Hospital Universitario Rey Juan Carlos (HURJC), 28933 Madrid, España

**Introducción y Objetivos**: La encefalitis por anticuerpos antirreceptor N-metil-D-aspartato (anti- NMDA-R) es una enfermedad autoinmune grave y reversible. Se caracteriza por alteraciones conductuales y psiquiátricas, trastornos del movimiento y crisis epilépticas. El electroencefalograma (EEG) puede mostrar alteraciones inespecíficas, aunque a lo largo de la evolución puede observarse un patrón característico como el delta-brush. El objetivo de este caso es mostrar como el EEG es útil para su diagnóstico, así como el papel de la terapia electroconvulsiva en pacientes refractarios que cursan con catatonia. **Material y Métodos**: Reporte de caso clínico de paciente estudiado en nuestra Unidad de Video-EEG (vEEG). **Resultados**: Varón de 34 años, con antecedentes de consumo de cannabis, con clínica de 1 mes de evolución consistente en depresión, ideas delirantes, alucinaciones visuales y auditivas y, aumento de consumo de tóxicos. Acude a urgencias por intento autolítico y se ingresa en psiquiatría con diagnóstico de brote psicótico, que empeora con neurolépticos, presentando agitación, confusión y agresividad. Tras su ingreso, presenta disautonomía y una crisis epiléptica. Se solicita EEG para descartar la presencia de patología orgánica. En los EEG seriados se observa patrón delta-brush, sin evidenciarse crisis, y se sospecha de encefalitis anti-NMDA. Se confirma positividad de anticuerpos anti-NMDA en suero y LCR. Se inicia tratamiento con corticoterapia e inmunoglobulinas, y rituximab por escasa respuesta a lo previo. A pesar de ello, presenta fluctuación clínica y marcada catatonia, por lo que se realiza terapia electroconvulsiva (TEC) con importante mejoría clínica a partir de la tercera sesión. **Discusión/Conclusiones**: La encefalitis anti-NMDA suele cursar con clínica psicótica, siendo necesario realizar diagnóstico diferencial con cuadro psiquiátrico agudo. El EEG puede orientar mediante hallazgos como el delta-brush, aunque el diagnóstico definitivo requiere la detección de anticuerpos. En casos de catatonia refractaria, la TEC es una opción eficaz.

**EEG Privación de Sueño en Trastorno del Espectro Autista y Epilepsia: Experiencia en el Hospital de Manises**

Ángela María Suero Cubilete^1^

^1^Hospital de Manises, 46930 Valencia, España

**Introducción y Objetivos**: Introducción: El Trastorno del Espectro Autista (TEA) es un trastorno del neurodesarrollo caracterizado por déficits persistentes en la comunicación, alteraciones en la reciprocidad social y patrones de comportamiento restringidos y repetitivos. Su etiología es multifactorial, con alta heterogeneidad clínica y genética. La asociación entre TEA y epilepsia ha sido ampliamente documentada, con una prevalencia de epilepsia en TEA estimada en un 12,1%. Hasta un 60% de estos pacientes pueden presentar anomalías electroencefalográficas (EEG) sin crisis clínicas aparentes, lo que resalta la relevancia del EEG como herramienta diagnóstica. Las anomalías más frecuentes incluyen actividad epileptiforme focal o multifocal, y su detección precoz es clave para reducir la morbilidad asociada. Objetivo: Evaluar la sensibilidad y especificidad del EEG con privación de sueño en la detección de epilepsia en pacientes con TEA de nuestro hospital. **Material y Métodos**: Estudio observacional, retrospectivo, de pacientes con diagnóstico de TEA atendidos en nuestra unidad de Neurofisiología entre 2023 y 2025. Se clasificaron según el tipo de EEG realizado: basal o tras privación de sueño. Se registró la presencia o ausencia de actividad epiléptica en el EEG. Se valora la sensibilidad y especificidad de la prueba en relación con los hallazgos electroencefalográficos. **Resultados**: La muestra en tres años fueron 35 pacientes, remitidos por Neuropediatría de los cuales 22 realizaron EEG con privación de sueño y 13 EEG basal. Se detectaron 10 EEG presentaron hallazgos patológicos compatibles con actividad epiléptica en el grupo con privación de sueño. El EEG con privación de sueño presentó una sensibilidad del 100% y una especificidad del 88,5%, mientras que el EEG basal identificó sólo 1 caso patológico con una sensibilidad del 20% y una especificidad del 100%. **Discusión/Conclusiones**: El EEG basal, aunque altamente específico, tiene una sensibilidad baja en la detección de actividad epiléptica en pacientes con TEA. El EEG con privación de sueño demostró una superior capacidad diagnóstica, lo que respalda su uso preferente en la evaluación neurofisiológica de esta población, especialmente en presencia de sospecha clínica o antecedentes genéticos relevantes.

**“Cuanta Emoción”, Epilepsia Refleja. A Propósito de un Caso**

Diego Francisco Peña Olaya^1^, Ana Gómez Menéndez^1^, Beatriz García López^1^, Alina Havrylenko Vynogradnyk^1^, Joseba Soto Ibáñez^1^, Boris Alfonso Orozco Jiménez^1^, Gricela María Sorto Bueso^1^

^1^Hospital Universitario de Burgos (HUBU), 09006 Burgos, España

**Introducción y Objetivos**: Epilepsias reflejas. Grupo de síndromes raros e infrecuentes de difícil manejo, caracterizado por crisis desencadenadas por un estímulo heterogéneo aferente (simple o complejo). Las crisis pueden ser de etiología y semiología variable, abarcando no solo manifestaciones motoras, sensoriales o cognitivas. Así mismo, pueden ser o no, el único tipo de crisis o coexistir con una epilepsia de base; lo que complica aún más su diagnóstico y manejo. El origen de estas crisis se asocia con la activación de redes neuronales anatómica y fisiológicamente funcionales, que coinciden con áreas de hiperexcitabilidad cortical, que consiguen reclutar la actividad neuronal provocando una crisis epiléctica. El tiempo entre el estímulo detonante y la aparición de cambios en el EEG puede ser de presentación inmediata o retardada, dependiendo del estímulo y la sensibilidad del paciente. **Material y Métodos**: Exponemos el caso de una paciente con crisis epilépticas reflejas, inducida por emociones (estrés o angustia), haciendo un diagnóstico diferencial con EPNE (Eventos paroxísticos no epilépticos). **Resultados**: Mujer de 40 años, antecedentes de Estatus epiléptico superrefractario de novo (NORSE/FIRES) en el verano de 2016 en un contexto infeccioso. En marzo de 2017 coincidiendo con bajada de fármacos anticrisis, comenzó a presentar episodios paroxísticos polimorfos con semiología auditiva y sensitiva de hemicuerpo izquierdo, dificultad para hablar y movilizar la hemicara izquierda. Estos se desencadenaban por emociones. Los vEEG convencionales fueron normales, se realizó vEEG prolongado, para hacer diagnostico diferencial entre EPNE Vs crisis refleja, registrando 4 crisis epilépticas. secundario a esto se realiza reajuste farmacológico. **Discusión/Conclusiones**: En general las crisis reflejas, son poco prevalentes, dado su variable morfología, así como su aparición concomitante o no, con una epilepsia de base; dadas estas características se hace necesario un estudio especifico, para poder realizar un correcto diagnóstico y un adecuado manejo.

**Epilepsia con Mioclonías Palpebrales: El Papel Clave del Vídeo-EEG en el Proceso Diagnóstico**

Rosa Altagracia Beriguete Alcántara^1^, Erika Herráez Sánchez^1^, Luigi Pietro Carlo Unda^1^

^1^Hospital Universitario Rey Juan Carlos (HURJC), 28933 Madrid, España

**Introducción y Objetivos**: La epilepsia con mioclonías palpebrales, conocida como síndrome de Jeavons, es un tipo de epilepsia generalmente farmacorresistente caracterizada por la presencia de mioclonías palpebrales, con o sin ausencias, pudiendo asociar otros tipos de crisis como tónico-clónicas generalizadas (CTCG). Es una entidad poco prevalente, aunque se considera infradiagnosticada, ya que las mioclonías palpebrales pueden no ser percibidas o incluso ser confundidas con tics, siendo inicialmente diagnosticada como otro tipo de epilepsia o eventos paroxísticos no epilépticos. **Material y Métodos**: Se analizan variables demográficas, clínicas y electroencefalográficas de una serie de 7 casos diagnosticados de epilepsia con mioclonías palpebrales en nuestra unidad de Vídeo-EEG (v-EEG). **Resultados**: De los 7 pacientes, el 85,7% son mujeres. La edad media de debut de epilepsia fue de 9,4 años. La sospecha clínica inicial fue de crisis tónico-clónica generalizada en el 28,6%, crisis de ausencia en 28,6%, eventos paroxísticos no epilépticos (EPNE) en el 28,6% y de mioclonías palpebrales en el 14,3%. En el 71,4% las mioclonías palpebrales no fueron percibidas por los pacientes ni familiares. La edad media de diagnóstico de epilepsia con mioclonías palpebrales de 13,6 años. En un 85,7% de los casos se cambió el diagnóstico inicial tras la realización del v-EEG, excepto en un paciente que acudió con el diagnóstico ya realizado. **Discusión/Conclusiones**: La epilepsia con mioclonía palpebrales es una entidad infradiagnosticada. La mayoría de los pacientes o familiares no reconocen los eventos como crisis, y la sospecha clínica inicial suele variar entre ausencias infantiles, CTCG o EPNE. Ante estos casos, se plantea la importancia de investigar de forma exhaustiva por este tipo de crisis, así como realizar un correcto protocolo de estudio en el v-EEG, ya que es preciso un correcto diagnóstico dadas características pronósticas de esta entidad.

**Clasificación de Patrones Electroencefalográficos con IA a Partir de Imágenes de Atlas: Estudio Piloto**

Gisel Fabiola Montoya Aguirre^1^, Mauricio Alejandro Echazú Suaznabar^2^

^1^Hospital Universitario Sant Joan de Reus, 43204 Tarragona, España

^2^Consultoria independiente

**Introducción y Objetivos**: La interpretación del electroencefalograma (EEG) requiere alta especialización, y la incorporación de herramientas de inteligencia artificial (IA) podría facilitar el reconocimiento de patrones relevantes. Este estudio piloto tuvo como objetivo evaluar la viabilidad de modelos de IA para clasificar imágenes EEG extraídas de atlas clínicos en cuatro categorías: normal, variantes benignas, artefactos y patrones patológicos. **Material y Métodos**: Se recopilaron 115 imágenes de EEG clasificadas manualmente, algunas marcadas con recuadros que destacaban las regiones de interés. Se entrenaron dos modelos: una red neuronal convolucional simple (CNN) desde cero y un modelo MobileNetV2 preentrenado mediante transferencia de aprendizaje. El conjunto se dividió en entrenamiento (80%) y validación (20%). Se analizaron métricas de precisión y matrices de confusión. **Resultados**: La CNN alcanzó una precisión del 80,7% en entrenamiento y 68,2% en validación. MobileNetV2 mostró un mejor desempeño, con una precisión del 85% en entrenamiento y 72% en validación. En ambos modelos, los errores se concentraron entre EEG normales, variantes benignas y patrones patológicos. Las clases de artefactos y patológicos se clasificaron con mayor estabilidad. **Discusión/Conclusiones**: A pesar del número limitado de imágenes, los modelos lograron diferenciar patrones EEG con una precisión significativa. MobileNetV2 fue superior a la CNN básica, especialmente en la generalización. Este trabajo demuestra la viabilidad de entrenar modelos IA con imágenes clasificadas de atlas, y plantea la posibilidad de extender su aplicación a entornos clínicos reales tras la correspondiente evaluación ética.

**A Propósito de un Caso: Síndrome de Alicia en el País de las Maravillas con Correlato EEG**

Andrea Gómez Moroney^1^, María Camila Bautista Villavicencio^2^, Carlos Castañeda Cabrero^1^, Sonia Montilla Izquierdo^1^, Clara Fernández Cortés^1^, Madeleyn Rodríguez Jiménez^1^, Cristina Fernández García^1^

^1^Hospital Universitario La Moraleja, 28050 Madrid, España

^2^Hospital Universitario La Paz, 28046 Madrid, España

**Introducción y Objetivos**: El Síndrome de Alicia en el País de las Maravillas (SAPM) es un trastorno infrecuente caracterizado por episodios de distorsión visuoespacial y alteraciones en la propiocepción. Es más frecuente en edad pediátrica y puede asociarse a infecciones virales, migraña y, en menor proporción, a epilepsia. Los estudios de neuroimagen suelen ser normales, sin embargo, el electroencefalograma (EEG) puede mostrar hallazgos característicos como la presencia de ondas lentas en regiones occipitales o temporoparietales en ausencia de actividad epileptiforme, lo que podría reflejar una disfunción cortical transitoria. El objetivo del estudio es describir un caso en paciente pediátrico con sospecha de SAPM y correlato EEG. **Material y Métodos**: Paciente en edad pediátrica con episodios autolimitados de distorsión visuoperceptiva descritos como alteraciones en el tamaño, forma y distancia de los objetos. Se realizó exploración neurológica, resonancia magnética (RM) craneal, y EEG de vigilia siguiendo el Sistema Internacional 10–20. **Resultados**: Varón de 10 años sin antecedentes neurológicos de interés, que consulta por episodios autolimitados de metamorfopsia, sin pérdida de consciencia ni otros síntomas acompañantes. La exploración neurológica y la RM fueron normales. El EEG mostró la presencia de anomalías focales en forma de ondas lentas sobre ambas regiones occipitales, sin actividad epileptiforme sobreañadida, coincidiendo con los episodios de distorsión visuoespacial. Estos hallazgos se han descrito previamente en pacientes con SAPM. **Discusión/Conclusiones**: Si bien los hallazgos EEG en el SAPM son infrecuentes e inespecíficos, el EEG puede aportar valor en el proceso diagnóstico, especialmente si se identifican alteraciones como las descritas, lo que puede ayudar a descartar otras etiologías como la epiléptica o la estructural. De esa manera, se puede evitar la realización de otras exploraciones innecesarias, contribuyendo a la tranquilidad del paciente y de su entorno familiar ante un cuadro llamativo, pero de curso generalmente benigno.

**Diferentes Escenarios del Estatus Epiléptico Eléctrico Durante el Sueño (ESES): A Proposito de 3 Casos**

Pilar Rosario Martínez Martínez^1^, Sofía Ortigosa Gómez^1^, Carmen María Garnés Sánchez^1^, Julián Vázquez Lorenzo^1^, Víctor Hugo Rubio Suárez^1^, Justo Marín Noguera^1^, Reetika Mukesh Baharani Baharani^2^

^1^Hospital Clínico Universitario Virgen de la Arrixaca, 30120 Murcia, España

^2^Hospital Morales Meseguer, 30008 Murcia, España

**Introducción y Objetivos**: El DEE-SWAS (Developmental and Epileptic Encephalopathy Sleep with Spike-Wave Activation in Sleep) y EE-SWAS (Epileptic Encephalopathy with Spike-Wave Activation in Sleep) se tratan de un espectro encefalopático poco frecuente de la infancia caracterizado por cursar, clínicamente con deterioro neurocognitivo y psicológico en diversos grados, y por un patrón EEG de punta-onda casi continua durante el sueño (ESES: Electrical Status Epilepticus in Sleep). El ESES se clasifica en: típico, el índice punta-onda es > o igual al 85% en sueño NREM, o atípico, si es >85% y >50%. La etiología puede ser genética, estructural, metabólica o idiopática. La efectividad del tratamiento se mide por la mejoría clínica, y la proporción de actividad punta-onda en los registros de EEG nocturnos. **Material y Métodos**: Estudio retrospectivo de 3 pacientes pediátricos con diagnóstico de DEE/EE-SWAS, mediante EEG seriados con sueño nocturno. **Resultados**: Los 3 pacientes presentan regresión cognitiva, sobre todo en el área del lenguaje y EEG compatible con ESES. Los 3 han sido tratados con fármacos antiepilépticos junto a benzodiacepinas. Caso 1: niño de 4 años, antecedente de debut a los 2 años con crisis febril compleja, y foco inicial centro-temporo-parietal bilateral y línea media sagital. ESES típico. Buena respuesta al tratamiento. Caso 2: niña de 6 años, con antecedente de hemimegalencefalia. ESES atípico. Mala respuesta al tratamiento, se lleva a comité de epilepsia. Caso 3: niña de 8 años, con antecedente de lesiones isquémicohemorrágicas en sustancia blanca subcortical bihemisféricas y pequeños focos de sangrado extraaxial en fosa posterior en periodo perinatal, resueltos en la actualidad. ESES típico. Mala respuesta al tratamiento, se inicia corticoterapia. **Discusión/Conclusiones**: El EEG es fundamental para el diagnóstico y seguimiento del ESES, de preferencia con registro de sueño nocturno con el fin de poder realizar una cuantificación completa de las anomalías epileptiformes.

**Cuando el Vídeo-EEG es Clave. Diagnóstico Diferencial del Deterioro Rápidamente Progresivo con Clínica Psicótica**

Elena Escario Méndez^1^, Erika Herráez Sánchez^1^, Adrián García Hernández^2^, Luigi Pietro Carlo Unda^1^, Rosa Altagracia Berigüete Alcántara^1^, Rolando Agudo Guerra^1^

^1^Hospital Universitario Rey Juan Carlos, 28933 Madrid, España

^2^Hospital Universitario General de Villalba, 28400 Madrid, España

**Introducción y Objetivos**: El deterioro cognitivo rápidamente progresivo (DCRP) implica un rápido declive cognitivo que incluye en su diagnóstico diferencial un espectro heterogéneo de entidades como infecciones del SNC, causas tóxico-metabólicas, enfermedades neurodegenerativas rápidamente progresivas, epilepsia o trastornos psiquiátricos, entre otras. El objetivo es describir el abordaje diagnóstico de una paciente con DCRP destacando los hallazgos electroclínicos que obligaron a replantear la orientación del caso. **Material y Métodos**: Reporte de un caso clínico. Detalle del proceso diagnóstico y revisión de la literatura. **Resultados**: Mujer de 79 años con antecedente de depresión. Acude a urgencias tras 3 meses de decaimiento anímico y alteraciones conductuales. Ingresa por síndrome confusional evolucionando a un DCRP con episodios de heteroagresividad. El curso es fluctuante con fases de delirium e hipoactividad. Sufre una crisis epiléptica y un pico febril realizándose un videoelectroencefalograma (v-EEG) con actividad epileptiforme, registrando episodios de dudosa etiología. Se extrae líquido cefalorraquídeo, sin hallazgos patológicos y con anticuerpos negativos (incluyendo NMDA). Inicia tratamiento inmunomodulador pero ante la ausencia de mejoría se interconsulta a psiquiatría diagnosticándose depresión mayor con síntomas psicóticos. Pese a una mejoría parcial con tratamiento psicofarmacológico y anticrisis, los episodios persisten. Sufre nueva crisis tónico-clónica realizándose neuroimagen que es normal y nuevo v-EEG registrándose un evento crítico que sugiere un origen epiléptico de la clínica psicótica. Se completa estudio etiológico con sospecha de encefalitis autoinmune y se evidencia tumor mamario. **Discusión/Conclusiones**: Se expone un caso poco frecuente que muestra la dificultad diagnóstica de pacientes psiquiátricos con síntomas neurológicos y muestra el valor del estudio v-EEG. La relevancia de un diagnóstico precoz es esencial y radica en su posible reversibilidad, ya que las posibilidades de éxito del tratamiento son mayores.

**Patrones Electroencefalográficos en Síndromes Cerebrales Asociados a MOGAD**

Rudy Jemina Chamorro Portilla^1^, N. Navarrete Páez^1^, A. Nieto Jiménez ^1^, M. Cobo Moreno^1^, L. Lorente Remiz^1^, C. Cazorla Cabrera^1^, A. Galdón Castillo^1^

^1^Hospital Universitario Virgen de las Nieves, 18012 Granada, España

**Introducción y Objetivos**: Introducción: La enfermedad asociada a anticuerpos contra la glicoproteína oligodendrocítica de la mielina (MOGAD) es un trastorno inflamatorio desmielinizante con fenotipo clínico variable. Recientemente se han descrito crisis epilépticas aisladas como presentación inicial de MOGAD, lo que resalta la utilidad del EEG en el diagnóstico diferencial. Objetivo: Revisar la evidencia sobre hallazgos electroencefalográficos síndromes cerebrales por MOGAD comparados con otras entidades inflamatorias del SNC, y describir dos casos en nuestro centro. **Material y Métodos**: Métodos: Revisión sistemática en Cochrane, EMBASE, PubMed y Scopus sobre hallazgos EEG en MOGAD, utilizando los términos: Electroencephalogram AND seizure AND encephalitis AND myelin oligodendrocyte glycoprotein antibody associated disease. **Resultados**: Los síndromes encefálicos por anticuerpos anti-MOG suelen cursar con crisis focales o focales a bilaterales. La AIC suele ser escasa, predominando en lóbulo temporal, aunque también se ha descrito en lóbulos parietal y occipital. Se presentan dos casos (10 y 11 años) con encefalomielitis diseminada por MOGAD y crisis convulsivas con enlentecimiento difuso en el EEG basal. Uno de los casos mostró actividad epileptiforme temporal izquierda y actividad lenta bifrontal. **Discusión/Conclusiones**: Discusión: A diferencia de otras encefalitis autoinmunes, con fenotipo frecuente de encefalitis límbica, MOGAD suele cursar con encefalitis cortical y se asocia más frecuentemente con hallazgos epileptiformes en regiones temporal, parietal y occipital, aunque sin patrones específicos definidos. Nuestros casos coinciden con lo reportado en la literatura. Conclusiones: El EEG en encefalitis por MOGAD puede mostrar características útiles para el diagnóstico diferencial. Se requieren estudios específicos que profundicen en los hallazgos neurofisiológicos en esta entidad.

**Estatus Epiléptico Eléctrico del Sueño: Nuestra Experiencia**

Marina Murillo Martínez^1^, Andrea Sánchez Femenía^1^, Lydia Navarro Ruiz^1^, Pau Giner Bayarri^1^, Ana Victoria Marco Hernández^1^

^1^Hospital Universitario Doctor Peset, 46017 Valencia, España

**Introducción y Objetivos**: El estatus epiléptico eléctrico del sueño (ESES) es un patrón EEG caracterizado por descargas epileptiformes tipo punta-onda lenta persistente durante el sueño no REM. Puede presentarse dentro de la Encefalopatía Epiléptica con activación de punta-onda en el sueño (EE-SWAS), entidad idiopática infantil caracterizada por deterioro cognitivo junto al patrón ESES; en otras epilepsias idiopáticas o secundario a lesiones estructurales cerebrales. Actualmente no existe consenso sobre el abordaje diagnóstico ni terapéutico para los pacientes con ESES. Por ello, el objetivo de este estudio es conocer cómo se manifiesta el ESES en nuestra población, el manejo recibido y su evolución. **Material y Métodos**: Estudio observacional retrospectivo mediante uso de historias clínicas de pacientes pediátricos diagnosticados de ESES en nuestro centro entre 2012 y 2023. **Resultados**: Se obtuvo una muestra de 23 pacientes. Edad media al diagnóstico 6,2 años (rango 2–16). 39% eran de etiología idiopática, 61% presentaban alteraciones estructurales. Las manifestaciones clínicas fueron crisis epilépticas (73%), alteración del lenguaje (43%), alteración del comportamiento (21%) y alteración motora (29%). El 78% tenían EEGs previos al diagnóstico, 56% con actividad epiléptica. El trazado ESES fue de punta onda generalizada en el 52% casos, resto focal. El principal método diagnóstico fue la siesta diurna (registro EEG 2–3 horas) (57%) seguido de monitorización de 24 horas (39%). Los fármacos más utilizados fueron el valproato, clobazam y levetiracetam. La remisión en el EEG fue de un 78% (rango 3–27 meses) y la remisión clínica de un 52%. **Discusión/Conclusiones**: El ESES es infrecuente y requiere una alta sospecha diagnóstica. La clínica principal fueron las crisis epilépticas. La realización de estudios EEG que incluyan periodos de sueño es fundamental para el diagnóstico, siendo la siesta diurna y la monitorización de 24 horas las principales herramientas utilizadas en nuestro medio. Es necesario estandarizar el estudio y manejo de estos pacientes.

**Paciente con Displasia Septo-Óptica y Punta-Onda Lenta Continua Asimétrica en un Estudio v-EEG de Duración Intermedia**

Diana Peñalver Espinosa^1^, Estefanía García Luna^1^, Francisco Granados López^1^, Eugenio Barona Giménez^1^, Andrés García Verdú^1^, Luis García Alonso^1^, Roberto López Bernabé^1^

^1^Hospital General Universitario Reina Sofía, 30003 Murcia, España

**Introducción y Objetivos**: La punta onda lenta continua en sueño (POCS) es una encefalopatía epiléptica infantil. El diagnóstico es difícil en pacientes con discapacidad intelectual por la confluencia de síntomas en lenguaje y comportamiento. Se propuso SWI ≥85% pero se han propuesto porcentajes inferiores. **Material y Métodos**: Varón de 16 años con esquisencefalia de labio abierto hemisférica derecha, hipoplasia de nervios ópticos, ausencia de septum pellucidum y déficit de ADH central (displasia septo-óptica), déficit visual, hemiparesia izquierda, discapacidad intelectual, retraso psicomotor y epilepsia focal. Ausencia de lenguaje. Toma topiramato 75 mg cada 12 horas. **Resultados**: En v-EEG de duración intermedia con privación de sueño y registro de sueño diurno se observa, en vigilia, una actividad de fondo enlentecida, sin reactividad a la apertura y cierre ocular y descargas de complejos punta onda lenta sobre regiones parietotemporooccipitales derechas y línea media con tendencia a difundir a todo el hemisferio derecho y regiones homólogas de izquierdo. De forma ocasional, muestran tendencia a generalizar manteniendo predominio derecho. No muestran correlato clínico. Durante el sueño NREM, se observan complejos punta onda lenta continuos con índice Spike Wave in Sleep (SWI) >85%, que se exteriorizan sobre hemisferio derecho y, en ocasiones, generalizadas sobre ambos hemisferios con predominio derecho, sin correlato clínico. Se observan grafoelementos de sueño en hemisferio izquierdo; en hemisferio derecho, la valoración es limitada por la incidencia de anomalías epileptiformes. **Discusión/Conclusiones**: En este caso, la ausencia de cuerpo calloso dificulta la propagación de la actividad epileptiforme al hemisferio izquierdo, limitando la expresión simétrica. ¿Es suficiente un registro de sueño diurno con sueño NREM para el diagnóstico? El SWI en sueño diurno no fue diferente al del primer ciclo de sueño nocturno. El estudio diurno puede ser específico y suficiente para el diagnóstico; la limitación es que el sueño puede ser más fragmentado y superficial.

**Desafíos Diagnósticos y Terapéuticos en un Caso Pediátrico Complejo de Epilepsia Focal Farmacorresistente**

Laura Menéndez Rúa^1^, Shijia Li^2^, Hyun Suk Oh Kim^1^, Pilar Tirado Requero^3^, Jorge Zamorano Fernández^3^, Arturo Ugalde Canitrote^4^

^1^Hospital Gómez Ulla, 28047 Madrid, España

^2^Hospital Universitario Miguel Servet, 50009 Zaragoza, España

^3^Hospital La Paz, 28046 Madrid, España

^4^Hospital la Paz, Facultad de Medicina, Universidad Francisco de Vitoria, 28223 Pozuelo de Alarcón, Madrid, España

**Introducción y Objetivos**: La epilepsia focal farmacorresistente en edad pediátrica puede constituir un desafío diagnóstico y terapéutico. Entre sus etiologías se encuentran la Displasia Cortical Focal (DCF) y, rara vez, la Encefalitis de Rasmussen (ER), encefalopatía inmunomediada progresiva que afecta un hemisferio cerebral, caracterizada por crisis focales, deterioro neurológico progresivo y hallazgos radiológicos típicos. Ambas entidades pueden compartir características clínicas, de imagen y electroencefalográficas, lo que dificulta su diferenciación. **Material y Métodos**: Se describe el caso de un niño que debuta a los 9 años con crisis focales y progresión a convulsiones bilaterales. Es derivado a nuestro hospital 9 meses después, con crisis frecuentes subintrantes de desviación izquierda de la mirada, alteración parcial de consciencia y a veces clonías en extremidades izquierdas, requiriendo ingreso en UCI, con difícil control farmacológico (medicación anticrisis e inmunomoduladores). **Resultados**: La RM sugirió posible ER y el PET-TAC evidenció hipometabolismo hemisférico derecho. La vídeoelectroencefalografía mostró probable origen de crisis en córtex posterior derecho. Se decidió estudio estéreo-electroencefalográfico con estimulación eléctrica y termocoagulación de zona epileptógena, con resultado de control parcial de crisis. Siete meses después, inició posible epilepsia focal continua motora del pie izquierdo. Dos meses después se realizó corticectomía parietal derecha guiada por electrocorticografía intraoperatoria, con mejoría significativa de las crisis iniciales y discreta mejoría de los movimientos en pie. La anatomía patológica mostró DCF tipo Ia. **Discusión/Conclusiones**: El caso ilustra la dificultad del diagnóstico diferencial entre DCF y ER, así como la necesidad de un manejo interdisciplinar experto, urgente y preciso, en epilepsias complejas en el paciente pediátrico, guiado por neuroimagen y herramientas neurofisiológicas sofisticadas, con abordajes quirúrgicos a medida.

**Migraña Confusional Aguda en la Infancia: A Propósito de un Caso**

Paula Villacis Palacios^1^, Beatriz Martín Bujanda^1^, Juan Arcocha Aguirrezabal^1^

^1^Hospital Universitario de Navarra, 31008 Pamplona, España

**Introducción y Objetivos**: La migraña confusional aguda (MCA) es una forma de migraña complicada en la infancia, de probable mecanismo isquémico en territorios dependientes de arterias cerebral posterior (ACP) y media (ACM), que se presenta como un estado confusional agudo transitorio, con sintomatología variable en forma de desorientación, somnolencia, agitación, afasia y pródromos visuales asociados o no a cefalea, con recuperación completa del episodio. **Material y Métodos**: Niño de 11 años que consulta por episodio de cefalea intensa de inicio brusco, vómitos, disartria y somnolencia de 1 hora de evolución. No refiere traumatismo, cuadro respiratorio ni gastrointestinal previos, ni episodios similares. No antecedentes familiares de migrañas o epilepsia. En la exploración destaca disartria, bradipsiquia, confusión y somnolencia (Glasgow 13). Se realiza Escala de PedNHISS con resultado de 2, y se activa código ictus. **Resultados**: Entre los exámenes complementarios, se realiza analítica de sangre normal y tóxicos en orina negativos. En la angioRM se objetiva disminución del calibre y señal de las ramas periféricas de la ACM y ACP izquierda, y en RM de perfusión oligohemia hemisférica izquierda comparativa con vasoespasmo distal. Se realiza EEG en las primeras 24 h con actividad lenta intermitente hemisférica izquierda, sin grafoelementos epileptiformes u otras anomalías asociados. A las 5–6 horas presentó remisión completa de la focalidad neurológica, y previo al alta se realiza EEG de control con normalización del registro. **Discusión/Conclusiones**: La MCA constituye una entidad clínica poco común, de pronóstico favorable, sin déficit neurológico residual. La realización de EEG en las primeras 24 horas resulta útil para el diagnóstico y permite excluir otras patologías de origen vascular, epiléptico, neoplásico, infeccioso o tóxico/metabólico.

**Valor del EEG en el Diagnóstico de Encefalitis: Revisión de una Serie de Casos**

Olimpia García Rico^1^

^1^Hospital Universitario Clínico de Santiago de Compostela, 15706 Santiago de Compostela, A Coruña, España

**Introducción y Objetivos**: Describir los hallazgos electroencefalográficos (EEG) en pacientes con sospecha de encefalitis entre junio de 2024 y junio de 2025, con especial atención a los casos confirmados de encefalitis herpética (VHS-1). **Material y Métodos**: Estudio retrospectivo de 11 EEG realizados por sospecha de encefalitis. Se recogieron datos clínicos, etiológicos y hallazgos EEG. Las indicaciones fueron alteración del nivel de conciencia y sospecha clínica de origen infeccioso o autoinmune. **Resultados**: Cuatro pacientes fueron diagnosticados de encefalitis por VHS-1, confirmados por PCR. En tres de ellos se registraron LPDS, principalmente en regiones temporales. Los siete EEG restantes, en pacientes con sospecha inicial de encefalitis pero sin confirmación virológica, presentaban etiologías diversas o no concluyentes (encefalitis autoinmune, NMO, encefalopatía hepática, meningitis bacteriana o causa desconocida), mostrando lentificación difusa, brotes lentos focales, asimetrías lentas o trazados normales. **Discusión/Conclusiones**: Los LPDS fueron un hallazgo frecuente (75%) en los casos de encefalitis herpética. Su presencia en pacientes con clínica compatible puede orientar precozmente el diagnóstico. En encefalitis no herpéticas, los EEG fueron menos específicos pero útiles para seguimiento y diagnóstico diferencial. El EEG es una herramienta clave en el abordaje de encefalitis, especialmente de etiología por VHS. La presencia de LPDS permite un diagnóstico temprano de esta entidad, así como un mejor ajuste terapéutico en estos pacientes.

**Hiperglicinemia no Cetósica: Desafío Diagnóstico en las Encefalopatías Metabólicas Neonatales: A Propósito de 2 Casos**

Gricela María Sorto Bueso^1^, María Carmen Lloria Gil^1^, Ana Isabel Gómez Menéndez^1^, Alina Havrylenko Vynogradnyk^1^, Boris Alfonso Orozco Jiménez^1^, Diego Francisco Peña Olaya^1^, Joseba Soto Ibañez^1^

^1^Hospital Universitario de Burgos (HUBU), 09006 Burgos, España

**Introducción y Objetivos**: La Hiperglicinemia no cetósica es un trastorno metabólico congénito raro, con herencia autosómica recesiva, que afecta la degradación de la glicina, generando un acúmulo en los tejidos. La principal afectación es a nivel del sistema nervioso central debido a la hiperestimulación de los receptores NMDA. **Material y Métodos**: Se trata de 2 varones que a las 24 horas de vida presentan hipoactividad, poca reactividad, llanto débil, succión débil y pupilas mióticas. Se inicia estudio descartando las causas más frecuentes (estructurales, infecciosas o tóxicas) y se realiza un primer vEEG que reporta actividad brote supresión. Ante sospecha clínica de patología metabólica, se solicitan niveles de glicina en plasma y LCR. A las 48 horas de vida inician con mioclonías de las extremidades, se solicita vEEG control que registra actividad compatible con crisis epilépticas por lo que se inicia tratamiento con Fenobarbital. Se realizan varios vEEG durante el ingreso de ambos pacientes, obteniendo trazados variables (supresión/crisis), reflejando el deterioro neurológico del cuadro clínico. **Resultados**: Se reciben Resultados: de los niveles de glicina, obteniendo en ambos casos un cociente glicina LCR/plasma patológico (>0,08). **Discusión/Conclusiones**: La presentación clínica de la Hiperglicinemia no cetósica varía entre complejos y diversos fenotipos, siendo la más frecuente la forma grave que inicia en los primeros días de vida con hipotonía, espasmos mioclónicos, apneas, letargia y coma, con un pronóstico muy desfavorable a corto o mediano plazo. En estos casos el vEEG cobra importancia para la identificación precoz de las crisis epilépticas y el seguimiento de la afectación neurológica.

**Epilepsia Farmacorresistente y estereoEEG: Análisis Clínico de una Cohorte de Pacientes**

Antonio Jesús Nieto Jiménez^1^, María Isabel Navarrete Paez^1^, Rudy Jemina Chamorro Portilla^1^, Miguel Cobo Moreno^1^, Cristina Cazorla Cabrera^1^, M. Teresa Ortega León^1^, Alberto Galdón Castillo^1^

^1^Hospital Universitario Virgen de las Nieves, 18012 Granada, España

**Introducción y Objetivos**: El estudio con estereoencefalografía (estereoEEG) se ha convertido en una herramienta relevante dentro del proceso prequirúrgico en pacientes con epilepsia farmacorresistente. Dada su naturaleza invasiva, su indicación debe basarse en criterios bien definidos. Se analiza la experiencia adquirida en nuestro centro con este tipo de pacientes. **Material y Métodos**: Se revisó una cohorte de 48 pacientes evaluados mediante estereoEEG en el contexto de cirugía de epilepsia. Se consideraron diversas variables clínicas y paraclínicas disponibles en el momento del estudio: año de inicio de la epilepsia, duración de la enfermedad, hallazgos en pruebas de imagen, uso de pruebas funcionales adicionales y características técnicas de la implantación de electrodos. **Resultados**: La mediana de edad de inicio de la epilepsia fue de 8 años (rango 0–46 años), 20 pacientes eran mujeres (42%) y 28 eran hombres (58%). La RM cerebral fue negativa en 23 pacientes (48%); 80% contaron con PET cerebral como parte del estudio. Se implantaron 338 electrodos (mediana de 7 electrodos por paciente, rango 4–14), con un 60% de implantaciones multilobares. De los 48 pacientes, el 68% fueron intervenidos, bien quirúrgicamente o mediante termocoagulación; de este grupo, el 79% presentó bien una disminución de la frecuencia de crisis, de su intensidad y, en algunos casos, la remisión completa de la enfermedad, en la última actualización de su seguimiento. **Discusión/Conclusiones**: La experiencia recogida respalda el valor del estereoEEG como herramienta diagnóstica en el contexto del estudio prequirúrgico de la epilepsia. Su implementación ha permitido orientar decisiones terapéuticas con una tasa de intervención y evolución clínica similar a la reportada en otros centros. Estos Resultados: refuerzan su integración dentro del abordaje habitual en unidades de cirugía de la epilepsia.

**Descargas Periódicas en Pacientes Críticos, Implicaciones Pronósticas**

Alexandra Desiree Dávila Andrade^1^

^1^Hospital Universitario Central de Asturias, 33011 Oviedo, España

**Introducción y Objetivos**: Las descargas periódicas (PDs) en el EEG de pacientes críticos han sido asociadas con hiperexcitabilidad cerebral, estado epiléptico no convulsivo y alta mortalidad. La estandarización de su nomenclatura por la American Clinical Neurophysiology Society (ACNS, 2021) ha permitido caracterizar mejor su implicancia clínica. Aún persiste incertidumbre sobre la relación entre sus subtipos y el desenlace clínico. **Material y Métodos**: Estudio observacional retrospectivo en el que se han evaluado en 510 EEGs de pacientes en UCI registrados entre enero 2020 y abril 2025, obteniendo 23 registros con PDs. Se recogieron variables clínicas (edad, sexo, diagnóstico, comorbilidades, mortalidad) y EEG (actividad basal, localización de PDs, continuidad, reactividad y presencia de actividad epileptiforme). Se utilizó la prueba de chi-cuadrado para evaluar la asociación entre las variables y la mortalidad, con un valor de *p* < 0,05 como estadísticamente significativo. **Resultados**: La mortalidad fue del 69,6% (n = 16). Se halló asociación significativa con la ausencia de reactividad EEG (*p* = 0,0358), un patrón discontinuo (*p* = 0,0359) y un pronóstico EEG malo (*p* = 0,0009). El 94% de los pacientes fallecidos presentaron actividad basal suprimida o hipovoltada. Aunque sin significación estadística, el 44% de los fallecidos tenían una patología neurológica como diagnóstico principal. Variables como sexo, comorbilidades, diagnóstico, localización de PDs o presencia de actividad epileptiforme no mostraron asociaciones significativas. **Discusión/Conclusiones**: Esta cohorte de pacientes presentó una alta tasa de mortalidad. Determinadas características del EEG, como la ausencia de reactividad, un pronóstico definido como malo y un patrón discontinuo se asociaron significativamente con mayor mortalidad en paciente críticos con PDs. Se requiere mayor tamaño muestral y grupos comparativos en el futuro para confirmar y expandir estos hallazgos.

**Cambios Electroencefalográficos en la Migraña Hemipléjica**

Hyun Suk Oh Kim^1^, Raúl Armas Zurita^1^, Cristina Moreno Jiménez^1^, Fadi Hallal Peche^1^, Mariano Aguilera Vergara^1^, Cielo Dayana González Mendoza^1^, Laura Menéndez Rúa^1^

^1^Hospital Central de la Defensa Gómez Ulla, 28047 Madrid, España

**Introducción y Objetivos**: La migraña hemipléjica (MH) es una forma rara de migraña con aura que se caracteriza por episodios de debilidad motora transitoria, acompañada de déficits neurológicos y síntomas variados. El electroencefalograma (EEG) es una herramienta útil en el diagnóstico y seguimiento de estos pacientes, diferenciando el aura migrañosa de otras patologías. **Material y Métodos**: Se trata de un paciente de 17 años, con antecedentes de migraña que presentó visión de sombras, seguido de cefalea hemicraneal izquierda y hemiparesia derecha. Durante el traslado, se describió una posible crisis generalizada con relajación de esfínteres. Los estudios complementarios iniciales, que incluyeron analítica sanguínea, punción lumbar y TC craneal, descartaron otras patologías agudas graves. **Resultados**: Se realizaron varios registros EEG basales. El primero no mostró actividad epileptiforme y la actividad de fondo fue compatible con mediatización farmacológica. Los estudios subsiguientes revelaron consistentemente signos de afectación cortical difusa y focal con marcada asimetría interhemisférica. Esta afectación fue de grado severo predominantemente en las regiones fronto-temporales derechas, con alta persistencia de actividad lenta. En ninguno de los registros EEG se objetivó actividad epileptiforme, a pesar de la sospecha clínica inicial de crisis. Los últimos EEG mostraron datos de mejoría, incluyendo mayor reactividad en el hemisferio derecho y disminución de la actividad lenta, aunque persistía afectación focal derecha. **Discusión/Conclusiones**: El EEG en este caso de migraña hemipléjica mostró actividad lenta asimétrica, compatible con disfunción cortical transitoria, además de lentificación focal fronto-temporal derecha. En este caso, los hallazgos EEG guiaron hacia otras posibles causas de bajo nivel de consciencia pese a la clínica inicial, posibilitando el uso racional de fármacos en la UVI.

**Características del Estado Ictal-Interictal Continuo en Pacientes Neurocríticos: Experiencia en Nuestro Centro**

Estuardo Daniel Castro Ruiz^1^, José Antonio Pérez Martínez^1^, Nuria Álvarez López-Herrero^1^, María José Postigo Pozo^1^, Victoria Fernández Sánchez^1^

^1^Hospital Regional Universitario de Málaga, 29010 Málaga, España

**Introducción y Objetivos**: El estado ictal-interictal continuo (IIC) es un patrón electroencefalográfico emergente, con actividad epileptiforme persistente que no cumple criterios de estatus epiléptico (EE), pero se ubica en un espectro potencialmente ictal con posible efecto deletéreo. Se observa en pacientes críticos con alteración del nivel de conciencia y representa un aspecto creciente de interés por su asociación a peor pronóstico. La incidencia estimada varía del 5 al 15%, aunque en España los datos son limitados. La complejidad diagnóstica y la variabilidad interobservador lo hacen un tema en investigación activa, con implicaciones aún por esclarecer. **Material y Métodos**: Estudio descriptivo de pacientes diagnosticados de patrón IIC en estudios EEGs desde enero 2024-marzo 2025. Se recogieron variables demográficas, clínicas, antecedentes neurológicos, etiología primaria y el tratamiento recibido. Se analizaron características electroencefalográficas según la nomenclatura de la ACNS: actividad de base, frecuencia, localización y distribución de las descargas periódicas (PDs), modificadores plus y presencia de SIPDs. **Resultados**: Se incluyeron 43 pacientes (60,5% mujeres), edad media: 69 años. Se realizó una media de 5 EEGs por paciente, mediana de seguimiento con EEG de 9 días. Las principales etiologías fueron lesión cerebral aguda estructural (39%) y encefalopatía infecciosa/metabólica (23%). Los hallazgos EEG más frecuentes fueron LPDs (72%), frecuencia 1–2 Hz en el 53%. El 46% presentó modificadores plus y 16% SIPDs. El 28% tuvo crisis epilépticas previas, y 16% evolucionaron a EE tras el diagnóstico de IIC. Se usaron LCM (84%), LEV (81%) y VPA (30%). El patrón se resolvió en 51%, con 6 pacientes con reincidencia. La mortalidad global fue del 46,5%. **Discusión/Conclusiones**: El IIC se asocia principalmente a lesión cerebral aguda y presenta una evolución tórpida pese a tratamiento intensivo. La ausencia de EEG contínuo limita la evaluación. Se requieren estudios con mayor muestra y seguimiento continuo para esclarecer su significado clínico.

**Epilepsia y Ataxia Genética Asociadas a Mutación del gen *CACNA1A*: Experiencia de dos Casos y Revisión de la Literatura**

Erika Herráez Sánchez^1^, Javier Martínez Poles^1^, Rolando Agudo Herrera^1^, Miguel Pintor Zamora^1^

^1^Hospital Universitario Rey Juan Carlos, 28933 Madrid, España

**Introducción y Objetivos**: El gen *CACNA1A* codifica canales de calcio voltaje-dependientes implicados en la neurotransmisión. Variantes patogénicas en este gen se asocian a diversos fenotipos neurológicos, como la ataxia episódica, la ataxia espinocerebelosa y la migraña hemipléjica familiar. Se han reportado además distintas formas de epilepsia asociadas a este gen, como estatus epiléptico (generalmente asociado a ganancia de función del canal), crisis de ausencia (generalmente asociado a pérdida de función), o encefalopatía epiléptica, entre otras. No obstante, la literatura es escasa y heterogénea. **Material y Métodos**: Reporte de dos casos clínicos y revisión bibliográfica. **Resultados**: Se trata de dos mujeres, de 25 y 17 años, con crisis epilépticas de inicio en la infancia, diagnosticadas de epilepsia tipo ausencias infantiles, que resultan farmacorresistentes en su evolución. Ambas presentan posteriormente episodios de inestabilidad de la marcha y sensación de mareo que dificultan su actividad diaria, con mejoría parcial con acetazolamida. Los estudios v-EEG realizados mostraron ausencias típicas, actividad punta-onda generalizada intercrítica, y otras anomalías irregulares inespecíficas. La neuroimagen fue normal en el primer caso, y mostró hallazgos de Chiari tipo I en el segundo. En el estudio genético, la primera paciente presenta delección de los exones del 39 al 47 en el gen *CACNA1A*; la segunda paciente presenta una mutación puntual G>A en el exón 12 (mutación missense), ambos hallazgos descritos como causantes de pérdida de función del canal. **Discusión/Conclusiones**: La epilepsia asociada a mutaciones en el gen *CACNA1A* puede presentar fenotipos electroclínicos variados, cursa generalmente con crisis farmacorresistentes, y puede asociar otros síntomas neurológicos como disfunción cerebelosa y afectación del neurodesarrollo. Es importante conocer los distintos fenotipos y su correlación genotípica, ya que pueden ayudar a un mejor asesoramiento y manejo terapéutico en estos pacientes.

**Evolución Electroclínica de Pacientes con Epilepsia Autoinmune Asociada a Anticuerpos anti-GAD65+**

Ismael Oussama^1^, José Antonio Pérez Martínez^1^, Nadia Bocero Hanan^1^, María José Postigo Pozo^1^, Victoria Eugenia Fernández Sánchez^1^

^1^Hospital Regional Universitario de Málaga, 29010 Málaga, España

**Introducción y Objetivos**: Las epilepsias autoinmunes asociadas a anticuerpos anti-GAD65 (E. GAD65) se caracterizan por refractariedad a fármacos anticrisis convencionales y asociación frecuente con otras enfermedades autoinmunes. Analizamos una serie de pacientes con E. GAD65 para evaluar sus características electroclínicas y evolución. **Material y Métodos**: Estudio descriptivo retrospectivo de 13 pacientes (84,6% mujeres), atendidos en el Hospital Regional Universitario de Málaga con electroencefalogramas (EEG) realizados entre 2009 y 2024 y diagnóstico confirmado de enfermedad asociada a anti-GAD65 (títulos ≥10 UI/mL). **Resultados**: Edad media: 45,8 años (15–74). En 11 pacientes (80%) el motivo inicial fue sospecha de crisis epilépticas, uno presentaba trastornos del movimiento y otro crisis epilépticas vs. psicógenas. En todos los casos se estableció un diagnóstico de epilepsia focal tras realizar al menos un EEG (media: 4,1 estudios por paciente, incluyendo vídeo-EEG y polisomnogramas). 7 pacientes (53,8%) mostraron lentificación de base en algún registro. Solo un paciente presentó EEGs normales (7,7%). Se observaron paroxismos en regiones temporales derechas en 6 casos (46,2%), izquierdas en 4 (30,8%) y bilaterales independientes en 2 (15,4%). En 4 de los 11 con sospecha epiléptica inicial se consideró encefalitis activa (33,3%) y en uno se diagnosticó estatus epiléptico refractario de nueva aparición (NORSE). Los tratamientos más empleados fueron Cenobamato+Clobazam y Eslicarbazepina combinada con Brivaracetam o Perampanel. En cuanto a la evolución, 9 de los 13 (69,2%) presentaron una reducción en la frecuencia de crisis. En 10 pacientes se realizaron EEG seriados; 3 mostraron mejoría (normalización de la actividad de base/desaparición de actividad paroxística; 30%). **Discusión/Conclusiones**: Las epilepsias asociadas a GAD65 presentan un curso variable y potencialmente grave. La detección precoz y la realización de EEG seriados son clave en su abordaje.

**EEG en el Diagnóstico y Pronóstico de la Enfermedad de Creutzfeldt-Jakob**

Marta San Millán Huang^1^, Thatiana Rossi Meza Gaspar^1^, Nicole Fontana García^1^, Antonio Jesús Pedrera Mazarro^1^, Isabel Sáez Landete^1^, Guillermo Martín Palomeque^1^, Ignacio Regidor Bailly-Bailliere^1^

^1^Hospital Universitario Ramón y Cajal, 28034 Madrid, España

**Introducción y Objetivos**: La enfermedad de Creutzfeldt-Jakob (ECJ) es una causa común de demencia rápidamente progresiva. Si bien el diagnóstico definitivo es anatomopatológico, los criterios diagnósticos contemplan, junto con las manifestaciones neurológicas, la presencia de biomarcadores en líquido cefalorraquídeo (LCR) así como hallazgos en la neuroimagen y el electroencefalograma (EEG). Dado su mal pronóstico, el objetivo principal es evaluar el papel del EEG en el diagnóstico de la ECJ probable. **Material y Métodos**: Se analizaron los casos clínicos de 2 mujeres de 43 y 70 años valoradas en nuestro hospital entre los años 2022 y 2025 con diagnóstico de probable ECJ. **Resultados**: En estas pacientes, el deterioro cognitivo de 2–3 meses de evolución se asoció a síntomas cerebelosos (n = 1) y labilidad emocional (n = 1). Entre el primer y el segundo EEG se observó un rápido deterioro clínico y de la actividad bioeléctrica cerebral. Inicialmente, los hallazgos electroencefalográficos sugerían una encefalopatía difusa e inespecífica. Transcurridos 5–7 días, se registraron ondas lentas en rango de frecuencia delta y periódicas a 1 Hz, de morfología trifásica, distribución generalizada y persistencia alta. Ambas pacientes fallecieron a los 7–21 días siguientes del segundo EEG. La neuroimagen mostró una atrofia cortical con hiperintensidad cortical y de los ganglios basales en secuencias de difusión y el análisis de proteína 14-3-3 en LCR resultó positivo en las muestras enviadas (n = 1). **Discusión/Conclusiones**: La presencia de ondas lentas trifásicas es un hallazgo inespecífico presente en diversas patologías, incluso en otras demencias rápidamente progresivas. No obstante, asociado al curso clínico y los hallazgos en la neuroimagen, el EEG seriado puede facilitar de forma rápida y no invasiva la orientación diagnóstica y pronóstica en pacientes con sospecha de ECJ.

**Código Crisis: Experiencia y Análisis de la Actividad EEG en el Hospital Universitario Puerta de Hierro Majadahonda**

Patricia Eliana Sainz Vecino^1^, Virginia Russu^1^, Lucas Rafael Pérez-Cuesta Llaneras^1^, Josué Montiel Salazar^1^, Ana Isabel Puente Muñoz^1^, Alberto Ignacio Pérez de Vargas Martínez^1^, Edwin Ebrat Mancilla^1^

^1^Hospital Universitario Puerta de Hierro, 28222 Madrid, España

**Introducción y Objetivos**: El estatus epiléptico (EE) constituye una emergencia neurológica tiempo-dependiente que requiere intervención inmediata. En la Comunidad de Madrid se implementó el protocolo “Código Crisis” (CC), activado ante determinados criterios clínicos para facilitar una respuesta rápida y estandarizada. El objetivo de este estudio es presentar la experiencia de nuestro centro con el CC, evaluando su rendimiento diagnóstico, tiempos de respuesta e impacto en el manejo del EE. **Material y Métodos**: Estudio observacional retrospectivo de 99 activaciones del protocolo CC entre enero de 2024 y mayo de 2025 (15:00–08:00 h). Se realizó EEG urgente en los primeros 15–45 minutos (20 a120 min de duración). El diagnóstico de EE se basó en los criterios de Salzburgo. Se analizaron variables clínicas, demográficas y neurofisiológicas para valorar el impacto terapéutico y pronóstico del CC. **Resultados**: El 98% de los pacientes presentó alteraciones EEG; el diagnóstico de EE se confirmó en el 18,2%. Los subtipos identificados fueron: EE eléctrico focal (38,9%), generalizado (27,8%), electroclínico focal (22,2%) e ictal-interictal (11,1%). En 20 pacientes se realizó prueba farmacológica, con mejoría del trazado en el 64,6%. La mortalidad fue superior en pacientes con EE (39%) frente a los que no lo presentaron (14%). El 38,9% de los casos de EE ocurrieron en pacientes sin diagnóstico previo de epilepsia. En ausencia de actividad ictal, el 59,5% mostró actividad epileptiforme inespecífica, indicando disfunción cortical subyacente y apoyando la utilidad del EEG en este contexto. **Discusión/Conclusiones**: El protocolo CC demuestra ser una herramienta diagnóstica esencial en el abordaje urgente del estatus epiléptico. La detección de patrones epileptógenos y actividad epileptiforme subclínica refuerza el valor del EEG urgente como biomarcador, y justifica su inclusión sistemática en pacientes con alta sospecha clínica, incluso ante hallazgos iniciales no concluyentes.

**Más Allá de la Punta-Onda a 2,5 Hz: Reconocimiento de Patrones Ictales Atípicos en el Estatus Epiléptico no Convulsivo**

Josué Alberto Montiel Salazar^1^, Virginia Rusuu^1^, Patricia Eliana Sainz Vecino^1^, Lucas Rafael Pérez-Cuesta Llaneras^1^, Edwin Ebrat Mancilla^1^, Marta Vaquero Martínez^1^, Luis Fernando López Pájaro^1^

^1^Hospital Universitario Puerta de Hierro Majadahonda, 28222 Madrid, España

**Introducción y Objetivos**: El estatus epiléptico no convulsivo (EENC) es una causa infradiagnosticada de alteración del nivel de consciencia. Su identificación precoz es fundamental para evitar secuelas neurológicas irreversibles. Nuestro objetivo es destacar el valor del EEG urgente como herramienta diagnóstica y pronóstica en fase aguda, y subrayar la importancia de reconocer patrones ictales menos frecuentes. **Material y Métodos**: Se presentan dos casos clínicos de pacientes con disminución del nivel de consciencia, sin hallazgos estructurales en neuroimagen. En ambos se realizó EEG urgente ante sospecha de EENC. Se describen hallazgos EEG, respuesta a prueba farmacológica y evolución clínica. **Resultados**: El primer caso corresponde a una mujer de 22 años con afasia mixta de inicio agudo. El EEG urgente mostró actividad delta rítmica de 0,5–1 Hz, con componente agudo asociado, en el hemisferio izquierdo. El segundo caso es una mujer de 48 años con alteración del nivel de consciencia y mioclonías sutiles en regiones frontales y orofaciales. El EEG reveló crisis electroclínicas con actividad rápida en rango beta en ambas regiones frontales. En ambos casos se observaron modificaciones del trazado tras la administración intravenosa de benzodiacepinas y mejoría clínica. El EEG fue clave para el diagnóstico de estos patrones basándonos en los criterios de Salzburgo. **Discusión/Conclusiones**: El EENC es una emergencia neurológica con alta morbimortalidad que debe ser identificada y tratada de forma urgente, supone un reto diagnóstico debido a la presencia de manifestaciones clínicas sutiles y a la dificultad en la interpretación de los hallazgos EEG. El EEG en fase aguda es crucial para su diagnóstico. Es fundamental conocer e identificar patrones ictales alternativos a la clásica punta-onda a 2,5 Hz, para no retrasar el diagnóstico ni el inicio del tratamiento.

**Crisis Epilépticas Secundarias a Cefalosporinas de 5º Generación**

Marta San Millán Huang^1^, Valeria Vanessa Gallo Rivero^1^, Antonio Jesús Pedrera Mazarro^1^, Guillermo Martín Palomeque^1^, Ignacio Regidor Bailly-Bailliere^1^

^1^Hospital Universitario Ramón y Cajal, 28034 Madrid, España

**Introducción y Objetivos**: Las cefalosporinas, como ceftriaxona, cefepime o ceftazidima, constituyen una causa farmacológica de crisis epilépticas y estatus epiléptico, disminuyendo el umbral convulsivo especialmente en pacientes con insuficiencia renal. El objetivo principal es describir la aparición de crisis epilépticas por la toxicidad de las cefalosporinas de 5º generación. **Material y Métodos**: Se analizaron los casos clínicos de 2 mujeres de 70 y 87 años con enfermedad renal crónica avanzada valoradas por crisis epilépticas al 5º día de tratamiento con ceftobiprol frente a una infección respiratoria complicada tras el fracaso de varias líneas de antibioterapia en nuestro hospital en el año 2024. **Resultados**: La primera paciente presentó un estatus epiléptico por agrupación de crisis focales secundariamente generalizadas que se asociaban en el electroencefalograma (EEG) con paroxismos de punta-onda sobre áreas posteriores derechas con tendencia a la generalización. En cambio, la segunda paciente comenzó con mioclonías generalizadas de predominio axial que se relacionaban con descargas de punta-onda generalizadas de persistencia alta en el EEG. En ambas pacientes, las manifestaciones neurológicas se resolvieron con el cambio de antibioterapia y el inicio de medicación anticrisis, lacosamida en un caso y ácido valproico junto con clonazepam en el otro caso, respectivamente. **Discusión/Conclusiones**: La experiencia disponible sobre las crisis epilépticas con el uso de ceftobiprol es limitada, en sujetos con y sin trastornos epilépticos previos, aunque existe evidencia sobre la necesidad de ajuste de dosis en sujetos con insuficiencia renal. La mejoría de la clínica neurológica tras la retirada del antibiótico desencadenante y la introducción de medicación anticrisis sugiere la posible neurotoxicidad por cefalosporinas de 5º generación como el ceftobiprol en pacientes susceptibles.

**Después de la Crisis, el Conflicto: La Agresividad Postictal en Epilepsia Temporal. A Propósito de un Caso Clínico**

Sara Muniesa Lacas^1^, Christian Hernández Aranda^1^, Gonzalo Ferrer Ugidos^1^, África Bueno García^1^, Margely Abete Rivas^1^, María Martín Carretero^1^, José Miguel Léon Alonso-Cortés^1^

^1^Hospital Clínico Universitario Valladolid, 47003 Valladolid, España

**Introducción y Objetivos**: La epilepsia del lóbulo temporal (ELT) es la forma de epilepsia más frecuente en adultos, con sintomatología ictal bien caracterizada. Sin embargo, las alteraciones conductuales del periodo postictal, como la agresividad, están poco estudiadas a pesar de su relevancia clínica. Se presenta un caso de ELT farmacorresistente con episodios de agresividad tras las crisis. Describir y analizar las características clínicas, contextuales y neurofisiológicas de la agresividad postictal en pacientes con ELT, a partir del análisis de un caso clínico y datos obtenidos mediante monitorización video-EEG prolongada. **Material y Métodos**: Estudio descriptivo retrospectivo basado en un caso clínico. Se recopilaron datos de historia clínica, exploración neurológica, analíticas, estudios de imagen y video-EEG. Se utilizó también revisión bibliográfica para contextualizar los hallazgos. **Resultados**: Una paciente de 63 años con retraso moderado del neurodesarrollo y ELT diagnosticada a los 21 años, desarrolló en 2020 episodios de agresividad postcrisis. Durante su ingreso, se identificaron crisis clínicas y subclínicas de origen bitemporal. La agresividad seguía a las crisis más prolongadas, lo que sugiere una recuperación cortical incompleta y disfunción transitoria de los circuitos frontotemporales y límbicos. **Discusión/Conclusiones**: La agresividad postictal, aunque infrecuente, es una manifestación significativa. Se relaciona con la afectación de estructuras límbicas (amígdala, hipocampo) y crisis con generalización bilateral. Desde el punto de vista neurofisiológico, se asocia con hipoactividad frontal postcrisis y alteraciones funcionales en las redes límbicofrontales, responsables del control emocional e impulsividad. La agresividad postcrítica en ELT debe ser reconocida como una manifestación transitoria pero clínicamente relevante. Su estudio puede aportar claves para el abordaje terapéutico, la prevención y la comprensión de los mecanismos cerebrales que vinculan epilepsia y conducta.

**Limitaciones del Valor de la CSA en el Diagnóstico del STC Desde la Tercera Edad**

Pablo González Uriel^1^

^1^HM La Esperanza, HM Hospitales, Santiago de Compostela, 15705 Santiago de Compostela, A Coruña, España

**Introducción y Objetivos**: El síndrome del túnel carpiano (STC) es más frecuente en mujeres entre la quinta y la sexta década de la vida, pero su presencia en la tercera o cuarta edad no es infrecuente, sobre todo por el incremento de artrosis. La electroneurografía (ENG) es la prueba de referencia en el diagnóstico, mientras que el uso del valor del área transversal del nervio mediano (*cross-sectional area* – CSA) en la ecografía (US) ha aumentado, aunque con limitaciones en este grupo de edad. Nuestro objetivo es conocer el valor medio de la CSA en el STC a partir de los 65 años en cada grupo de gravedad, y compararlo con otros grupos de edad. **Material y Métodos**: Estudio descriptivo, serie de casos, de pacientes con sospecha de STC que se someten a ENG y US. Exploración ENG: mediano sensitivas antidrómicas a 2º, 3º y 4º dedo; motora distal del mediano. US: CSA del nervio mediano en la entrada proximal del túnel del carpo. Criterios de inclusión: pacientes de 65 a 85 años con sospecha de STC. Criterios de exclusión: traumatismos o cirugías previas de STC, carpo, mano o antebrazo; enfermedades neuromusculares de base; artritis reumatoide. Estudio estadístico realizado con el programa SPSS 29.0. **Resultados**: Se estudiaron 191 carpos de 163 pacientes (72,55 ± 5,69 años); 123 de mujeres y 68 de hombres. Según la ENG 12 no tenían STC; 18 fueron incipientes, 72 leves, 34 moderados, 48 graves y 7 muy graves. La media de la CSA fue de 8,07 mm^2^ en los no STC; 9,55 mm^2^ en los incipientes; 10,89 mm^2^ en los leves; 14,21 mm^2^ en los STC moderados; 14,69 mm^2^ en los graves y 18,39 mm^2^ en los muy graves. Según el test de Tukey HSD, las diferencias de medias entre controles e incipientes, incipientes y leves, y moderados y graves no fueron significativas (*p* > 0,05); mientras que el resto de diferencias de medias intragrupos sí lo fueron. **Discusión/Conclusiones**: Nuestros hallazgos sugieren, de acuerdo con la mayoría de autores, que la CSA, por sí sola, tiene limitaciones para clasificar la gravedad del STC a partir de los 65 años.

**Diagnóstico Diferencial de Alteraciones Motoras con Respuestas Sensitivas Conservadas: Valor de la Electromiografía**

Alexandra Margarita Navarrete Loza^1^, María Pacífica Vidal Lijo^1^, Ana Isabel Martín Vigo^1^, Adrián Baz López^1^, José Luis Relova Quinteiro^1^

^1^Hospital Clínico Universitario de Santiago de Compostela, 15706 Santiago de Compostela, A Coruña, España

**Introducción y Objetivos**: Analizar el valor diagnóstico de la ENMG en alteraciones motoras, con cuadros clínicos similares. **Material y Métodos**: Sobre una serie de pacientes se realizaron bilateralmente ENG y EMG según la clínica y hallazgos durante la exploración. Se presentan 4 casos. El caso 1 es una mujer de 35 años, exposición viral (mano-pie-boca), con fiebre, cervicalgia, debilidad progresiva ascendente de piernas; evoluciona a tetraparesia, arreflexia global, apertura ocular espontánea, con soporte respiratorio. Caso 2: varón 48 años, cuadro diarreico previo, presenta debilidad de piernas (afecta bipedestación) y manos, reflejos tricipitales 1+, aquíleos 0. Caso 3: mujer de 81 años antecedente de atragantamiento; con debilidad progresiva de piernas, lesiones dérmicas. Caso 4: mujer de 80 años, debilidad de extremidades, hiperreflexia en MS, lleva soporte respiratorio. Técnicas: ENG sensitivo-motor, onda F,EMG. **Resultados**: ENG. Todos con respuestas sensitivas conservadas. En cuanto a las motoras, el caso 1, amplitud disminuida en hemicuerpo izquierdo y respuestas abolidas en derecho; en los casos 2 y 3, amplitudes motoras disminuidas; en el caso 4 ausentes. Velocidad de conducción motora dentro de normalidad, sin bloqueos de la conducción. EMG. Caso 1: silencio en reposo, ausencia de PUM. Caso 2: silencio reposo, patrón intermedio rico proximal, sin PUM distal. Caso 3: denervación activa (descargas alta frecuencia) y trazados miopáticos. Caso 4 denervación activa incluido nivel paravertebral y ausencia de PUM. La neuroimagen del caso1 muestra lesiones desmielinizantes+hemorrágicas. En el caso 3, anti-Ro+, en imagen se detecta un cáncer metastásico. **Discusión/Conclusiones**: La exploración del caso 1–2 sugiere polineuropatía axonal motora, asimétrica [1 probable Weston Hurst] o simétrica [2, S. de Guillain-Barré/AMAN]; el caso 3 proceso miopático paraneoplásico, y el 4 afectación de asta anterior [ELA]. A pesar de una presentación clínica muy similar, el estudio ENMG muestra su valor en relación al diagnóstico diferencial de esta serie de casos.

**Diplejía Facial Como Forma de Presentación del Síndrome de Guillain-Barré: A Propósito de dos Casos**

Alicia Navarro Vicente^1^, Cristina Llorente Rubio^1^, María de los Ángeles Velázquez Gómez^1^, Augusto Duperré^1^, Miguel Herguijuela Paredes^1^, José Luis Fernández Torre^1^, Pedro José Orizaola Balaguer^1^

^1^Hospital Universitario Marqués de Valdecilla, 39010 Santander, España

**Introducción y Objetivos**: La diplejía facial aislada es una variante regional del Síndrome de Guillain-Barré (SGB), asociada con frecuencia a una infección por Citomegalovirus (CMV). La sospecha clínica y el diagnóstico neurofisiológico son fundamentales para su detección precoz. **Material y Métodos**: Presentamos dos pacientes, de 41 y 54 años, con parálisis facial periférica bilateral. Se realizó estudio neurofisiológico diagnóstico y de control, incluyendo electroneurografía (ENG) de nervio facial y de extremidades, así como estudio del reflejo de parpadeo, respuestas tardías (ondas F y reflejo H) y electromiografía (EMG) de musculatura facial y de extremidades. Asimismo, se realizaron estudios serológicos, análisis de líquido cefalorraquídeo (LCR), determinación de anticuerpos antigangliósido y resonancia magnética cerebral (RMN). **Resultados**: Caso 1. El estudio inicial (día 6) mostró un aumento de latencia y amplitud disminuida del PAMC del nervio facial, con ausencia de respuesta en reflejo de parpadeo y reflejo H. En el estudio de control (día 22), una prolongación de la latencia motora del nervio facial. La ENG de nervios de extremidades y la EMG fueron normales. Caso 2. El primer estudio (día 7) evidenció latencia aumentada y amplitud levemente disminuida del PAMC del nervio facial y un aumento de latencia de los componentes del reflejo de parpadeo. En el estudio de control (día 21), se añadió una prolongación de latencias distales en nervios de extremidades. EMG normal. En ambos pacientes se evidenció disociación albuminocitológica en LCR, serología positiva para CMV (IgM e IgG) y realce de nervios faciales en los segmentos intracanalicular, laberíntico y timpánico en la RMN. En conjunto, estos hallazgos son compatibles con una diplejía facial como forma de presentación de SGB. **Discusión/Conclusiones**: La diplejía facial puede presentarse como una variante atípica de SGB, representando un desafío diagnóstico. La sospecha clínica y una evaluación neurofisiológica exhaustiva son esenciales para un diagnóstico precoz y manejo adecuado del síndrome.

**Hallazgos Neurofisiológicos en el Síndrome de POEMS. Reporte de dos Casos**

Edwin Pérez Carro^1^, Octavio Rodríguez Gómez^1^, Catia Mª. Martínez Barjas^1^, Adriana Carballo Rey^2^, Íñigo Gredilla Zubiria^1^, Daniel García García^1^

^1^Complejo Universitario A Coruña, 15006 A Coruña, España

^2^Complejo Hospitalario A Coruña, 15006 A Coruña, España

**Introducción y Objetivos**: El síndrome POEMS es un raro trastorno paraneoplásico asociado a discrasias de células plasmáticas. La neuropatía periférica está presente en el 100% de los casos, considerándose un criterio obligatorio. Su diagnóstico requiere un alto índice de sospecha, ya que comparte características neurofisiológicas con la CIDP. Objetivo: Subrayar la importancia del estudio neurofisiológico para el diagnóstico de POEMS y presentar nuestra experiencia con dos casos recientes. **Material y Métodos**: Presentamos dos casos diagnosticados en el Complejo Hospitalario Universitario de A Coruña. Caso A: Varón de 77 años con cuadro de un año de evolución de síndrome constitucional y síntomas sugestivos de polineuropatía en miembros inferiores; se objetiva gammapatía monoclonal IgA lambda. Caso B: Varón de 80 años con un cuadro de 18 meses de evolución de síndrome constitucional y síntomas sugestivos de polineuropatía en miembros inferiores. Se objetiva gammapatía monoclonal IgA kappa. Ambos cumplen además criterios mayores y menores para diagnóstico de POEMS. En ambos casos se realiza neurografía incluyendo respuestas motoras, sensitivas antidrómicas y ondas F en las cuatro extremidades. En el paciente B se realiza ademas electromiografía en extremidades derechas. **Resultados**: En los dos pacientes se objetivó una polineuropatía de naturaleza mixta, con un patrón desmielinizante en miembros superiores (incremento en latencias distales motoras con amplitudes dentro de limites normales, marcada disminución de VC, prolongación de ondas F) y severa pérdida axonal en miembros inferiores (respuestas motoras y sensitivas ausentes o de muy baja amplitud y marcada denervación activa en la EMG en el caso B). **Discusión/Conclusiones**: Estos casos subrayan la utilidad del estudio neurofisiológico en el diagnóstico temprano del síndrome de POEMS. Dado que el POEMS comparte características neurofisiológicas con la CIDP pero requiere un manejo específico, es necesario tener presente esta entidad en el diagnóstico diferencial de las polineuropatías desmielinizantes.

**Neuropatía Tomacular, a Propósito de un Caso**

Sebastián Díaz Rodríguez^1^, Rosa Elena Sánchez Gutiérrez^1^, Mayrel Aguiar González^1^, María Ángeles Nieto Martín^1^, Mónica Cano del Pozo^1^, Pablo García Gutiérrez^1^

^1^Hospital Universitario Río Hortega, 47012 Valladolid, España

**Introducción y Objetivos**: La neuropatía tomacular es un trastorno de herencia autosómica dominante, cuyo diagnóstico se basa en la presencia de neuropatías focales recurrentes en relación con traumatismos leves o compresiones y en la historia familiar. Con las pruebas neurofisiológicas demostramos las latencias distales alargadas en estos nervios, como expresión de una polineuropatía sensitivomotora desmielinizante y la confirmación diagnóstica nos la da la presencia de la mutación PMP22. **Material y Métodos**: Caso clínico: mujer de 59 años que consulta por parestesias y dolor en mano izquierda, de meses de evolución intervenida previamente de síndrome de túnel carpiano derecho. Se realiza estudio ENG sensitivo-motor explorando los nervios: mediano, cubital, peroneal común, tibial anterior bilateral. **Resultados**: En el estudio neurofisiológico se objetivan datos ENG compatibles con polineuropatía sensitivomotora de tipo desmielinizante, evidenciando afectación de segmentos nerviosos en sitios frecuentes de compresión, presentando en el momento actual signos de STC bilateral motor y sensitivo, atrapamiento de cubital bilateral en codo, así como la compresión de ambos nervios ciáticos poplíteos externos en la cabeza de peroné. El estudio genético fue positivo Con deleción 17p11.2 que contiene el gen que codifica la proteína periférica de mielina 22 PMP22. **Discusión/Conclusiones**: Debemos sospechar una neuropatía tomacular en pacientes con neuropatías focales recurrentes, es importante ampliar estudio en los casos que objetivamos afectación de más de un nervio, ya que en muchos casos estos pacientes conviven durante años con síntomas a los que dejan de dar importancia, y de esta forma evitaríamos intervenciones innecesarias. Es importante ampliar estudio a los familiares debido a su carácter hereditario.

**Utilidad de la Electromiografía en el Diagnóstico de Sarcoma de Tejido Blando, a Propósito de un Caso**

Mayrel Aguiar González^1^, Rosa Elena Sánchez Gutiérrez^1^, Sebastián Díaz Rodríguez^1^, María Ángeles Nieto Martín^1^, Mónica Cano del Pozo^1^, Pablo García Gutiérrez^1^

^1^Hospital Universitario Río Hortega, 47012 Valladolid, España

**Introducción y Objetivos**: Los sarcomas de tejidos blandos son tumores que se pueden desarrollar en el plexo braquial de forma primaria, procedentes del plexo o secundarios procedentes de otras partes del cuerpo que se han extendido al plexo. Un importante número de casos cursan con dolor, parestesias, déficit motor y en ocasiones masas palpables. **Material y Métodos**: Caso clínico: Mujer de 36 años, fumadora moderada, sin otros antecedentes personales de interés, ni tratamientos crónicos, que consulta por parestesias, dolor y déficit motor en la mano derecha de varios meses de evolución, por lo que tras ser valorada por traumatología y objetivar un déficit de separación del quinto dedo piden una EMG, para valoración de mediano y cubital. Se realiza estudio ENG motor de mediano, cubital y radial, así como sensitivas de mediano, cubital, cubital dorsal, cutáneo antebraquial medial derechos y EMG de músculos: flexor cubital del carpo, flexor radial, extensor común de los dedos, abductor del meñique, abductor corto del pulgar y tríceps derechos. **Resultados**: Se objetivan amplitudes reducidas de mediano y cubital motor derechos, respuestas sensitivas reducidas en cubital, cubital dorsal y cutáneo antebraquial medial derechos. En la EMG se evidencia abundante denervación y perdida de unidades motoras en musculatura C8-T1, concluyendo que se objetiva una afectación del tronco inferior del plexo braquial derecho, por lo que se pide una RMN que evidencia una masa tumoral en el trayecto de la raíz T1 derecha. Se realiza biopsia donde se confirma la presencia de un tumor maligno de alto grado muy indiferenciado. **Discusión/Conclusiones**: Aunque la electromiografía en el contexto de un sarcoma no es una prueba de diagnóstico principal ya que se utiliza más para seguimiento de tratamiento y para valorar compresión o daño nervioso una vez hecho el diagnóstico, en este caso, el estudio neurofisiológico ha sido una herramienta de gran valor para el diagnóstico de un tumor maligno de la vaina nerviosa, considerado como un sarcoma de tejido blando muy agresivo.

**Síndrome de Parsonage-Turner: Revisión de Nuestra Casuística**

María Jesús Lardelli García^1^, Paloma Villalobos López^1^, Clara Adela Carrasco Méndez^1^, Irene Lucena Padrós^1^

^1^Hospital Universitario Torrecárdenas, 04009 Almería, España

**Introducción y Objetivos**: La enfermedad de Parsonage-Turner es un trastorno neuromuscular infrecuente caracterizado por dolor braquial agudo, debilidad muscular y atrofia. Su diagnóstico es clínico y neurofisiológico. El objetivo del trabajo es describir los hallazgos neurofisiológicos en una serie de 10 pacientes diagnosticados en nuestra consulta, con atención a los nervios periféricos más frecuentemente afectados. **Material y Métodos**: Se revisaron retrospectivamente los estudios de electroneuromiografía de 10 pacientes diagnosticados con Parsonage-Turner entre 2015–2025. Se recogieron datos clínicos y ENMG, estudios electroneurográficos (ENG), sensitiva y motora, con especial énfasis en los nervios afectados, así como electromiografía (EMG), de la musculatura dependiente. El diagnóstico se basó en la presentación clínica, la ENMG, distribución nerviosa compatible y pruebas de imagen. **Resultados**: Los nervios más frecuentemente afectados en nuestra serie fueron el musculocutáneo (5 pacientes), seguido del axilar (4), torácico largo (3), supraescapular (3), frénico (2) y espinal (1). En siete de los diez casos se observó afectación multifocal. El inicio de los síntomas fue agudo en todos los pacientes, con evolución a debilidad motora en 5–7 días. El estudio ENMG confirmó la localización y extensión de la lesión, así como descartó otras etiologías. **Discusión/Conclusiones**: Nuestros hallazgos coinciden con la literatura, que describe una afectación frecuente del nervio musculocutáneo y una presentación variable del resto de los nervios del plexo braquial. La afectación del nervio frénico, aunque menos común, es relevante por su implicación respiratoria. La variedad de nervios afectados refuerza la etiología inflamatoria o autoinmune focal del plexo braquial. La ENMG es clave en el diagnóstico, permitiendo identificar los nervios comprometidos y orientar el manejo clínico. En nuestra serie, el nervio musculocutáneo fue el más afectado, seguido del axilar y el torácico largo. La heterogeneidad en la distribución nerviosa apoya su carácter multifocal.

**Síndrome de Parsonage-Turner. Una Serie de Casos**

Lledó Orenga Pachés^1^, Silvia Parra Escorihuela^1^, José Vicente Orenga Orenga^1^, Alina Denisa Ghinea Nuta^1^, Claudia Collado Andrés^1^, Nuria Ruiz Montagud^1^, María Calatayud García^1^

^1^Hospital General Universitari de Castelló, 12004 Castellón de la Plana, Comunidad Valenciana, España

**Introducción y Objetivos**: El síndrome de Parsonage-Turner (SPT) o neuralgia amiotrófica, es una neuropatía de uno o más nervios, con presentación clínica típica unilateral y monofásica de dolor, debilidad e hipoestesia en la extremidad superior con afectación principal del tronco superior, que suele ocurrir tras un antecedente estresante. También existen casos atípicos sin dolor, en extremidad inferior, bilaterales y recurrentes. Se trata de un diagnóstico de exclusión. El objetivo es describir los casos estudiados en nuestro servicio compatibles con SPT. **Material y Métodos**: Estudio descriptivo retrospectivo de los pacientes estudiados en nuestro servicio entre 2016 y 2024, revisando los casos compatibles con SPT y describiendo sus características clínicas y del estudio neurofisiológico. **Resultados**: Se identificaron 17 estudios, que correspondieron a 15 pacientes, 14 hombres y 1 mujer. Edad media 64,7 años. Presentación clínica: dolor 73,3% (11/15), atrofia 73,3%, clínica sensitiva 60% (9/15). Presentaron antecedentes clínicos relacionados 4 pacientes. Resultados del estudio neurofisiológico: conducciones sensitivas alteradas en 46,7% (7/15) de los pacientes y conducciones motoras alteradas en el 73,3% (11/15); estudio de aguja: signos denervativos y reinervativos en todos los casos según tiempo de evolución. Localización lesiva: 9 plexo, 5 plexo/raíz, 1 nervio periférico; de los 11 pacientes con afectación del plexo braquial, 54,5% (6/11) tenían afectación del tronco superior, 27,3% (3/11) del tronco superior y medio y 18,2% (2/11) del medio e inferior. Lesión de plexo lumbosacro en el 26,7% (4/15). Afectación miembros superiores 9 unilateral y 2 bilateral; miembros inferiores 3 unilateral y 1 bilateral. **Discusión/Conclusiones**: En nuestra serie la presentación más frecuente fue de dolor, atrofia y clínica sensitiva, unilateral, afectación de tronco superior de plexo braquial en hombres de mediana edad. Es importante reconocer las variantes atípicas, tanto clínicas como en hallazgos del estudio neurofisiológico, para realizar un correcto diagnóstico.

**Variante Inusual de la Enfermedad de Charcot-Marie-Tooth**

Clara Adela Carrasco Méndez^1^, Irene Lucena Padrós^1^, María Jesús Lardelli García^1^, Paloma Villalobos López^1^

^1^Hospital Universitario Torrecárdenas, 04009 Almería, España

**Introducción y Objetivos**: La enfermedad de Charcot-Marie-Tooth tipo 2K (CMT2K) es una forma poco frecuente de neuropatía periférica hereditaria, causada por mutaciones en el gen *GDAP1*, que alteran la función mitocondrial y la dinámica axonal. Puede heredarse tanto de forma autosómica dominante como recesiva, con una edad de inicio y expresión clínica variables. El objetivo de esta comunicación es presentar dos casos clínicos de CMT2K en pacientes de una misma familia y destacar el papel del estudio electromiográfico (EMG) en la orientación diagnóstica. **Material y Métodos**: Se describen dos pacientes de sexo masculino, primos hermanos, con clínica de debilidad muscular progresiva, alteración de la marcha y caídas frecuentes de inicio en la infancia tardía. En la exploración física ambos pacientes presentaban atrofia muscular distal en miembros inferiores con reflejos osteotendinososo abolidos. Marcha en estepaje. Dificultad para ponerse de puntillas y talones. **Resultados**: Se realizaron electroneurografías (ENG) sensitivas y motoras, ondas F y electromiografía (EMG). El estudio ENG mostró una polineuropatía sensitivo-motora axonal, con caída de amplitud de los potenciales motores y sensitivos y velocidades de conducción normales y el EMG signos de reinervación crónica, sin actividad de denervación. Estos hallazgos descartaron formas desmielinizantes de CMT y se orientó el estudio molecular hacia la secuenciación de *GDAP1*, identificándose una variante en heterocigosis que confirmó el diagnóstico de Enfermedad de Charcot-Marie-Tooth axonal tipo 2K. **Discusión/Conclusiones**: Dada su baja prevalencia, la CMT2K se considera una enfermedad rara, por lo que puede estar infradiagnosticada o mal clasificada. En este contexto, el EMG desempeña un papel fundamental al confirmar el carácter axonal de la neuropatía, diferenciándola de otros subtipos de CMT y permitiendo orientar el estudio genético de forma dirigida. Esto no solo acorta los tiempos diagnósticos, sino que también facilita un asesoramiento genético preciso y un pronóstico para los familiares afectados.

**Neuropatía Motora Axonal Aguda en Porfiria Aguda Intermitente: Reporte de un Caso y Revisión Diagnóstica**

Andrés García Verdú^1^, Diana Peñalver Espinosa^1^, Estefanía García Luna^1^, Eugenio Barona Giménez^1^, Elena Giménez López^1^, Luis García Alonso^1^, Roberto López Bernabé^1^

^1^Hospital Universitario Reina Sofía de Murcia, 30003 Murcia, España

**Introducción y Objetivos**: La porfiria aguda intermitente (PAI) es un trastorno metabólico hereditario que se manifiesta típicamente con dolor abdominal y síntomas neuropsiquiátricos. Puede cursar con neuropatía motora axonal aguda, una presentación menos habitual pero clínicamente relevante, que representa un desafío diagnóstico por su similitud con otras neuropatías motoras. **Material y Métodos**: Se presenta el caso de un varón de 51 años con diagnóstico de PAI desde 2000. En noviembre de 2024 inició debilidad progresiva en el miembro superior izquierdo, que se extendió al derecho en diciembre, estabilizándose en enero de 2025. No refería síntomas sensitivos, pero sí limitación funcional significativa en miembros superiores (MMSS), coincidiendo con episodios de dolor abdominal. Se realizó electroneurografía sensitiva y motora de MMSS y de miembros inferiores, así como electromiografía (EMG) en músculos de miotomas cervicales y lumbares bilaterales. **Resultados**: La electromiografía en febrero del 2025 mostró afectación neuropática de MMSS con disminución de las amplitudes de los potenciales motores (CMAP), con velocidades de conducción motora (VCM) conservadas, se observó actividad espontánea en los músculos de miotomos C5 y C7 izquierdas. Los datos neurofisiológicos mostraron afectación de los nervios mediano y radial bilaterales y raíces cervicales C5 y C7 izquierdas. **Discusión/Conclusiones**: El patrón clínico, con debilidad motora pura proximal y distal, cursa muy similar a la variante axonal del síndrome de Guillain-Barré, o a una multineuropatía motora. La afectación únicamente de MMSS con presencia de actividad espontánea en musculatura proximal, junto con VCM conservadas y disminución de amplitudes del CMAP en MMSS, en contexto de PAI, es compatible con una polirradiculoneuropatía motora axonal aguda asociada a brotes de PAI. En conclusión la neuropatía motora axonal aguda debe considerarse una manifestación neurológica posible en brotes agudos de PAI. La integración clínica y neurofisiológica es clave para su identificación y manejo adecuado.

**Coexistencia de Anti HMCoA y Mutación *LMNA* en Paciente Asiática con Déficit Muscular Crónico Progresivo**

Verónica Beatriz Mendoza Parra^1^, Nelson Esmir Cuéllar Ramos^2^, José Manuel Corredera^2^, Gonzalo Díaz Cano^2^, Marta Escribano Muñóz^2^, Elena Montes Fernández^2^, Mónica Salinas Rodríguez^2^

^1^Hospital Fundación Jiménez Díaz/Hospital General de Villalba, 28400 Collado Villalba, Madrid, España

^2^Hospital Fundación Jiménez Díaz, 28040 Madrid, España

**Introducción y Objetivos**: El abordaje diagnóstico de pacientes con déficit motor progresivo puede suponer un reto en casos donde la anamnesis se encuentra limitada por factores como la barrera idiomática, siendo preciso apoyarse en la exploración física y pruebas complementarias (PC) como el electromiograma (EMG). **Material y Métodos**: Presentamos el caso de una mujer de 63 años, natural de China, derivada desde Traumatología por debilidad muscular crónica en miembros inferiores (MMII), agravada en últimos dos años. Se realiza electroneurografía y EMG acorde a protocolo. **Resultados**: El EMG mostró un patrón miopático y abundante actividad espontánea en MMII, ampliando estudio a superiores, con afectación de predominio proximal, por lo que se derivó a Neurología. Las PC mostraron una CK de 575, anticuerpos anti HMGCoA positivos (negaba consumo previo de estatinas), y resonancia magnética completa con atrofia grasa en MMII, cintura pélvica y escapular. Una mutación del gen *LMNA* en el estudio genético, hizo sospechar una distrofia muscular (DM); sin embargo, la biopsia muscular con abundante necrosis sin fibrosis, fue definitiva para una miopatía necrotizante inmunomediada (MNI). **Discusión/Conclusiones**: Los anticuerpos anti-HMGCoA han sido asociados con el uso de estatinas, por lo que en adultos con episodio agudo de debilidad y este antecedente farmacológico, es fácil de sospechar; sin embargo, en pacientes con clínica de curso crónico sin dicha exposición, se entra en diagnósticos diferenciales que retrasan el inicio terapéutico. Las MNI anti-HMGCoA no asociadas a estatinas están descritas en pacientes desde edad pediátrica hasta adultez tardía, y no es rara en población China. Tienen peor pronóstico y respuesta terapéutica que los asociados a estatinas, y algunas presentan un fenotipo similar a las DM. Por otro lado, las variantes patogénicas en el gen *LMNA* dan lugar a un amplio espectro clínico que incluye la DM en cinturas. En nuestro caso, el EMG fue clave para el diagnóstico, siendo la primera exploración en orientar al origen miopático inflamatorio.

**Poliradiculoneuropatía Sensitivo Motora (Síndrome Guillain-Barré like) Inducida por Nivolumab. Estudio Evolutivo**

Mario Oswaldo Figueroa Martínez^1^

^1^Hospital Universitario Doctor Josep Trueta, 17007 Girona, España

**Introducción y Objetivos**: Nivolumab es un inhibidor de puntos de control inmunitario (IPCI) que actúa sobre la vía PD-1/PDL-1, eficaz en diversos cánceres avanzados como melanoma, cáncer de pulmón no microcítico y carcinoma de células renales. Aunque la inmunoterapia ha transformado el tratamiento oncológico, puede provocar efectos adversos inmunomediados en cualquier órgano o tejido, siendo las neuropatías inflamatorias agudas una complicación poco frecuente, pero potencialmente grave. **Material y Métodos**: Varón de 72 años con antecedentes de hipertensión arterial y diabetes mellitus controlada, diagnosticado de carcinoma papilar renal metastásico (pulmones, ganglios y psoas), en segunda línea de tratamiento con nivolumab desde enero 2024. Fue remitido por hipoestesias distales en palmas y plantas, debilidad distal en extremidades superiores e inestabilidad en la marcha, de más de un mes de evolución. Al ingreso hospitalario se realizaron estudios de conducción nerviosa, respuestas F y electromiografía de extremidades inferiores bilaterales y extremidad superior derecha. **Resultados**: Los estudios neurofisiológicos muestran (velocidades de conducción enlentecidas, potenciales disgregados con amplitud disminuida y abundantes signos de actividad espontánea en reposo) una poliradiculoneuropatía sensitivo motora simétrica, mixta, con signos de degeneración axonal aguda en músculos distales de extremidades superiores e inferiores. Se realizarán dos estudios evolutivos: uno al mes de suspender nivolumab y otro a los cinco meses, tras completar el tratamiento corticoide. **Discusión/Conclusiones**: El enfoque neurofisiológico busca identificar diferencias entre neuropatías tóxicas, inmunomediadas y neuropatías paraneoplásicas, integrando los datos clínicos con los hallazgos neurofisiológicos. La electromiografía permite confirmar el diagnóstico y caracterizar el tipo de neuropatía, pero además, el estudio evolutivo será útil como guía en el tratamiento, para diferenciar el daño agudo del crónico, y ser apoyo en decisiones complejas como la reintroducción del IPCI.

**Cuando el Dolor se Convierte en Debilidad. Neuralgia Amiotrófica. Una Serie de Casos**

Alba Sánchez Tudela^1^, Pau Mahiques Ochoa^1^, Vanesa Navarro Córdoba^1^, Sara Giménez Roca^1^, Elena Francés Jiménez^1^, Lucía Soto Manzano^1^, Andrea Aliaga Díaz^1^

^1^Hospital General Universitario Dr. Balmis, 03010 Alicante, España

**Introducción y Objetivos**: La neuralgia amiotrófica (NA) se caracteriza por dolor agudo y severo, en el plexo braquial y nervios individuales de las extremidades superiores, sin traumatismo asociado, que evoluciona a parálisis y atrofia precoz. Su diagnóstico es clínico, apoyado en pruebas como la electroneurografía (ENG) y electromiografía (EMG), clave para evitar déficits permanentes. **Material y Métodos**: Describimos tres casos de NA remitidos para estudio neurobiológico. El primer caso, varón de 36 años con un episodio autolimitado de dolor agudo en el hombro derecho hace cuatro meses, seguido de debilidad y escápula alada. El segundo, mujer de 42 años con dolor interescapular irradiado al brazo derecho y debilidad de cuatro días tras un cuadro infeccioso, asociando debilidad en la hemilengua derecha. El tercero, varón de 56 años que tras un cuadro infeccioso presenta dolor agudo y debilidad en el hombro derecho y escápula alada, parálisis facial transitoria y elevación del hemidiafragma derecho. Se realizaron EMG en músculos dependientes de nervios del plexo braquial (PB) (nervios supraescapular, axilar, pectoral lateral, musculocutáneo) y no dependientes del plexo (nervios torácico largo, dorsal de la escápula, espinal y facial), ENG sensitiva de nervios mediano, cubital, cutáneo antebraquial y radial y ENG motora de nervios mediano, cubital, axilar, musculocutáneo y espinal. **Resultados**: En el primer y tercer caso, el estudio neurofisiológico mostró afectación axonal aguda del nervio torácico largo derecho, mientras que en el segundo caso mostró afectación axonal aguda en el nervio espinal y nervios pertenecientes al tronco superior y medio del plexo braquial derecho. **Discusión/Conclusiones**: La NA, generalmente desencadenada por una respuesta inmunitaria, debido a la afectación de nervios fuera del plexo braquial recientemente se denomina “mononeuropatía múltiple aguda”. Su presentación varía desde formas clásicas, con afectación del nervio torácico largo, hasta manifestaciones atípicas como la afectación del nervio frénico o pares craneales bajos.

**Síndrome de POEMS. A Propósito de un Caso**

Beatriz Rosado Peña^1^, Juan-Bosco López Sáez^1^, José Luis Puerto Alonso^1^, Antonio Ramos Guerrero^1^

^1^Hospital Universitario de Puerto Real, 11518 Cádiz, España

**Introducción y Objetivos**: El síndrome de POEMS es un trastorno paraneoplásico infrecuente, asociado a una discrasia de células plasmáticas. El término POEMS, es un acrónimo de: Polineuropatía, Organomegalia, Endocrinopatía, Proteína M monoclonal y alteraciones cutáneas. Objetivo: Presentar un caso clínico ilustrativo que pone de manifiesto la complejidad diagnóstica y evolutiva del síndrome de POEMS, así como destacar la importancia de un enfoque multidisciplinar para su detección y tratamiento oportuno. **Material y Métodos**: Paciente de 59 años, evaluada en el Servicio de Medicina Interna. Se revisaron los AP, anamnesis, exploración clínica, pruebas complementarias, evolución clínica y estudios específicos realizados durante sucesivos ingresos a lo largo de varios meses. **Resultados**: Ingreso por debilidad y ataxia en MMII. AP: HTA, hipertrigliceridemia, DM2 y poliartralgias. Fue diagnosticada de Polineuropatía sensitivo-motora severa, con características axonales y desmielinizantes. Presentó una TVP con TEP. En el proteinograma, se observó una banda monoclonal. Evolutivamente, desarrolló esclerodermia, esplenomegalia, lesiones óseas, cambios en la coloración de la piel y aumento del vello. Tras seguimiento en consulta externa de Medicina Interna, se estableció el diagnóstico de síndrome de POEMS. **Discusión/Conclusiones**: El síndrome de POEMS se caracteriza por una presentación polisintomática y de evolución crónica, junto con polineuropatía sensitivomotora progresiva como manifestación inicial predominante. A pesar de su rareza, el reconocimiento precoz del patrón clínico y de los criterios diagnósticos es crucial, ya que se trata de una condición potencialmente tratable e incluso curable. La morbimortalidad del síndrome depende fundamentalmente del grado de afectación multisistémica y de la extensión del proceso neoplásico subyacente. Este caso resalta la necesidad de considerar el diagnóstico diferencial de polineuropatías complejas, especialmente cuando se asocian hallazgos sistémicos poco habituales.

**Ondas F-Repeaters Gigantes: A Propósito de un Caso**

Cristina Llorente Rubio^1^, Alicia Navarro Vicente^1^, Augusto Duperre^1^, Andrea Valera Barrero^1^, Pedro José Orizaola Balaguer^1^, José Luis Fernández Torre^1^, María de los Ángeles Velázquez Gómez^1^

^1^Hospital Universitario Marqués de Valdecilla, 39010 Santander, España

**Introducción y Objetivos**: Las ondas F son respuestas motoras tardías que aparecen tras el potencial de acción muscular compuesto (respuesta M), secundarias a la activación antidrómica de las motoneuronas (MTN) del asta anterior. Se denominan F-repeaters (Freps) cuando presentan misma latencia y comparten morfología, y gigantes cuando son de gran amplitud. Las Freps gigantes se describen principalmente en trastornos de la MTN espinal (ELA, poliomielitis, etc.) y se relacionan en la literatura con la activación de un grupo selectivo de MTN que han sufrido procesos de denervación-reinervación y/o hiperexcitabilidad. **Material y Métodos**: Se presentan dos pacientes remitidos por diferentes motivos (caso 1 remitido por probable ELA de inicio espinal, caso 2, por sospecha de radiculopatía), en los que realizamos estudio de conducciones nerviosas periféricas y electromiografía (en el caso 1 estudio completo, en el caso 2, de extremidades inferiores). **Resultados**: En ambos casos llama la atención la obtención de unas ondas Freps gigantes en 1 nervio (caso 1, mediano izquierdo, caso 2, tibial derecho), que es posible detectar asimismo al realizar las conducciones nerviosas periféricas motoras (CNPM) de dicho nervio. Aunque no es habitual visualizar las ondas F en las CNPM, al tener las Freps gigantes mayor amplitud, es posible detectarlas. Además, como característica distintiva, esta respuesta disminuye su latencia cuanto más proximal es el estímulo, pudiendo llegar incluso a integrarse con la respuesta M, lo que apoya su origen proximal. **Discusión/Conclusiones**: Las Freps gigantes son ondas F con latencia idéntica y gran amplitud que pueden observarse al realizar las CNPM y típicamente se acercan a la respuesta M según vamos realizando estímulos más proximales. La presencia de Freps gigantes debe sugerir como primera posibilidad diagnóstica patología de MTN espinal.

**Correlación Clínica, Electromiográfica y Genética en Miopatías Distales: Serie de Siete Casos**

Alicia Navarro Vicente^1^, Cristina Llorente Rubio^1^, Alberto Galván Jurado^1^, Alba Juárez Turégano^1^, Ana Lara Pelayo Negro^1^, Pedro José Orizaola Balaguer^1^, José Luis Fernández Torre^1^

^1^Hospital Universitario Marqués de Valdecilla, 39010 Santander, España

**Introducción y Objetivos**: Las miopatías distales son enfermedades musculares primarias genéticas caracterizadas inicialmente por debilidad y atrofia progresiva de predominio distal de extremidades. Su heterogeneidad fenotípica y genotípica dificulta su diagnóstico. La electromiografía (EMG) y electroneurografía (ENG) y otras pruebas complementarias como la resonancia magnética muscular (RMm) y los estudios genéticos constituyen procedimientos clave para su caracterización. **Material y Métodos**: Presentamos los hallazgos clínicos, radiológicos, neurofisiológicos y genéticos de siete pacientes con miopatía distal hereditaria evaluados entre 2020 y 2025. Se incluyen casos de miopatía miofibrilar (MYOT n = 2, FLNC n = 1), miopatía distal de Laing (MYH7 n = 1), miopatía de Miyoshi (DYSF n = 1), neuropatía motora hereditaria con rasgos miopáticos (VWA1 n = 1) y probable miopatía recesiva (GNE n = 1). Se analizaron datos clínicos, niveles de creatinquinasa (CK), EMG/ENG, RMm, biopsia y genética molecular. **Resultados**: El 100% de los pacientes presentó debilidad distal de predominio en miembros inferiores. El 71% mostró alteraciones en la marcha. La CK estaba elevada en el 57% de los casos. La EMG reveló un patrón miopático en el 71% de los pacientes con predominio en musculatura distal, fundamentalmente en tibial anterior, peroneo largo y gastrocnemio medial. La RMm evidenció infiltración grasa distal en el 71% de los casos. La biopsia se realizó en 5 de los pacientes, con resultados variables. **Discusión/Conclusiones**: La debilidad y atrofia muscular de predominio distal supone un reto diagnóstico por el solapamiento clínico principalmente entre neuropatías longitud-dependientes y miopatías distales. Es importante el diagnóstico diferencial y precoz, apoyado en herramientas como la RMm y el estudio neurofisiológico como complemento a un estudio genético dirigido. Es necesario ampliar las capacidades de las pruebas genéticas y dilucidar las relaciones genotipo-fenotipo para mejorar la precisión diagnóstica y fundamentar las estrategias terapéuticas y el consejo genético.

**Rostros que Hablan. Neurofisiología de la Distrofia Facioescapulohumeral**

Estefanía García Luna^1^, Diana Peñalver Espinosa^1^, Eugenio Barona Giménez^1^, Francisco Granados López^1^, Andrés García Verdú^1^, Elena Giménez López^1^, Francisco Biec Alemán^1^

^1^Hospital General Universitario Reina Sofía de Murcia, 30003 Murcia, España

**Introducción y Objetivos**: La distrofia facioescapulohumeral (FSHD) es una enfermedad genética que afecta a musculatura facial y región proximal de los miembros superiores. El diagnóstico requiere evaluación clínica, estudio neurofisiológico y análisis genético. No existe tratamiento curativo pero el manejo sintomático puede mejorar la calidad de vida. **Material y Métodos**: Varón de 35 años remitido a la consulta de NFL con diagnóstico de distrofia facioescapulo-humeral hace años para descartar la presencia concomitante de PNP. Intervenido de escápula derecha. Refiere empeoramiento con marcada debilidad en musculatura facial y parte proximal de MMSS junto con hormigueo en MMII de intensidad variable. Se realizó ENG sensitiva de ambos nervios sural, peroneal superficial, y mediano y cubital unilateral. ENG motora de ambos nervios tibial posterior y peroneal, mediano y cubital unilateral. Onda F de ambos tibiales posteriores y cubital unilateral. EMG de los músculos deltoides, bíceps braquial y tibial anterior izquierdos. **Resultados**: EF: amiotrofia en musculatura escapular y humeral de ambos MMSS, más izquierda. Amiotrofias en MMII de predominio en musculatura distal (sobre todo tibial anterior). Prominencia abdominal severa por debilidad de musculatura paravertebral y abdominal. Escápula alada izquierda (no intervenida) y derecha intervenida. Debilidad facial: facial derecho severa, facial izquierdo leve. ENG normal. EMG que muestra fibrilaciones y ondas positivas en músculo bíceps braquial izquierdo, y cambios de tipo miopático en la morfología de los PUMs de los músculos explorados. Patrón de reclutamiento incrementado y precoz para niveles bajos de esfuerzo en todos los músculos explorados. **Discusión/Conclusiones**: La ENG es importante para descartar la presencia de PNP. Aunque para el diagnóstico definitivo se requiera de la correlación clínica y el estudio genético, la EMG es fundamental en la confirmación del patrón miopático, en la evaluación de la progresión y para confirmar o descartar la presencia de otros trastornos.

**Estudios Neurofisiológicos en un Caso de Adrenoleucodistrofia en el Adulto**

Marta Escribano Muñoz^1^, Nelson Esmir Cuéllar Ramos^1^, Mónica Salinas Rodríguez^1^, Gonzalo Díaz Cano^1^, Elena Montes Fernández^1^, Blanca Patricia Díaz Montoya^1^

^1^Hospital Universitario Fundación Jiménez Díaz, 28040 Madrid, España

**Introducción y Objetivos**: La adrenoleucodistrofia (ALD) es una enfermedad ligada al cromosoma X, causada por una mutación del gen de los peroxisomas ABCD1. Se caracteriza por la acumulación de ácidos grasos de cadena muy larga (VLCFA) en todos los tejidos y en mayor medida en el cerebro y la médula espinal, lo que explica la sintomatología. Cuando la ALD se presenta en la adultez la forma más frecuente es una afectación medular de progresión lenta (adrenomieloneuropatía). La aparición de síntomas en las mujeres suele ser más tardía y los más comunes son el trastorno de la marcha y las manifestaciones vesicales e intestinales. Los potenciales evocados somatosensoriales (PESS) han demostrado ser útiles para detectar signos de afectación medular en pacientes con ALD, incluso en etapas preclínicas. Otros estudios neurofisiológicos, como el electromiograma o el estudio de nervio pudendo ayudan a completar el estudio y su diagnóstico diferencial. **Material y Métodos**: Presentamos un caso de una mujer de 50 años con diagnóstico de ALD desde hace diez (mutación c.1885 G>A; p.Asp629Asn). Se le ha realizado seguimiento en nuestro servicio desde momento del diagnóstico genético, en el que se la consideró portadora asintomática de la enfermedad, hasta este año, en el que la paciente presenta síntomas de adrenomieloneuropatía. Revisamos la progresión de su enfermedad a través de las pruebas neurofisiológicas realizadas, en el contexto de los hallazgos en el resto de pruebas complementarias y de la aparición de clínica. **Resultados**: Los electroneurogramas, electromiogramas y el estudio de suelo pélvico no han mostrado afectación periférica a lo largo de estos años. Los PESS de miembros inferiores mostraron un retraso en la conducción central desde el primer estudio, cuando los síntomas eran muy leves e inespecíficos, y han presentado un empeoramiento progresivo en todos los estudios posteriores. **Discusión/Conclusiones**: El caso destaca el valor de las pruebas neurofisiológicas en el diagnóstico temprano de la ALD y en la evolución de la enfermedad.

**Neuronopatía Sensitivo-Motora de Inicio Facial: Un Abordaje Neurofisiológico**

Gabriel Jesús Planchart Gómez^1^, Belén Clavera de la Gándara^1^, Beatriz Villarrubia González^1^, Ernesto Vargas Díaz^1^, María Ángeles García González^1^, Ángel Saponaro González^1^

^1^Complejo Asistencial Universitario de León, 24080 León, Castilla y León, España

**Introducción y Objetivos**: El FOSMN (Neuronopatía sensitivo-motora de inicio facial) es un trastorno neurodegenerativo ultra-raro descrito en 2006, caracterizado por debut de parestesias faciales y progresión sensitiva-motora craneocaudal. Su prevalencia es <1/1M y hay <100 casos reportados hasta 2019. El objetivo es presentar un caso clínico que ilustre el valor de los estudios neurofisiológicos en su diagnóstico precoz. **Material y Métodos**: Varón de 61 años con dolor y parestesias en hemicara derecha desde noviembre 2023, extendiéndose bilateralmente. Se realizaron: Exploración neurológica complete; Estudios neurofisiológicos: reflejo de parpadeo, reflejo maseterino inhibitorio, reflejo H, ENG (sensitiva, motora y onda F), EMG (territorio facial, trigémino y extremidades) y PESS (MMSS y MMII); RM cerebral, LCR, serologías y genética para excluir otras causas. **Resultados**: Clinicamente el paciente presentó una evolución craneocaudal de síntomas sensitivos trigeminales → debilidad bulbar y facial. Reflejo de parpadeo: latencia R1–R2 aumentada en V1 derecho; ausencia de respuestas en otros estímulos; Reflejo maseterino inhibitorio: ausente; ENG: neuronopatía sensitiva axonal universal, conducciones motoras preservadas; Reflejo H: ausente; EMG: actividad denervativa aguda en masetero D, frontal I. y tibial ant. D; PESS: amplitud disminuida y latencias prolongadas en mediano y tibial posterior, bilateralmente; RM: disminución cualitativa del calibre de ambos trigéminos; Analíticas: sin hallazgos patológicos. **Discusión/Conclusiones**: El patrón de neuronopatía disociada (degeneración selectiva de fibras Aβ/Aδ con preservación de C) junto con los hallazgos del reflejo de parpadeo ayudan a diferenciar FOSMN de ELA bulbar y otras neuropatías . No existen tratamientos modificadores; el manejo es sintomático. Este caso destaca la necesidad de un protocolo neurofisiológico específico para un diagnóstico precoz y preciso.

**Estudio Neurofisiológico del Nervio Frénico en Niños. A Propósito de 8 Casos**

Julián Vázquez Lorenzo^1^, María Concepción Maeztu Sardiña^1^, Pilar Rosario Martínez Martínez^1^, Victor Hugo Rubio Suárez^1^, Justo Marín Noguera^1^, Reetika Baharani Baharani^2^

^1^Hospital Universitario Virgen de la Arrixaca, 30120 Murcia, España

^2^Hospital Morales Meseguer, 30008 Murcia, España

**Introducción y Objetivos**: La parálisis diafragmática (PD) por lesión del nervio frénico causa gran morbimortalidad en niños. Suele relacionarse con cirugías cardíacas o pulmonares. La fisiopatología incluye la axonotmesis y la neuroapraxia. En éste último caso, la recuperación espontánea es esperable y puede evitarse la cirugía (plicatura). **Material y Métodos**: 8 niños con sospecha de PD en HUVA 2019–2025. El estudio neurográfico se realizó con estímulo en fosa clavicular medial, registro en apófisis xifoides y referencia en séptimo espacio intercostal en línea media clavicular. Los estudios ecográficos valoraron simetría de diafragmas a través de ventana subdiafragmática. **Resultados**: Pacientes con edades comprendidas entre 34 días de vida y 11 años con sospecha de parálisis diafragmática. Antecedentes: 3 pacientes intervenidos de cirugía cardíaca, 4 de cirugía torácica y un paciente por quimioterapia. 3 presentaron afectación de nervio frénico derecho, tres de izquierdo y dos estudios fueron normales. En un paciente se observó normalización del registro neurográfico en estudio de control (neuroapraxia). En uno de los paciente solo fue posible realizar el estudio neurográfico unilateral por presencia de vía central, completándose el estudio con ecografía e infiriendo normalidad por la simetría en dicha prueba. Para el diagnóstico de lesión de nervio frénico se utilizó la asimetría en latencia y/o amplitud, dada la divergencia de los valores de normalidad. Datos de normalidad en nuestra serie: <2 años la latencia entre 2,7 y 5,2 ms y la amplitud entre 0,4 y 1,3 mV. >2 años latencia entre 3,5 y 4,1 ms y la amplitud entre 0,6 y 1,1 mV. **Discusión/Conclusiones**: El estudio neurográfico del nervio frénico es fundamental, pudiendo diferenciar entre casos de axonotmesis y de neuroapraxia si se realizan estudios seriados y evitando cirugías innecesarias. Su combinación con otras técnicas diagnósticas (ecografía), puede mejorar la precisión. Las asimetrías de latencia y/o amplitud en la neurografía son fundamentales para el diagnóstico.

**Hallazgos Neurofisiológicos de una Serie de Casos de Neurotoxicidad por Consumo Recreativo de Óxido Nitroso**

María Mercedes Gallego de la Sacristana López Serrano^1^, Yaiza Guede Guillén^1^, Aiala Sáez Ansotegui^1^, María Cortés Velarde^1^

^1^Hospital Universitario Gregorio Marañón, 28009 Madrid, España

**Introducción y Objetivos**: El óxido nitroso (N_2_O) ha ganado popularidad como droga recreativa. Describimos los hallazgos neurofisiológicos de una serie de pacientes con síntomas atribuidos a su consumo. **Material y Métodos**: Se analizaron retrospectivamente las características clínicas, electrofisiológicas y de resto de pruebas complementarias de 6 pacientes ingresados en nuestro centro por patología asociada a N_2_O en 2024–2025. **Resultados**: De los 6 pacientes analizados, 33% presentó clínica sensitiva y 66% sensitivo-motora de extremidades. 60% de estudios de conducción nerviosa realizados mostró polineuropatía (PNP) sensitivo-motora, en su mayoría de predominio motor, de tipo mixto con predominio axonal y longitud-dependiente. 4 de 5 estudios de potenciales evocados somatosensoriales realizados y 2 de 3 estudios de conducción motora central fueron patológicos. En un paciente se hizo electromiograma (EMG), que mostró denervación aguda y cambios neurógenos crónicos. Hubo disociación albúminocitológica en 2 de 4 punciones lumbares realizadas. Los niveles séricos de vitamina B12 fueron normales en el 66% y la resonancia magnética nuclear medular fue patológica en 3 de 5 realizadas. **Discusión/Conclusiones**: El consumo abusivo de N_2_O puede causar daño de sistema nervioso central y periférico en forma de PNP, degeneración combinada subaguda y encefalopatía. Su potencial efecto neurotóxico se ha relacionado con la inactivación irreversible de la vitamina B12. Los casos de PNP de nuestra serie tienen características longituddependientes, con afectación de predominio motor y tipo axonal, al igual que la mayoría de casos descritos en la literatura, presentando ciertas diferencias con la PNP asociada al déficit clásico de vitamina B12 (predominio sensitivo, mayor frecuencia de afectación de extremidades superiores). Según la literatura, la mayoría de pacientes presenta denervación aguda en EMG al diagnóstico. El estudio neurofisiológico es útil en el diagnóstico diferencial con otras PNP de presentación aguda, así como en la detección de un posible daño central.

**Evolución Neurofisiológica en Caso Pediátrico con Subtipo Oculofaríngeo de Síndrome de Guillain Barré**

María Mercedes Gallego de la Sacristana López Serrano^1^, Ana Paloma Polo Arrondo^1^, Aiala Sáez Ansotegui^1^, Rocío García Uzquiano^1^, Natalia Bravo Quelle^1^

^1^Hospital Universitario Gregorio Marañón, 28009 Madrid, España

**Introducción y Objetivos**: La polineuritis craneal (PNC) implica afectación de varios nervios craneales (NC) simultáneamente. El síndrome de Guillain Barré (SGB) es una causa rara de PNC. Presentamos la evolución neurofisiológica de un subtipo oculofaríngeo de SGB. **Material y Métodos**: Niña de 10 años sin antecedentes de interés, con cuadro de instauración aguda y rápidamente progresiva de disartria flácida, diplopía y disfagia, sin afectación de extremidades ni ataxia; y sin signos/síntomas de infección activa concomitante. Se realiza exploración clínica, 3 estudios neurofisiológicos seriados, neuroimagen, punción lumbar y estudios de autoinmunidad y microbiológicos. **Resultados**: La clínica muestra afectación bilateral de los NC V, VI VII, IX y X. El estudio neurofisiológico (NF) en la primera semana objetiva: reflejo de parpadeo con ausencia de respuesta R1 y R2 ipsi y contralateral tras estímulo en lado derecho e izquierdo, estimulación repetitiva a alta y baja frecuencia de nervio cubital derecho normal, onda F de nervio tibial posterior y mediano derechos normales, y conducciones motoras de nervios medianos tibiales posteriores y sensitiva sural y de nervio mediano normales. A las 2 semanas se objetiva mejoría del reflejo de parpadeo bilateral, con respuestas de bajo voltaje y latencias normales. El electromiograma de músculo frontalis derecho a los 2 meses muestra moderada actividad espontánea, con escasos potenciales de unidad motora polifásicos. Tras el primer estudio NF, el resto de pruebas complementarias y la buena respuesta a inmunoglobulinas, se sugiere se trate de PNC de probable origen disinmune, que en los siguientes estudios NF se constata como tipo axonal. **Discusión/Conclusiones**: La PNC como variante de SGB tiene pocos casos descritos en la literatura. La identificación de casos atípicos de SGB es importante para el tratamiento y el seguimiento de las potenciales complicaciones. Es recomendable la realización de estudios NF seriados para confirmar tipo de lesión y pronóstico.

**Hallazgos ENG/EMG de una Paciente con Enfermedad de Charcot-Marie-Tooth Autosómica Dominante Tipo 2 Asociada al gen *MME***

Nelson Esmir Cuéllar Ramos^1^, Verónica Mendoza Parra^1^, Gonzalo Díaz Cano^1^, José Manuel Corredera Rodríguez^1^, Óscar Garnés Camarena^1^, Daniel López de Mota Sánchez^1^, Swafiri Saoud Tahsin^1^

^1^Hospital Universitario Fundación Jiménez Díaz, 28040 Madrid, España

**Introducción y Objetivos**: La enfermedad de Charcot-Marie-Tooth (CMT) o neuropatía hereditaria sensitivo-motora, es una patología de origen genético, en la que se han descrito numerosas anomalías responsables de su desarrollo. Las múltiples presentaciones clínicas observadas en la práctica reflejan heterogeneidad genética subyacente. La CM2T se ha definido como una neuropatía de inicio tardío, generalmente entre la quinta y sexta década de la vida. Tradicionalmente, los trastornos con este patrón de inicio se consideraban de herencia autosómica dominante, sin embargo, en años recientes también se han descrito formas recesivas de aparición tardía. El gen *MME*, que codifica la neprilisina, ha sido implicado en formas recesivas de CMT2. Presentamos un caso con mutación heterocigota en *MME* y fenotipo ENG/EMG atípico. **Material y Métodos**: Mujer de 51 años con debilidad rápidamente progresiva de miembros inferiores, sin antecedentes familiares conocidos. Se realizaron estudios neurofisiológicos (ENG/EMG) y análisis genético mediante secuenciación masiva dirigida a genes relacionados con neuropatía hereditaria. **Resultados**: El ENG/EMG mostró un patrón axonal con una serie de alteraciones atípicas, entre las que destaca una marcada afectación de miembros inferiores en comparación con los superiores, donde la afectación fue nula o imperceptible. Otro hallazgo relevante fue una marcada afectación motora en contraste con la afectación sensitiva. El estudio genético reveló una variante genética en heterocigosis en el exón 4 del gen *MME* (NM_007289.4): c.202C > T; p.(Arg68*), clasificada como patogénica (ClinVar ID: 851617), previamente descrita en neuropatías axonales de herencia dominante. **Discusión/Conclusiones**: El caso respalda la posible implicación de variantes heterocigotas en *MME* en neuropatías axonales de inicio tardío, ampliando el espectro clínico-genético de CMT2. La disociación motora-sensitiva, la nula/imperceptible expresión en miembros superiores debe alertar sobre fenotipos atípicos en los estudios ENG/EMG. El análisis genético es clave en estos cuadros.

**Miopatía inflamatoria Asociada a Infección por HTLV-1: A Propósito de un Caso**

Nelson Esmir Cuéllar Ramos^1^, Alba María González Martínez^1^, Verónica Mendoza Parra^1^, Gonzalo Díaz Cano^1^, José Manuel Corredera Rodríguez^1^, Óscar Garnés Camarena^1^, Marta Escribano Muñoz^1^

^1^Hospital Universitario Fundación Jiménez Díaz, 28040 Madrid, España

**Introducción y Objetivos**: La miositis inflamatoria aguda es una entidad caracterizada por inflamación súbita y destrucción del músculo esquelético, generalmente secundaria a infecciones. Una causa infrecuente es la infección por HTLV-1 (virus linfotrópico de células T humanas tipo 1). Aunque la manifestación neurológica más común es la paraparesia espástica, la afectación muscular es rara y cabe destacar que también puede simular una polimiositis. Nuestro objetivo es presentar un caso de miopatía inflamatoria focal asociada a HTLV-1 con afectación predominante de músculos gemelos, ilustrando una presentación clínica atípica **Material y Métodos**: Varón de 43 años, natural de Perú, sin antecedentes personales relevantes, con cuadro progresivo de paraparesia espástica de cuatro años de evolución. Se realizaron: Resonancia magnética (RM) de musculatura de miembros inferiores. Electromiografía (EMG) y estudios neurofisiológicos. Analítica con niveles de CPK. Serología HTLV-1. **Resultados**: La RM mostró hallazgos compatibles con miopatía focal, predominantemente en músculos gemelos. El EMG evidenció patrón miopático generalizado, con signos inflamatorios agudos exclusivos en gemelos. Se detectó CPK elevada. Los PESS mostraron alteración bilateral de la conducción a nivel central. Los estudios de conducción nerviosa y estimulación repetitiva normales. **Discusión/Conclusiones**: Lo clásico en este tipo de miopatías por HTLV-1 es una afectación de musculatura proximal, aunque también se han descrito casos de miopatías axiales, con atrofia muscular paravertebral significativa. Este cuadro puede también simular una polimiositis, tanto clínica como histológicamente. Nuestro caso los hallazgos más marcados fueron paradójicamente en músculos gemelos. Esto destaca la importancia de considerar el HTLV-1 como etiología en miopatías inflamatorias, especialmente en pacientes con contexto epidemiológico compatible. El EMG es esencial para la identificación de afectación inflamatoria, la exclusión de otras causas y el seguimiento clínico.

**Análisis de Llenado EMG: Un Nuevo Método Para la Evaluación del Reclutamiento de Unidades Motoras con EMG de Aguja**

Cristina Mariscal Aguilar^1^, Silvia Recalde Villamayor^2^, Paula Villacís Palacios^1^, Javier Navallas Irujo^2^, Javier Rodríguez Falces^2^

^1^Hospital Universitario de Navarra, 31008 Pamplona, España

^2^Universidad Pública de Navarra, 31006 Pamplona, España

**Introducción y Objetivos**: La progresión del reclutamiento de los potenciales de unidad motora (PUM) durante el aumento de la contracción voluntaria puede proporcionar información importante sobre las unidades motoras (UM) que inervan un músculo. En este trabajo, describimos un método para cuantificar el nivel de reclutamiento de la señal electromiográfica intramuscular (iEMG) durante un nivel de fuerza creciente. **Material y Métodos**: Se registraron señales de EMG con aguja concéntrica del tibial anterior de sujetos sanos a medida que la fuerza se incrementaba gradualmente desde 0 hasta la fuerza máxima. El proceso de llenado del iEMG se analizó midiendo el factor de llenado EMG (FF), calculado a partir del iEMG medio rectificado y la raíz cuadrada media del iEMG. **Resultados**: (1) La actividad del iEMG a bajas fuerzas de contracción fue discreta (FF <0,3) para todos los participantes. (2) La actividad iEMG al máximo esfuerzo fue completa (FF <0,5) en el 83% de los participantes, mientras que se redujo de forma incompleta (0,3 < FF < 0,5) en el 17%. (3) La FF aumentó rápidamente para fuerzas de hasta el 20% de la CVM y luego se estabilizó para fuerzas mayores: por lo tanto, la curva de FF presentó una forma exponencial típica. **Discusión/Conclusiones**: El método de llenado iEMG puede considerarse de aplicabilidad general, ya que la FF aumentó en un amplio rango en todos los participantes sanos. El análisis de llenado EMG podría tener el potencial de detectar escenarios de pérdida y remodelación de UM en enfermedades neurogénicas y de la neurona motora.

**Perímetro Distal del Nervio Mediano Registrado por Ultrasonido Como Predictor de Sindrome de Túnel Carpiano**

Óscar Manzanilla Zapata^1^, Beatriz Echeveste González^1^, Romina Zapatel^1^, Alberto Solís Martín^1^, Manuel Alegre Esteban^1^, María del Carmen García Penco^1^, Elena Urrestarazu Bolumburu^1^

^1^Clínica Universidad de Navarra, 31008 Pamplona, España

**Introducción y Objetivos**: La ecografía neuromuscular del nervio mediano ha emergido como una herramienta diagnóstica complementaria de gran valor en el síndrome del túnel carpiano. El papel de la ecografía es fundamental en el diagnóstico integral del STC, especialmente en casos atípicos o en etapas tempranas de la enfermedad. El análisis de las principales medidas que se registran en ecografía de nervio periférico (área y perímetro) pueden revelar correlaciones con las escalas diagnósticas actuales y su valor como elemento predictor de patología. **Material y Métodos**: El presente estudio retrospectivo de la base de datos de estudios de velocidad de conducción, electromiografía y ecografía neuromuscular realizados a pacientes con sospecha de neuropatía por atrapamiento en el de túnel carpiano recopiló los hallazgos de 20 sujetos con diagnóstico final positivo para neuropatía de nervio mediano y 20 controles sin patologías. Los datos fueron evaluados con métodos estadísticos no paramétrico buscando correlación de los valores y la escala diagnóstica (correlación de Spearman) y su relación predictiva de patología con análisis de regresión linear, con niveles preestablecidos de significancia de 0,05. **Resultados**: El área y perímetro distal del nervio mediano, medidos por ecografía, fue significativamente más alto en el grupo de neuropatía de nervio mediano vs el grupo control con una correlación clara (*p* < 0,05). El análisis de regresión linear reveló que el perímetro distal del nervio mediano tiene una significancia estadística como predictor de patología por atrapamiento en el túnel carpiano similar a velocidad de conducción sensitiva y latencia motora distal (*p* < 0,05). **Discusión/Conclusiones**: Se observa una clara correlación entre los valores de área y perímetro distales del nervio mediano, medidos por ecografía y la escala diagnóstica de neuropatía por atrapamiento de nervio mediano en el túnel carpiano. El perímetro distal se evidencia como factor predictor de patología de forma similar a estudios de velocidades de conducción.

**Utilidad del Test de Ejercicio para la Evaluación Neurofisiológica de la Parálisis Periódica Hipopotasémica**

Maria Camila Bautista Villavicencio^1^, Almudena Martínez Pérez^1^, Susana Santiago Pérez^1^, Juan José Navarrete Pérez^1^, Juan Manuel Escobar Montalvo^2^

^1^Hospital Universitario La Paz, 28046 Madrid, España

^2^Hospital Universitario Henares, 28805 Madrid, España

**Introducción y Objetivos**: La PP hipopotasémica (PPH) es una metabolopatía hereditaria caracterizada por episodios de parálisis flácida desencadenados por dieta, ejercicio y/o inmovilidad. Presentamos a una mujer con episodios de debilidad y fatigabilidad en que el test de ejercicio (TE) como parte de la evaluación neurofisiológica fue fundamental para la resolución del caso. **Material y Métodos**: -. **Resultados**: Mujer de 34 años, tiene hermano con PPH genética y antecedentes de hipotiroidismo y depresión tratados. Refiere desde la infancia episodios intermitentes de debilidad muscular y fatigabilidad de predominio en miembros inferiores, con empeoramiento reciente. La marcha y balance muscular son normales, con atrofia muscular distal leve. Dada la alta sospecha diagnóstica, se indicaron: analítica sanguínea, electromiografía y estudio genético. La analítica mostró un leve incremento de creatinfosfokinasa y autoanticuerpos de miopatías inflamatorias negativos. El TE según lo descrito por McManis, registra la disminución progresiva de la amplitud del potencial de acción compuesto muscular (PAMC) tras ejercicio local (contracción muscular isométrica voluntaria), con punto de corte por disminución ≥40% en amplitud o ≥50% en área del PAMC. Esto indirectamente representa la pérdida de excitabilidad de las fibras musculares tras el ejercicio. El estudio basal de la paciente mostró conducciones nerviosas normales y electromiografía con rasgos miopáticos. El TE con estímulo de nervio cubital y registro en abductor digiti minimi indicó una disminución progresiva de la amplitud del PAMC máxima a los 45 minutos (65%). Hallazgos que, junto al antecedente familiar sugirió de forma robusta el diagnóstico de PPH, por lo que se inició tratamiento. Diagnóstico confirmado posteriormente por mutación del gen *SCN4A*. **Discusión/Conclusiones**: El TE es una herramienta relativamente sencilla con una especificidad elevada que permite aportar datos objetivos sobre la patología y facilitar el diagnóstico diferencial.

**Neuropatía anti-MAG: Cuándo Sospecharla**

María de los Ángeles Velázquez Gómez^1^, Alicia Navarro Vicente^1^, José Luis Fernández Torre^1^

^1^Hospital Universitario Marqués de Valdecilla, 39010 Santander, España

**Introducción y Objetivos**: La neuropatía por anticuerpos (Acs) contra la glicoproteína asociada a la mielina (anti-MAG) es un tipo de polineuropatía inflamatoria desmielinizante crónica (CIDP) paraproteinémica que aparece en pacientes con gammapatía monoclonal de significado incierto (MGUS) IgM. Aunque clásicamente se ha descrito como una entidad homogénea, consistente en un cuadro típico de ataxia y temblor progresivos con un patrón de desmielinización adquirida distal y simétrica (DADS), cada vez son más los artículos que subrayan la heterogeneidad de la afectación neuropática de los pacientes con anti-MAG, distinguiendo un subgrupo típico (el clásico, con DADS y títulos altos de Acs) y uno atípico (tipo CIDP, títulos menores de Acs). Es importante reconocer el patrón neurofisiológico tipo DADS del subgrupo típico ya que presentan pobre respuesta al tratamiento habitual del resto de CIDP. **Material y Métodos**: Paciente de 70 años, con MGUS IgM, remitido a nuestra consulta por ataxia, ante sospecha de polineuropatía asociada. Se realiza estudio de conducciones nerviosas periféricas sensitivo-motoras de miembros superiores e inferiores, incluyendo segmentos proximales y ondas F. **Resultados**: Se objetivan datos de afectación nerviosa de predominio desmielinizante en nervios sensitivos y motores de miembros inferiores y superiores. Llama la atención una prolongación de la latencia motora distal desproporcionada respecto a las velocidades de conducción, sobre todo en nervio mediano y tibial bilateral, con valores bajos en el índice de latencia terminal (ILT). No se objetivan bloqueos de la conducción. Ante los hallazgos descritos, se recomienda la determinación de Acs anti-MAG, resultando en valores significativos. **Discusión/Conclusiones**: Debemos reconocer el patrón tipico de neuropatía anti-MAG en aquellos pacientes con ataxia de la marcha que presenten desmielinización adquirida de predominio distal, con valores bajos de ILT (útil como marcador electrofisiológico de desmielinización distal desproporcionada).

**Asociación Entre Escalas Clínicas y Valores Neurofisiológicos en Pacientes Operados de Mielopatía Cervical Degenerativa**

Lena Verdaguer Barberan^1^, Alberto Vidal Vidal^1^, José Luis Seoane Reboredo^1^, Manuel Ramírez Valencia^1^, Núria Raguer Sanz^1^

^1^Hospital Universitari Vall Hebron, 08035 Barcelona, España

**Introducción y Objetivos**: La mielopatía cervical degenerativa (MCD) es una de las principales causas de disfunción medular progresiva en adultos. No queda claro que los estudios neurofisiológicos (NF) se correlacionen con la severidad de la mielopatía. El objetivo era estudiar si la magnitud de cambio de las escalas clínicas se asociaba a cambios NF, incluyendo la latencia de los Potenciales Evocados Somatosensoriales (PES) y el tiempo de conducción central motor (TCC) con Estimulación Magnética Transcraneal de extremidades inferiores (EEII). **Material y Métodos**: Evaluamos, retrospectivamente, 36 pacientes (edad media 59 años, 66,7% hombres) con MCD mediante escalas clínicas prequirúrgicas (preIQ), a los 6 y 12 meses tras la intervención (6PO y 12PO) y estudios NF al menos uno preIQ y uno PO. **Resultados**: Estudiamos las diferentes relaciones entre PES de EEII y TCC de EEII con diferentes escalas clínicas. En conjunto, observamos que el TCC de EEII se relaciona con más escalas que los PES de EEII, tanto en situación preIQ como PO. Además, el TCC de EEII se correlaciona con más escalas clínicas a nivel preIQ, con menos escalas a los 6PO y todavía menos a los 12PO. Caben destacar: (a) una correlación negativa moderada entre la escala de la Asociación Japonesa de Ortopedia modificada (mJOA) y PES de EEII a nivel preIQ y 12PO (r entre –0,22 y –0,42, *p* entre 0,01 y 0,2); y (b) a nivel de TCC de EEII, correlaciones positivas con el walking test de 30 metros preIQ (EID r = 0,62 y *p* > 0,001, EII r = 0,46 y *p* = 0,01), 6PO (EID r = 0,69 y *p* > 0,0001, EII r = 0,59 y *p* = 0,001) y 12PO (EID r = 0,62 y *p* > 0,001, EII r = 0,56 y *p* = 0,002). **Discusión/Conclusiones**: Pese a las limitaciones del estudio, que incluyen un bajo tamaño muestral, lesión medular heterogénea y los estudios NF realizados a diferentes tiempos, los resultados indican que los parámetros NF se relacionan con algunas escalas clínicas. El estudio sugiere que los estudios NF son más eficientes a tiempos determinados, sobre todo a nivel preIQ.

**Correlación y Variabilidad de la Expresión Clínica con la Severidad Objetiva del Síndrome del Tunel del Carpo (STC)**

Gian Claudio Dal Boni Gómez^1^, Alejandro Soriano Garcia^1^, Beatriz Lozano Aragoneses^1^, Paula Carvajal García^1^, Maruvic Ramírez Arvelo^1^, Consuelo Valles Antuña^1^

^1^Hospital Universitario Central de Asturias (HUCA), 33011 Oviedo, España

**Introducción y Objetivos**: El STC es la neuropatía por atrapamiento más común (prevalencia 35%). Su clínica es variable (80% sensitiva, 20% motora), y patologías coexistentes (artrosis, artritis) pueden complicar el diagnóstico. La clínica sola puede ser insuficiente para un diagnóstico definitivo. Comprender la relación entre esta variabilidad clínica y la severidad objetiva (ECO, ENG) es crucial para optimizar el diagnóstico y adecuado tratamiento. **Material y Métodos**: Estudio transversal retrospectivo de 42 pacientes (73 manos evaluadas) con sospecha de STC. Se analizaron variables: clínica (sensitiva, motora, sensitiva-motora), extensión (un dedo, varios dedos, toda la mano), y hallazgos superpuestos (artrosis, deformidades, etc.). Se incluyó la presencia de signos de Tinnel (TT+) y Phalen (TP+). La severidad se evaluó por impresión diagnóstica ECO y ENG. Se usó la V de Cramer para correlación de variables cualitativas. **Resultados**: Del total (N = 73 manos), la clínica sensitiva fue predominante (84%, 62/73), seguida por motora (15%, 11/73). La afectación de “todos los dedos” fue la más común (30,14%), seguida por dedos 1-2-3 (26,03%). La correlación del tinnel con la severidad ENG fue débil-moderada (Cramer’s V = 0,35); para el phalen fue débil (Cramer’s V = 0,30). Los hallazgos superpuestos más frecuentes fueron artrosis (26,03%) y tenosinovitis (17,81%). Del total de manos evaluadas con ENG, 15 (20,55%) presentaron un ENG normal a pesar de la clínica. El VPP de la clínica (para STC por ENG anormal) fue del 88,5%, y el VPN del 75,0%. **Discusión/Conclusiones**: La clínica mostro ser un buen indicador de sospecha (VPP 88,5%, VPN 50–75%), pero puede no ser suficiente para el diagnóstico definitivo por la alta variabilidad clinica y la superposicion de otras patologías. La correlación entre la expresión clínica con la severidad objetiva es de débil a moderada, hallazgos que son corcondantes con la variabilidad clínica y sugieren la importancia de un enfoque diagnóstico multimodal.

**Nuestra Experiencia con el Estudio de Neuropatía de Fibra Fina**

Andrea Sánchez Femenía^1^, Nuria Gil Galindo^1^, Rosa Chilet Chilet^1^, Alexandra Mazzillo Ricaurte^2^, Pau Giner Bayarri^1^, Marina Murillo Martínez^1^, Lydia Ruiz Navarro^1^

^1^Hospital Universitario Doctor Peset, 46017 Valencia, España

^2^Hospital Lluis Alcanyís de Xátiva, 46800 Valencia, España

**Introducción y Objetivos**: El sistema autónomo controla funciones vitales como la circulación, respiración, digestión y metabolismo. Para evaluarlo, se usan pruebas neurofisiológicas, principalmente para estudiar sistema cardiovascular y función sudomotora. La respuesta simpática cutánea (SRS) es una de las más usadas, aunque dispositivos como el Sudoscan® permiten medir la conductancia electroquímica de la piel, evaluando la inervación autónoma distal mediada por fibras C no mielínicas. **Material y Métodos**: Estudio descriptivo y retrospectivo en el que se han incluido 49 pacientes (24 hombres y 25 mujeres) con una edad comprendida entre los 33 y los 92 años, valorados desde septiembre de 2022 hasta mayo de 2025 en el servicio de Neurofisiología Clínica del Hospital Universitario Doctor Peset, derivados para estudio de sistema nervioso autónomo. Se han recopilado datos de historias clínicas, evaluándose motivo de consulta, resultados de electroneurografía/electromiografía, Sudoscan® y respuesta simpática cutánea (SRS), entre otros. El principal objetivo del estudio ha sido evaluar la correlación entre los test diagnósticos de Sudoscan® y SRS. **Resultados**: En cuanto a los resultados de Sudoscan®, 19 (38,8%) de los 49 pacientes incluidos presentaron alteración en el mismo. Por otro lado 10 (20,4%) de los 49 pacientes presentaron ausencia de respuesta simpático refleja. Al evaluar la correlación entre ambos test diagnósticos, Sudoscan® y SRS, se observó concordancia de los resultados obtenidos en ambos test en 38 (77,6%) del total de pacientes incluidos. **Discusión/Conclusiones**: El objetivo de este estudio ha sido evaluar la posibilidad de comparar la SRS con los resultados obtenidos mediante Sudoscan®, dado que estudios previos han reportado un nivel de concordancia entre ambos test mayor del 80%. La medición de la conductancia electroquímica de la piel mediante Sudoscan®, al tratarse de una técnica no invasiva, resulta de utilidad para evaluar la disfunción autonómica en las diferentes neuropatías periféricas.

**Diagnóstico Precoz del Daño Neuropático en Amiloidosis: Sural Dorsal y Período Silente Cutáneo en Portadores y Casos**

Alba González Arjonilla^1^, Nuria Álvarez López-Herrero^1^, María José Postigo Pozo^1^, Virginia Reyes Garrido^1^, Victoria Fernández Sánchez^1^

^1^Hospital Regional Universitario de Málaga, 29010 Málaga, España

**Introducción y Objetivos**: La amiloidosis hereditaria por transtirretina es una enfermedad de presentación heterogénea, progresiva y multisistémica, potencialmente mortal. Los depósitos de amiloide afectan comúnmente al sistema nervioso periférico y, en muchos casos, a las fibras finas. Las nuevas terapias pueden modificar el curso de la enfermedad; por ello, se están investigando nuevas técnicas para detectarla de forma precoz. Presentamos un estudio en el que se han registrado el periodo silente cutáneo (PSC) y el potencial de acción sensitivo del nervio sural dorsal como posibles biomarcadores. **Material y Métodos**: Revisión descriptiva prospectiva de pacientes atendidos en la consulta multidisciplinar de amiloidosis en el Hospital Regional Universitario de Málaga desde el año 2023, a los que se les ha realizado PSC y conducciones de nervio sural y sural dorsal (cálculo de índice). **Resultados**: Se han estudiado 11 pacientes portadores (6 mujeres y 5 hombres, media de 48 años) y 4 casos índice (1 mujer y 3 hombres, media de 68 años). La latencia media del PSC para los portadores fue de 84 ms; no se obtuvo PSC en 2/4 casos índice y en los otros 2, la latencia estaba aumentada (95,25 y 107,5 ms). La media de duración del PSC en los portadores y los casos índice fue de 39,10 y 37,38 ms, respectivamente. El índice sural/sural dorsal resultó normal en todos los portadores. No se halló potencial de acción sensitivo del nervio sural en 3 de los casos índice; en el restante, el índice sural/sural dorsal fue normal. **Discusión/Conclusiones**: Existen pocos estudios que hayan empleado el PSC en pacientes con amiloidosis. Sin embargo, los resultados publicados en la literatura son similares a los observados en nuestro laboratorio. Aunque el índice sural/sural dorsal fue normal en todos nuestros pacientes portadores, supone una herramienta prometedora en la detección precoz de daño neuropático en amiloidosis, según investigaciones previas.

**Alteración de la Transmisión Neuromuscular Tras el uso de Toxina Botulínica: Dos Casos con Síntomas Miasteniformes**

Valeria Gallo Rivero^1^

^1^Hospital Universitario Ramón y Cajal, 28034 Madrid, España

**Introducción y Objetivos**: El síndrome miasténico es un trastorno neuromuscular caracterizado por debilidad fluctuante y fatigabilidad muscular, causado por alteraciones en la transmisión neuromuscular. El uso prolongado de toxina botulínica (BoNT) puede, aunque raramente, desencadenar efectos adversos sistémicos, como disfunción de la unión neuromuscular. Describimos dos casos clínicos con síntomas miasteniformes tras tratamiento con BoNT. **Material y Métodos**: Caso 1: Mujer de 45 años con paraparesia espástica familiar, tratada durante dos años con BoNT tipo A (1200 U/sesión). Tras dos años de uso, comenzó con debilidad transitoria, visión borrosa y disartria. La estimulación repetitiva (ER) fue normal, pero el EMG de fibra única (EMGFU) mostró valores individuales del jitter aumentados en 8 de los 10 pares analizados, con 4 pares mostrando bloqueos. Los anticuerpos anti-AChR fueron positivos, confirmando una miastenia gravis generalizada, previamente no diagnosticada. Se considera que la BoNT pudo haber desenmascarado la enfermedad subyacente. Se inició tratamiento con piridostigmina con mejoría parcial y posteriormente se añadió azatioprina. **Resultados**: Caso 2: Varón de 52 años con paraplejia secundaria a lesión medular (C6–C7, ASIA C), tratado con BoNT tipo A (400 U/sesión) durante un año. Presentó diplopía vertical y disartria. La ER fue normal, pero el EMGFU reveló valores individuales del jitter patológicos en 6 de los 20 pares analizados, 3 de ellos presentando bloqueos. Los anticuerpos anti-AChR y anti-MuSK fueron negativos. Se inició piridostigmina (½ comp c/8 h), sin mejoría clínica; se recomendó aumentar la dosis a 1-1-1. Se considera un efecto remoto no deseado de la toxina, sin evidencia serológica de miastenia. **Discusión/Conclusiones**: Ambos casos mostraron hallazgos neurofisiológicos similares, el primero en contexto de una miastenia gravis no diagnosticada previamente y el otro sin base autoinmune confirmada. Estos casos subrayan la necesidad de realizar mas estudios en pacientes con síntomas atípicos tras el uso de BoNT.

**Utilidad de Modelos de Machine Learning Para Clasificar el Resultado Postquirúrgico del Síndrome de Túnel Carpiano Grave**

Juan Manuel Escobar Montalvo^1^, Edgar Rivera Vigil^2^

^1^Hospital Universitario del Henares - Centro de trabajo: Grupo Experto en Análisis Médico (Instituto de Tecnología), Universidad de Castilla-La Mancha (Cuenca, España), Cátedra de Inteligencia Artificial Aplicada a la Salud-Universidad de Castilla-La Mancha

^2^Hospital Universitario de Villalba, 28822 Madrid, España

**Introducción y Objetivos**: El síndrome del túnel carpiano (STC) es una neuropatía focal prevalente que impacta la salud. La cirugía es habitualmente eficaz en el STC moderado e incierta en STC grave. El objetivo fue evaluar la utilidad de modelos de machine learning (MML) para clasificar el resultado postquirúrgico (RPQx) en base a parámetros electromiográficos (EMG). Material y Métodos: Estudio de aprendizaje supervisado de clasificación realizado de datos de 111 pacientes con STC grave (ausencia de potencial sensitivo y/o latencia motora distal >7 ms y/o amplitud motora >2 mV y/o ausencia de potenciales de unidad motora en el EMG de aguja). La variable objetivo fue el RPQx (variable dicotómica): mejoría o no mejoría, definida con la escala visual analógica y el cuestionario DASH2. Tras el *data cleaning* se generaron grupos de entrenamiento (80%) y prueba (20%), se incluyeron la edad, sexo, latencia motora distal, amplitud del potencial motor, denervación y presencia de potenciales de unidad motora, y se construyeron MML de random forest (RF), Gradient Boosting (GB), regresión logística (RL) y redes neuronales (RN). La capacidad de clasificación y el rendimiento de los MML se midió con la exactitud, sensibilidad, precisión, F1 score y el área bajo la curva ROC (AUC). Los análisis se realizaron con el *toolbox Orange Data Mining.* **Resultados**: Los MML tuvieron una adecuada capacidad de clasificación con exactitud >80% (NN = 88%, RF = 87%, GB = 86% y RL = 84%), sensibilidad >90% (RN = 97%, RF = 97,5%, GB = 96% y RL = 97%), precisión >80% (NN = 90%, RF = 88%, GB = 89% y RL = 87%) y F1 score >90% (RN = 94%, RF = 93%, GB = 92% y RL = 91%). El rendimiento de los MML mostró valores de AUC >0,8 (RN = 0,79, RF = 0,69, GB = 0,68 y RL = 0,75). Esto indicaría que los MML clasifican adecuadamente el RPQx, pero con un rendimiento aceptable-bajo para NN y RL y pobre para RF y GB. **Discusión/Conclusiones**: En nuestros datos los MML empleados clasificaron adecuadamente el resultado postquirúrgico de pacientes con STC grave. Sin embargo, el rendimiento de los MML fue aceptable-bajo para RN y RL y pobre para RF y GB.

**No Es Cosa De Risa. Uso Recreativo de Óxido Nitroso y Lesión Neurológica**

Alicia Silva Catedra^1^, Patricia Orive Rodríguez^1^, Enrique Montes Latorre^1^, Francisco José Palomar Simón^1^, Rocío Vázquez Rodríguez^1^

^1^Hospital Universitario Virgen del Rocío, 41013 Sevilla, España

**Introducción y Objetivos**: El óxido nitroso (N_2_O) o gas de la risa se usa legítimamente en medicina. Sin embargo, actualmente es consumido de manera recreativa. El N_2_O inactiva la vitamina B12 mediante su oxidación interfiriendo en la acción de la metionina sintasa, que convierte la homocisteína en metionina, lo que termina afectando a la reparación y mantenimiento de la mielina. Como consecuencia del uso de este tóxico se puede producir una polineuropatía sensitivo-motora o una mielopatía subaguda combinada severa. **Material y Métodos**: Presentamos seis casos diagnosticados de alteraciones neurológicas en relación con el consumo de N_2_O. **Resultados**: Todos los pacientes acudieron a Urgencias con parestesias, debilidad y ataxia de predominio en miembros inferiores. Presentaban niveles bajos de vitamina B12 (158–270). En la mayoría de ellos la RNM fue normal. Los estudios neurofisiológicos que pudimos realizar en dos de ellos revelaron una polineuropatía sensitivo-motora axonal con afectación distal. Un dato muy significativo es que nos ha sido imposible garantizar el seguimiento de los pacientes y aumentar los estudios por las características de la población (jóvenes consumidores de tóxicos con muy escasa adherencia al seguimiento médico). **Discusión/Conclusiones**: La exposición al N_2_O de forma prolongada o tras altas dosis puede producir severas alteraciones neurológicas o hematológicas. La variabilidad depende de la reserva corporal de la vitamina B12 y de factores relacionados con su metabolismo, interfiriendo en la síntesis de ADN determinando una anemia megaloblástica o bien afectando a las vías de la metionina sintasa y de la metimalonil-CoA mutasa, generando daño neurológico. El predominio clínico de polineuropatía o mielopatía depende del grado y duración de la exposición, presencia de comorbilidad y vulnerabilidad individual. Los estudios neurofisiológicos resultaron más útiles para el diagnóstico precoz que otras pruebas. La suspensión inmediata de N_2_O y la suplementación con vit B12 son esenciales para evitar secuelas neurológicas permanentes.

**Caso Clínico de Polineuropatía anti-MAG y la Importancia de la Electroneuromiografía (ENMG), a Propósito de un Caso**

Boris Alfonso Orozco Jiménez^1^, María Asunción Martín Santidrián^1^, Olga Pérez Gil^1^, Francisco Isidro Mesas^1^, Beatriz García López^1^, Ana Isabel Gómez Menéndez^1^, Estefanía Navas Rivas^1^

^1^Hospital Universitario de Burgos, 09006 Burgos, Castilla y León, España

**Introducción y Objetivos**: Las PNP por anticuerpo anti-MAG es una enfermedad crónica y poco frecuente de origen autoinmune, que deteriora la mielina de los nervios periféricos y su causa está relacionada a la producción de IgM monoclonal que ataca la proteína MAG (glicoproteína asociada a mielina). Los individuos con PNP por MAG suelen mostrar una PNP crónica desmielinizante distal que progresa lentamente, empezando con sensaciones anormales en las extremidades que inicialmente afectan las piernas. La PNP anti-MAG puede estar inactiva durante varios años y eventualmente puede comenzar a avanzar. La identificación de la PNP antiMAG se basa en la detección de anticuerpos anti-MAG. **Material y Métodos**: Presentamos el caso de una paciente con antecedente de gamapatía monoclonal, macroglobulinemia de waldenstrom y sacoidosis, que comenzó a experimentar sensaciones anormales en las manos y los pies, junto con episodios de torpeza al caminar y fatiga. **Resultados**: En la ENMG, se observa un aumento significativo en las latencias motoras distales en todos los nervios examinados, lo que sugiere una polineuropatía desmielinizante de predominio distal asociada a un índice de latencias terminales (ILT) <0,25 en al menos 2 nervios. Esto sugiere una PNP desmielinizante vinculada a gamapatía monoclonal. Luego, mediante el uso de los laboratorios, se identifican anticuerpos anti-MAG, lo que conduce a la identificación de PNP asociada a anticuerpos antiMAG. **Discusión/Conclusiones**: La PNP anti-MAG tiene características neurofisiológicas específicas, las cuales hacen el diagnóstico muy sugestivo y se relacionan con la discapacidad funcional del paciente. El uso del (ILT) como indicador electrofisiológico para diagnóstico de PNP anti-MAG, ha sido objeto de debate. En este caso clínico afirmamos su utilidad diagnóstica y permitiendo la realización de diagnóstico diferencial con otras patologías especialmente otras formas de CIDP. La ENMG se podría utilizarse como biomarcador en la monitorización y seguimiento de los pacientes con PNP anti-MAG.

**Mioquimias, Fasciculaciones y Denervación: Diagnóstico Diferencial Entre Radiotoxicidad y Enfermedad de Motoneurona**

Patricia Orive Rodríguez^1^, Alicia Silva Cátedra^1^, Enrique Montes Latorre^1^, Mikel Salgado Irazabal^1^, Rocío Vázquez Rodríguez^1^

^1^Hospital Universitario Virgen del Rocío, 41013 Sevilla, España

**Introducción y Objetivos**: Las neuropatías inducidas por radioterapia (RT) constituyen una complicación tardía y poco frecuente en pacientes oncológicos tratados por tumores de cabeza y cuello. Su presentación clínica puede simular enfermedades de segunda motoneurona como la Esclerosis Lateral Amiotrófica (ELA), planteando un reto diagnóstico. Nuestro objetivo es describir un caso con clínica progresiva y exploraciones neurofisiológicas clave para el diagnóstico diferencial entre afectación por RT y enfermedad de motoneurona. **Material y Métodos**: Varón de 53 años con antecedentes de carcinoma escamoso cervical de primario desconocido tratado con cirugía, quimioterapia y RT bilateral cervical. Un año después inicia clínica progresiva de debilidad, atrofia y alteración sensitiva en miembro superior izquierdo, que posteriormente se lateraliza, con aparición de fasciculaciones y afectación de lengua. Se realizaron una serie de estudios ENMG en centro privado y en nuestro centro, con exploración extensa de miembros superiores, inferiores, pares craneales y nervios frénicos. **Resultados**: Los estudios ENMG mostraron denervación activa y fasciculaciones en musculatura dependiente de XI, XII, axilar y supraescapular izquierdos, con mioquimias, sin afectación difusa ni progresión a miembros inferiores ni frénicos. Las respuestas sensitivas fueron normales salvo leve atrapamiento carpiano. La neuroimagen fue no concluyente. La distribución topográfica y el patrón neurofisiológico apoyaron el diagnóstico de neuropatía por RT. **Discusión/Conclusiones**: El caso ilustra el valor de la ENMG seriada y dirigida para distinguir entre una neuropatía focal secundaria a RT y una enfermedad degenerativa difusa como la ELA. La presencia de mioquimias y la ausencia de diseminación neurofisiológica apoyaron la afectación secundaria a radiación. La correlación clínica-neurofisiológica fue decisiva para orientar el diagnóstico y el seguimiento.

**Impacto del Trasplante Simultáneo Páncreas-Riñón en la Neuropatía Periférica: Seguimiento a Cinco Años**

Dora del Pilar Sturla Carreto^1^, Karen López Viera^1^, María Victoria Alejos Herrera^1^, Raúl Sastre González^1^, Gemma Vázquez Casares^1^, Carmen Montes Gonzalo^1^, Johanna Barón Sánchez^1^

^1^Complejo Asintencial Universitario de Salamanca, 37007 Salamanca, Castilla y León, España

**Introducción y Objetivos**: La neuropatía periférica es una de las complicaciones más prevalentes en pacientes con diabetes mellitus tipo 1 (DM1). La coexistencia de insuficiencia renal (IR) contribuye a un empeoramiento de la disfunción nerviosa, traduciéndose en una neuropatía más severa. El trasplante simultáneo de páncreas y riñón (TSPR) es el tratamiento de elección para individuos con DM1 e IR terminal. Objetivo: Evaluar el impacto del TSPR en los parámetros neurofisiológicos de conducción nerviosa periférica. **Material y Métodos**: Estudio descriptivo y retrospectivo de pacientes con DM1 e IR terminal sometidos a TSPR, a los que se les realizó estudios de electroneurografía (ENG) previo al trasplante, a los 6 meses, y del 1º al 5º año post-trasplante. Se incluyeron 63 pacientes, edad media de 42 años (28–58), 70% de sexo masculino. Se registraron parámetros motores, como latencia distal (LDM), velocidad de conducción (VCM) y amplitud del potencial de acción compuesto motor en los nervios (n) peroneal común y tibial posterior bilateralmente, y en los n. mediano y cubital derechos. Se evaluó la velocidad de conducción sensitiva (VCS) y la amplitud del potencial de acción sensitivo en el n. sural bilateral, y en mediano y cubital derechos en los trayectos III y V dedo-muñeca. El análisis estadístico se realizó utilizando MATLAB (2024a). **Resultados**: Al comparar los valores pre-trasplante con los valores a 5 años, se observó un aumento significativo de las VCM y VCS en todos los nervios explorados, así como una disminución de las LDM. Sin embargo, las amplitudes de los potenciales motores y sensitivos no mostraron cambios significativos. **Discusión/Conclusiones**: Durante los primeros cinco años posteriores al TSPR se evidencia una estabilización en la evolución natural de la polineuropatía diabética, con mejoría en los parámetros de latencia y velocidad de conducción nerviosa, lo que podría indicar un proceso de reparación mielínica. La ausencia de modificaciones en las amplitudes de los potenciales sugiere una recuperación limitada del daño axonal.

**Evolución y Características Neurofisiológica en un Grupo de Pacientes con Amiloidosis por Transtiretina**

Christian Javier Hernández Aranda^1^, Sara Muniesa Lacasa^1^, Margely Abete Rivas^1^, María Martín Carretero^1^, África Bueno García^1^, Marta Ayuso Hernández^1^, José Miguel León Alonso-Cortés^1^

^1^Hospital Clínico Universitario de Valladolid, 47003 Valladolid, Castilla y León, España

**Introducción y Objetivos**: Introducción: La amiloidosis por transtiretina (*TTR*) se caracteriza por depósito extracelular de fibrillas amiloides derivadas de la proteína transtiretina. Objetivos: Analizar evolución clínica y hallazgos neurofisiológicos en pacientes portadores de mutaciones en el gen *TTR*, identificando signos precoces de afectación nerviosa y su evolución. **Material y Métodos**: Estudio descriptivo retrospectivo de una serie de casos. Material: 1. Datos clínicos de los Pacientes; studios neurofisiológicos; Referencias bibliográficas; Se revisaron 15 casos, de los cuales 4 son portadores asintomáticos y 11 sintomáticos. La selección se realizó considerando como criterio principal que los pacientes sean portadores de mutaciones en el gen *TTR*, además de haber recibido un seguimiento clínico y neurofisiológico. **Resultados**: Entre los pacientes sintomático se observó que las primeras manifestaciones fueron alteraciones en la fibra fina (A-delta), en ocasiones, asociadas a síndrome del túnel del carpo. Asimismo, se estableció que todos los pacientes con polineuropatía presentaban concomitantemente afectación de fibras finas (A-delta). **Discusión/Conclusiones**: La polineuropatía amiloidotica asociada a TTR es una enfermedad rara, neurodegenerativa, progresiva y potencialmente mortal, causada por mutaciones en el gen de la TTR que conducen a la formación de depósitos de amiloide en los nervios periféricos. La afectación inicial suele comprometer las fibras nerviosas pequeñas, manifestándose clínicamente con síntomas como parestesias, dolor neuropático y disfunción autonómica y progresando hacia una polineuropatía sensitivo-motora de distribución distal y simétrica. Las manifestaciones clínicas y neurofisiológicas dependen del estadio y el momento del diagnóstico. Conclusión: La amiloidosis por *TTR* asociada a polineuropatía es una enfermedad progresiva y de mal pronóstico. Por ello, es fundamental considerar los datos clínicos y neurofisiológicos de alarma que puedan orientar hacia su sospecha diagnóstica y manejo terapéutico.

**Evaluación Neurofisiológica de Polineuropatía en Paciente con Mieloma Múltiple: Un Caso con Distribución Atípica**

Christian Javier Hernández Aranda^1^, Sara Muniesa Lacasa^1^, Margely Abete Rivas^1^, María Martín Carretero^1^, África Bueno García^1^, Marta Ayuso Hernández^1^, José Miguel León Alonso-Cortés^1^

^1^Hospital Clínico Universitario de Valladolid, 47003 Valladolid, Castilla y León, España

**Introducción y Objetivos**: La polineuropatía periférica es una complicación frecuente y potencialmente limitante en pacientes oncológicos. Objetivos: Describir y analizar los hallazgos clínicos y neurofisiológicos en un paciente con mieloma múltiple y polineuropatía periférica. **Material y Métodos**: Estudio descriptivo retrospectivo de un caso clínico. (1) Datos clínicos: Masculino de 69 años diagnosticado en octubre 2023 de mieloma multiple IgA Lamba BenceJones, con t(11; 14) tratado con quimioterapia y radioterapia; (2) Estudios neurofisiológicos: Electroneurografía, Respuestas Tardías, Electromiograma. **Resultados**: Paciente desarrollo clínica sospechosa de polineuropatía periférica aguda incapacitante, con predominio en miembros superiores, durante el curso de su tratamiento con Bortezomib, realizándosele estudios neurofisiológicos diagnósticos. **Discusión/Conclusiones**: Los antecedentes y la clínica que presento el paciente, obligan a realizar un diagnóstico diferencial ampliado entre diversas etiologías. El estudio neurofisiológico fue determinante, evidenciando una polineuropatía axonal sensitiva moderada, exclusiva de MMSS. En cuanto a los hallazgos objetivados en MMII, se identificaron datos de denervación aguda (fibrilaciones) y crónica con ausencia de ondas F en MID, estos hallazgos compatibles con una radiculitis sobre una afectación radicular crónica en MMII; Datos compatibles con neuropatía tóxica por quimioterapia, sin excluir una posible neuropatía paraneoplásica asociada. Además, la ausencia de alteraciones neurofisiológicas características (como bloqueo de conducción, dispersión temporal, entre otras) fue crucial para el despistaje del síndrome de Guillain-Barré. La polineuropatía periférica es una complicación neurológica relevante en pacientes con mieloma múltiple. **Conclusión**: La polineuropatía en pacientes con mieloma múltiple tratados con esquemas modernos es multifactorial y debe ser evaluada cuidadosamente. El enfoque neurofisiológico no solo confirma el diagnóstico, sino que permite orientar etiología subyacente y guiar decisiones terapéuticas.

**Hallazgos Electrofisiológicos Por Intoxicación Colinérgica: A Propósito De Un Caso**

Pilar Rosario Martínez Martínez^1^, María Concepción Maeztu Sardiña^1^, Julián Vázquez Lorenzo^1^, Víctor Hugo Rubio Suárez^1^, Justo Marín Noguera^1^, Sara Giménez Roca^1^, Reetika Mukesh Baharani Baharani^2^

^1^Hospital Clínico Universitario Virgen de la Arrixaca, 30120 Murcia, España

^2^Hospital Clínico Morales Meseguer, Murcia, España, 30008 Murcia, España

**Introducción y Objetivos**: La intoxicación colinérgica consiste en una acumulación excesiva de acetilcolina en la terminación neuromuscular. Su causa más frecuente son los organofosforados, menos probable por toxinas halladas en setas. La clínica se caracteriza por manifestaciones colinérgicas: muscarínicas, nicotínicas y del sistema nervioso central. Se suceden tres formas en el tiempo: (1) Crisis colinérgica aguda (primeras horas), (2) Síndrome intermedio (SI, tras 24–96 horas), (3) Polineuropatía tardía. **Material y Métodos**: Estudio retrospectivo de un caso de intoxicación colinérgica valorado en HCUVA. **Resultados**: Varón ingresado en UCI tras parada cardiorrespiratoria reanimada. Presenta Glasgow de 3 y bradicardia, se intuba y más tarde se traqueostomiza. Presenta miosis, sialorrea y lagrimeo. Nivel de colinesterasa 10 veces por debajo de lo normal. Compatible con intoxicación colinérgica, en posible asociación a ingesta de setas alucinógenas sin confirmar la especie. Posterior mejoría en el Glasgow, con cuadriplejia e incapacidad para el destete. Se solicita electromiografía (EMG) 4 días tras el ingreso, con signos de severo trastorno de la transmisión neuromuscular al hallar en la estimulación repetitiva a altas frecuencias (30 Hz), en nervio mediano y cubital izquierdos, una respuesta de tipo decremental con caída de la amplitud del potencial del 55% en abductor pollicis brevis y 75% en eminencia hipotenar. Así como, ausencia de trazado voluntario muscular. Tras 15 días, en EMG de control, normalización de la estimulación repetitiva, coincidente con buena evolución clínica. **Discusión/Conclusiones**: La evolución temporal y los hallazgos en el EMG son compatibles con el SI, caracterizado por debilidad muscular junto a parálisis de los músculos respiratorios. Sucede por un trastorno postsináptico de la transmisión neuromuscular, que en la EMG se traduce en los hallazgos descritos en nuestro paciente. El estudio EMG de transmisión neuromuscular a bajas y altas frecuencias es fundamental en el diagnóstico y seguimiento de la intoxicación colinérgica.

**Laboratorio de Análisis de Movimiento, uso Novedoso del EMG de Superficie**

Alejandro José Soriano García^1^, Jorge Areán García^1^, Pedro Oliva Nacarino^1^, Paula García Carvajal^1^, María Beatriz Lozano Aragoneses^1^, Gianclaudio Dal Boni Gómez^1^, Consuelo Valles Antuña^1^

^1^Hospital Universitario Central de Asturias (HUCA), 33011 Oviedo, España

**Introducción y Objetivos**: Como es bien sabido la EMG mide la actividad eléctrica que los músculos producen tanto en reposo como durante el movimiento, utilizamos esta prueba en nuestras consultas, usualmente mediante aguja concéntrica o monopolar, para identificar el correcto funcionamiento de músculo y nervio, pudiendo discernir neuropatías, miopatías, trastornos neuromusculares entre otros. Sin embargo, no solemos tener experiencia en el uso de la EMG de superficie en los trastornos de movimiento, la cual este siendo utilizada de manera innovadora, junto con el análisis cinemático y cinético, en el estudio de la marcha. Los laboratorios de análisis de movimiento son unidades, que están comenzando a formarse en algunos hospitales a cargo de servicios dispares, cuyo objetivo es analizar e investigar de manera objetiva el desempeño motor del cuerpo humano con la intención de identificar alteraciones de las dinámicas de la marcha y poder influir en una mejor decisión terapéutica. El objetivo de esta comunicación es dar a conocer estas unidades novedosas, su utilidad, el papel que tiene la EMG de superficie en ellas y su potencial relación con la Neurofisiología Clínica. **Material y Métodos**: Documentación basada en los libros “Análisis del Movimiento Humano para Profesionales de la Salud” y “Clinical Gait Analysis theory and practice”, recomendados desde el laboratorio de análisis del movimiento del HUCA, desde el que se reporto la experiencia e información práctica y teórica. **Resultados**: La experiencia del laboratorio de movimiento, aunque prototípica, esta arrojando una gran cantidad de datos para analizar que reportan un mejor manejo terapéutico de los pacientes evaluados por la unidad, objetivable en estudios de control, así como una mayor percepción de mejoría por parte de los pacientes respecto a aquellos que no pasan por la misma. **Discusión/Conclusiones**: Con esta comunicación se pretende dar a conocer estas unidades y abrir un debate sobre la implicación que como especialidad podemos tener en la evaluación de la patología del movimiento.

**Rendimiento Ecográfico en el Diagnóstico y Graduación del Síndrome del Túnel del Carpo Frente a la Electroneurografía**

Alejandro José Soriano García^1^, Gianclaudio Dal Boni Gómez^1^, Jesús González Rato^1^, Paula García Carvajal^1^, María Beatriz Lozano Aragoneses^1^, Luis Santoveña González^1^, Consuelo Valles Antuña^1^

^1^Hospital Universitario Central de Asturias (HUCA), 33011 Oviedo, España

**Introducción y Objetivos**: Se ha realizado un estudio descriptivo y transversal de exactitud diagnostica a pacientes con sospecha de síndrome del túnel del carpo (STC), con el objetivo de comprobar el rendimiento diagnóstico de los diferentes parámetros ecográficos en comparación con la electroneurografía (gold standard). Con ello se pretende demostrar la utilidad de la ecografía neuromuscular en la práctica clínica de la especialidad de neurofisiología clínica. **Material y Métodos**: En el estudio se incluyeron pacientes con sospecha de patología del STC con un rango de edad entre 18 y 80 años. Se excluyeron aquellos pacientes con antecedente de diabetes mellitus, otras lesiones de nervio periférico, antecedentes de cirugía del TC o fractura de muñeca. La adquisición de las imágenes fue realizada de forma ciega e independiente. Se realizaba en primera instancia la prueba ecográfica, registrando el área de sección transversal en diferentes puntos del trayecto del nervio mediano, así como el diámetro transverso y anteroposterior del nervio mediano a su salida del TC. Con estos datos se calculaba los parámetros ultrasonográficos de ratio y diferencia de edematización muñeca/antebrazo y ratio de adelgazamiento, con los que se determinaba si había patología del STC. El AST (área de sección transversal) del nervio en la entrada del TC y el índice MA (muñeca antebrazo) lo utilizamos para graduar los diferentes estadios del STC en función de valores de corte constatados por otros estudios. Una vez emitido el diagnostico ecográfico se realizaba la ENG, con la que se comparaba la presencia o ausencia de STC y su graduación. **Resultados**: El estudio ha inferido, con un intervalo de confianza del 95%, una sensibilidad de 0,72, especificidad de 0,63, VPP de 0,77 y un VPN de 0,55 para detectar STC para el AST a la entrada del TC, siendo este el parámetro ecográfico de mayor rentabilidad diagnóstica. **Discusión/Conclusiones**: El estudio concluye que la ecografía no alcanza valores estadísticos suficientes para considerarla como prueba diagnóstica de cribado eficaz.

**Numb Chin Syndrome Secundario a Cirugía Ortognatica. A Propósito de un Caso**

Sonia Montilla Izquierdo^1^, Carlos Castañeda Cabrero^1^, Esteban Peña Llamas^1^, Cristina Fernández García^1^, Clara Fernández Cortés^1^, Andrea Gómez Moroney^1^, Madeleyn Rodríguez Jiménez^1^

^1^Hospital Universitario La Moraleja, 28050 Madrid, España

**Introducción y Objetivos**: El síndrome de hipoestesia mentoniana es debido a la lesión del nervio alveolar inferior (mentoniano). Aunque las causas más comunes son banales como traumatismos, extracciones dentarias e infecciones, la asociación con neoplasias malignas no es despreciable. **Material y Métodos**: Varón de 44 años sin antecedentes médicos de interés. No tratamiento habitual. Cirugía ortognática para control de Apnea obstructiva del sueño. Desde entonces movimientos involuntarios de segundos de duración, una o varias veces al día, en el tercio inferior de la cara y la mandíbula que le dificulta la articulación del lenguaje por incapacidad para mover adecuadamente la lengua. Muestra vídeo que visualizan y confirman los neurólogos en la consulta. **Resultados**: Pruebas complementarias: analítica completa incluido frotis para neuroacantocitosis que muestra como único hallazgo anormal discreto aumento de CPK. TAC y RMN craneal sin hallazgos relevantes. Se plantea diagnóstico diferencial entre distonia oromandibular y trastorno conversivo. Se realiza estudio neurofisiológico explorándose electroneurografía de ambos nervios faciales y electromiograma de los músculos mentalis y vientre anterior de ambos digástricos. Se aprecia un patrón neurógeno deficitario, sin datos de denervación activa asociada, en vientre anterior del músculo digástrico izquierdo. Este dato pudiera estar en relación con una lesión axonal crónica del nervio alveolar inferior izquierdo de la rama mandibular del trigémino. Se inicia tratamiento con anticolinérgicos con respuesta parcial. **Discusión/Conclusiones**: Las parestesias faciales son un motivo de consulta relativamente frecuente en Neurología. Aunque la mayoría de los casos son cuadros banales, hacer pruebas de imagen, analíticas y neurofisiológicas pertinentes es obligado por la posibilidad de asociar patologías más severas. En nuestro caso la posible asociación con neoplasias malignas hace necesario la realización de un seguimiento mediante control de forma periódica para valorar la aparición de nueva sintomatología.

**Hallazgos Neurofisiológicos en la Encefalomielitis Progresiva con Rigidez y Mioclonías**

Olga Fedirchyk Tymchuk^1^, Marta Villadóniga Zambrano^1^, Liliana González Rodríguez^1^, María Carolina Chinchilla Haylock^1^, Valeria Vanessa Gallo Rivero^1^, Juan Luis Chico García^1^, Lidia Cabañes Martínez^1^

^1^Hospital Universitario Ramón y Cajal, 28034 Madrid, España

**Introducción y Objetivos**: La encefalomielitis progresiva con rigidez y mioclonías (PERM) es una forma grave y poco frecuente del síndrome de la persona rígida (SPR), caracterizada por rigidez muscular progresiva, espasmos dolorosos, hiperekplexia, mioclonías, síndrome troncoencefálico y disautonomía. La etiología es inmunomediada, habiéndose descrito altos niveles de anticuerpos antiGlyR yo antiGAD65. Sin tratamiento, tiene un pronóstico infausto. **Material y Métodos**: Descripción del caso clínico. **Resultados**: Varón de 47 años remitido a nuestro hospital para valorar timectomía. Desde hacía 1 mes presentaba un cuadro de oftalmoparesia, disfagia, hiperreflexia, hiperekplexia, disautonomía y mioclonías, así como rigidez en miembros inferiores (MMII). Un estudio inicial mediante RM del neuroeje, punción lumbar y analítica con autoinmunidad fue normal. El estudio de estimulación repetitiva y jitter fue normal. Días después se solicitó un nuevo ENF que mostró una alteración de SNA con normalidad de los estudios de conducción nerviosa. El estado clínico del paciente empeoró, con una mayor rigidez y espasmos en MMII. Se detectaron anticuerpos anti-GAD65 en suero y LCR. Se realizó un nuevo ENF, mostrando actividad muscular contínua en reposo en músculos agonista-antagonista de MMII, exacerbada con estímulo táctil, así como signos de hiperexcitabilidad en el estudio de los reflejos de tronco. Se inició tratamiento con IgIV y posteriormente corticoterapia, con mejoría clínica. El paciente fue intervenido y se confirmó el diagnóstico de timoma, mostrando una evolución favorable. **Discusión/Conclusiones**: El SPR es un espectro de trastornos neurológicos de etiología autoinmune, con el hallazgo más característico de actividad muscular contínua en los músculos afectos. El PERM es una forma grave de SPR que, debido a la gravedad de sus síntomas puede tener una evolución fulminante. Es importante conocer todas las formas clínicas de esta entidad, así como los hallazgos en los ENF, pues un diagnóstico y tratamiento precoces mejoran el pronóstico de estos pacientes.

**Transformación del Proceso Asistencial del Síndrome del Túnel Carpiano (STC). Evolución y Datos de los Años 2023 y 2024**

Blanca Díaz Montoya^1^, Inmaculada López Gutiérrez^2^, José Carlos Fernández Ferro^3^, Javier Cervera Irimia^1^, Carmelo Fernández García^1^

^1^Hospital Universitario Fundación Jiménez Díaz, 28040 Madrid, España

^2^Servicio Andaluz de Salud, 41071 Sevilla, España

^3^Hospital Universitario Rey Juan Carlos, 28933 Madrid, España

**Introducción y Objetivos**: Hacer seguimiento del proceso asistencial desarrollado en el Servicio de Neurofisiología Clínica (NFC) de los Hospitales Públicos de Madrid (Fundación Jiménez Díaz, Rey Juan Carlos, Infanta Elena y General de Villalba) junto a los servicios de Traumatología (TRA), Neurología (NRL) y Rehabilitación (REH) en la estandarización de las trayectorias de los pacientes con STC procedentes de atención primaria (AP) y hospitalaria (AH) con el fin de mejorar la calidad asistencial, la experiencia del paciente, haciendo un uso responsable de los recursos. **Material y Métodos**: Se siguió el protocolo iniciado en 2022, que establece la valoración por NFC como primer paso en el proceso asistencial del STC. El neurofisiólogo clínico toma decisiones basadas en el resultado del electroneuromiograma pudiendo iniciar tratamiento conservador o derivar al paciente al servicio correspondiente. El proceso se automatizó con la implementación de vías de derivación protocolizadas desde AP y AH, integración de algoritmos, y consultas digitales, lo que permitió una gestión más eficiente del flujo asistencial, desde la entrada en el circuito hasta el cierre del mismo. **Resultados**: En los años 2023 y 2024 se atendieron 11.432 pacientes en la consulta de NFC con sospecha de STC. Una media del 23,78% fueron dados de alta directamente, tanto el diagnóstico como el inicio del tratamiento conservador se realizaron en una única visita hospitalaria. El 76,28% restante fue derivado a TRA, NRL o REH para continuar su atención. De los 2669 pacientes con afectación moderada o severa que fueron derivados hacia TRA un 36,52% fue incluido directamente en lista de espera quirúrgica, con un seguimiento protocolizado y digital posterior a la intervención. **Discusión/Conclusiones**: El conocimiento de esta trayectoria estandarizada por parte de los profesionales optimiza los circuitos de derivación, permitiendo un modelo de trabajo eficiente y orientado al paciente. Todo ello permite una mejora del proceso asistencial de STC, la satisfacción de pacientes y profesionales.

**Relevancia de los Estudios de Conducción Nerviosa en la Enfermedad de Charcot-MarieTooth, en la era de la Genética**

Thatiana Rossi Meza Gaspar^1^, María Carolina Chinchilla Haylock^1^, Valeria Vanessa Gallo Rivero^1^, Marta Villadóniga Zambrano^1^, Olga Fedirchyk Tymchuk^1^, Lidia Cabañes Martínez^1^, Ignacio Regidor BaillyBailliere^1^

^1^Hospital Universitario Ramon y Cajal, 28034 Madrid, España

**Introducción y Objetivos**: La enfermedad de Charcot-Marie-Tooth (CMT) es la forma más frecuente de neuropatía hereditaria que clásicamente se manifiesta con debilidad distal, alteraciones sensitivas, arreflexia y pie cavo. El diagnóstico inicial es principalmente clínico y debe implicar la exclusión de otras neuropatías o enfermedades neuromusculares con clínica similar. El estudio neurofisiológico puede no ser esencial en casos clínicamente sugestivos y con historia familiar compatible. Sin embargo, cuando la sospecha diagnóstica no está bien definida, en ocasiones la consulta de neurofisiología es el primer momento en el que el paciente es explorado neurológicamente. **Material y Métodos**: Se describen 7 casos remitidos a nuestra consulta entre los años 2021 y 2025 con sospechas diagnósticas diversas, pero nunca con sospecha de CMT, en las que el estudio de conducción nerviosa (ECN) fue sugestivo de una neuropatía hereditaria. **Resultados**: Edades comprendidas entre 34 y 64 años, predominio de mujeres (n = 4). La mayoría de pacientes procedencia de traumatología (n = 5). Los motivos de consulta más comunes fueron lumbalgia y deformidad en miembros inferiores. En todos los casos el ECN mostró una polineuropatía sensitivo-motora simétrica, de claro componente desmielinizante (velocidades de conducción marcadamente disminuidas, importante dispersión temporal), y se sugirió valoración por Neurología. En el seguimiento posterior, en cinco de los casos se confirmó con estudio genético (se usó panel genético de 86 genes de CMT) un CMT1A, en uno de los casos la genética fue negativa y en los dos casos restantes el estudio no fue realizado hasta la fecha. **Discusión/Conclusiones**: Los ECN proporcionan datos relevantes para el diagnóstico de la enfermedad de CMT, por lo que son útiles en todos los pacientes con sospecha clínica. Sin embargo, en algunos casos los signos pueden no ser reconocidos en un primer momento si no se hace una exploración neurológica adecuada y completa, y en estos casos el ECN es esencial para el diagnóstico.

**Utilidad de la Electromiografía en la Caracterización de Dermatomiositis antiTIF1γ: Descripción de una Serie de Casos**

María Carolina Chinchilla Haylock^1^, Olga Fedirchyk Tymchuk^1^, Lidia Cabañes Martínez^1^, Marta Villadóniga Zambrano^1^, Ignacio Regidor^1^

^1^Hospital Universitario Ramón y Cajal, 28034 Madrid, España

**Introducción y Objetivos**: La dermatomiositis anti-TIF1γ representa un subtipo clínico característico poco probable que afecta a individuos mayores de 50 años y debe alertar sobre la posibilidad de neoplasias ocultas. Clínicamente se puede observar lesiones cutáneas clásicas, sin embargo la presencia de afectación muscular es variable. La electromiografía representa una herramienta de apoyo tanto en el enfoque diagnóstico inicial como evolutivo. Además, su sensibilidad varía según la fase de la enfermedad y la expresión clínica. Describir una serie casos con dermatomiositis anti-TIF1γ asociada a distintos tipos de cáncer y evaluar el papel de la electromiografía en la orientación diagnóstica. **Material y Métodos**: Se presentan cuatro pacientes diagnosticados de dermatomiositis anti-TIF1γen el contexto de neoplasias sólidas. Se analizan características clínicas, oncológicas, serológicas y hallazgos electromiográficos. **Resultados**: Caso 1 varón de 67 años diagnosticado de cáncer renal y debilidad proximal y ausencia de lesiones cutáneas. Caso 2 varón de 82 años diagnosticado de cáncer gástrico, debilidad proximal y lesiones cutáneas. Caso 3 mujer de 53 años diagnosticada de cáncer de mama y clínica de debilidad proximal y lesiones cutáneas. Caso 4 mujer de 45 años, diagnosticada de cáncer de tiroides sin clínica muscular ni cutánea. En los cuatro casos se confirmó malignidad dentro del período típico de asociación paraneoplásica (<3 años) y serología positiva con anticuerpos anti-TIF1γ. El electromiograma (EMG) mostró hallazgos variables: dos casos con patrón miopático inflamatorio franco y dos con alteraciones sutiles (presencia de inestabilidad de membrana) sin patrón miopático. **Discusión/Conclusiones**: El EMG es una herramienta útil en la valoración de la afectación muscular en pacientes con dermatomiositis debido a las implicaciones terapéuticas y pronósticas que supone. En nuestra serie de casos con dermatomiositis anti-TIF1 no hemos observado ningún hallazgo electromiográfico específico que lo haga diferente de otro tipo de miopatías inflamatorias.

**Papel del Electromiograma en la Plexopatía Lumbar Iatrogénica: Revisión de Casos en la Última Década**

Maria Carolina Chinchilla Haylock^1^, Olga Fedirchyk Tymchuk^1^, Thatiana Meza Gaspar^1^, Marta Villadóniga Zambrano^1^, Lidia Cabañes Martínez^1^, Ignacio Regidor^1^

^1^Hospital Universitario Ramón y Cajal, 28034 Madrid, España

**Introducción y Objetivos**: La afectación del plexo lumbar es una complicación poco frecuente pero significativa, asociada a diversos procedimientos quirúrgicos. Su prevalencia depende del tipo de intervención, el abordaje anatómico, posición del paciente y la duración de la cirugía. El diagnóstico puede ser complejo debido a la variabilidad clínica y a la superposición con otras patologías neuromusculares. Analizar la relación entre las diferentes intervenciones quirúrgicas y la aparición de neuropatía femoral o plexopatía lumbar iatrogénica, destacando el papel del electromiograma en el proceso diagnóstico. **Material y Métodos**: Se realizó una revisión retrospectiva de casos clínicos documentados en los últimos 10 años, en los que se diagnosticó una neuropatía femoral o plexopatía lumbar tras procedimientos quirúrgicos traumatológicos, urológicos, ginecológicas, endovasculares y abdominales. Se analizó la correlación entre las manifestaciones clínicas y los hallazgos electromiográficos. **Resultados**: En los 66 casos estudiados, el electromiograma permitió confirmar el diagnóstico con un tiempo medio de 13 meses y establecer la severidad del daño. El promedio de edad fue de 60 años con mayor prevalencia en varones (n = 35) y en el lado derecho (n = 37). La cirugía más frecuentemente asociada con lesión del nervio femoral fue la prótesis total de cadera (n = 15). En 9 casos se diagnosticó una plexopatía lumbar, siendo la cirugía abdominopélvica (n = 6) la más asociada. **Discusión/Conclusiones**: La afectación de algunas de las estructuras del plexo lumbar debe considerarse en el diagnóstico diferencial de debilidad e hipoestesia proximal y dolor inguinal postoperatorio. El electromiograma es esencial en la evaluación de estos pacientes, permitiendo una identificación temprana y precisa de la lesión, lo cual es clave para una adecuada orientación diagnóstica, intervención oportuna según precise y un mejor pronóstico funcional.

**Neuropatía Axonal Sensitiva de los Nervios Mediano, Cubital y Radial en Paciente con FAVI Húmero-Basílica Derecha**

Joseba Soto Ibáñez^1^, Beatriz García López^1^, María Carmen Lloria Gil^1^, Olga Pérez Gil^1^, Boris Alfonso Orozco Jiménez^1^, Diego Francisco Peña Olaya^1^, Alina Havrylenko Vynograndnyk^1^

^1^Hospital Universitario de Burgos, 09006 Burgos, Castilla y León, España

**Introducción y Objetivos**: Paciente de 39 años en seguimiento por el servicio de Cirugía Vascular por fístula arterio-venosa (FAVI) húmero-basílica es remitida a nuestras consultas por presentar clínica sugerente de afectación del nervio Mediano. El diagnóstico de sospecha inicial es el de axonotmesis versus robo vascular, por lo que se solicita estudio electromiográfico (EMG). **Material y Métodos**: Se estudian las conducciones motoras y sensitivas de los nervios Mediano y Radial bilateral y Cubital derecho utilizando electrodos de superficie. Se estudian también músculos dependientes de los nervios Mediano, Cubital y Radial (m. Flexor radial del carpo, Flexor cubital del carpo, extensor propio del índice y Extensor común de los dedos) utilizando un electrodo de aguja coaxial. **Resultados**: Se obtienen datos de neuropatía en nervios Mediano, Cubital y Radial, en su vertiente sensitiva en el momento actual, de carácter axonal y de grado severo. **Discusión/Conclusiones**: La disociación de la afección sensitiva versus motora (indemne en el momento de la exploración), con el mantenimiento de amplitudes distales y la afectación sensitiva del Radial (a priori fuera del campo quirúrgico de la intervención), hacen pensar en un mecanismo de lesión isquémico, debido a robo vascular que pudiera progresar en el futuro afectando a la vertiente motora de dichos nervios.

**Detección Precoz del Síndrome de Guillain-Barré en Niños, Hallazgos EMG-ENG y Revisión de la Literatura**

Raidili Cristina Mateo Montero^1^, Catalina Viña Jurado^2^, Ingrid Rivadeneira^2^, Francisco González de la Rosa^2^, Miguel Á Hernández Latorre^2^

^1^Neurotoc, HM Now Delfos, HM Nens, 08023 Barcelona, España

^2^HM Nens, 08009 Barcelona, España

**Introducción y Objetivos**: El síndrome de Guillain-Barré (SGB) en niños es la causa más frecuente de parálisis flácida aguda en la infancia aunque su incidencia es baja. En niños el diagnóstico temprano puede ser complejo debido a que los síntomas iniciales son inespecíficos (dolor e irritabilidad más que debilidad franca), por este motivo el EMG y ENG son claves para la su detección precoz. El objetivo es describir los hallazgos electrofisiológicos característicos del SGB en niños atendidos en nuestro hospital y compararlos con los reportados en la literatura. **Material y Métodos**: Estudio retrospectivo del 6 pacientes (4 niños y 2 niñas de entre 2 y 11 años de edad) diagnosticados de SGB; todos fueron evaluados con EMG-ENG incluyendo el las ondas F, Reflejo H y Blink reflex los primeros 7 días del inicio de los síntomas. **Resultados**: El primer EMG-ENG se realizó con un promedio de 4 días tras el inicio de los síntomas y los hallazgos principales fueron: latencia de la onda F aumentada, con una media de más 5 ms en relación con los valores normales para la edad y la altura, presente en todos los casos; latencia aumentada de reflejo H en 3 pacientes. Hallazgos sugerentes de la variante axonal aguda motora (AMAN) en el 50% de los casos y resultados asimétricos en el 50% de los casos sugiriendo presentaciones atípicas. **Discusión/Conclusiones**: La prolongación de la onda F y del reflejo H son hallazgos precoces y fiables en el SGB infantil. Otras alteraciones, como aumento de la latencia y la leve disminución de la velocidad de conducción, suelen aparecer en etapas más avanzadas. El SGB debe considerarse como diagnóstico diferencial ante cualquier parálisis flácida aguda o progresiva con arreflexia, incluso en casos con debilidad asimétrica. Nuestros datos revelan una frecuencia significativa de la variante AMAN y de hallazgos asimétricos, lo que sugiere una presentación clínica más diversa de lo que se describe habitualmente.

**Neuromiotonía Focal con Anticuerpos Anticanales de Potasio. A Propósito de un Caso**

Gonzalo Díaz Cano^1^, Nelson Cuéllar Ramos^1^, Verónica Mendoza Parra^1^, Alba González Martínez^1^, Marta Escribano Muñoz^1^, Óscar Garnés Camarena^1^, José Manuel Corredera Rodríguez^1^

^1^Hospital Universitario Fundación Jiménez Díaz, 28040 Madrid, España

**Introducción y Objetivos**: Los síndromes de hiperexcitabilidad de nervio periférico son un grupo de trastornos caracterizados por rigidez muscular, calambres y sacudidas musculares, con actividad eléctrica anormal involuntaria en el EMG. Los hallazgos neurofisiológicos en estos trastornos se solapan y a menudo generan confusión. La forma de presentación como neuromiotonía focal es muy poco frecuente, pudiendo confundirse con patología reumatológica. En los pocos casos publicados, es común el antecedente de enfermedad pulmonar obstructiva crónica en tratamiento con agonistas beta-2 adrenérgicos. **Material y Métodos**: Presentamos el caso de una mujer de 72 años, asmática en tratamiento con broncodilatadores y corticoides. Presenta flexión forzada, dolorosa, del tercer dedo de la mano izquierda, de meses de evolución, que persiste durante el sueño. Es valorada por Traumatología, que solicita EMG y RM. **Resultados**: La RM mostró una rotura de los tendones de los extensores del brazo. En el EMG se observaron descargas que se interpretaron como posibles neuromiotonías. Ante la sospecha de un síndrome de hiperexcitabilidad de nervio periférico, se solicitaron anticuerpos anticanales de potasio, que dieron resultado positivo, diagnosticándose de un síndrome de Isaacs con presentación focal. Se trató con corticoides y con inmunoglobulinas, sin mejoría. **Discusión/Conclusiones**: Los síndromes de neuromiotonía focal son muy poco frecuentes. Los escasos casos descritos en la literatura comparten presentación en un brazo, presencia de descargas electromiográficas de alta frecuencia, anticuerpos anticanales de potasio positivos, por lo que se consideran una variante del síndrome de Isaacs, así como antecedente de enfermedad pulmonar crónica en tratamiento con broncodilatadores. En varios casos se describe mala respuesta al tratamiento.

**Lesión del Plexo Lumbosacro, una Complicación Rara Pero Invalidante en el Parto. A Propósito de un Caso**

David Cañizo García^1^, Inmaculada Rodríguez Ulecia^1^, Octavio Jiménez Vega^1^, María Jesús Alemany Rodríguez^1^, Juan Francisco García Granado^1^, Amanda Labrador Rodríguez^1^

^1^Hospital Universitario de Gran Canaria Doctor Negrín, 35010 Las Palmas de Gran Canaria, España

**Introducción y Objetivos**: La afectación neurológica tras el parto es infrecuente, con una incidencia menor al 2%, que puede pasar desapercibida si no se realiza una evaluación neurofisiológica dirigida. Dentro de esta, el uso de electromiografía (EMG), incluyendo la exploración del suelo pélvico, constituye una herramienta esencial para el diagnóstico topográfico y funcional en mujeres con síntomas neurológicos postparto, especialmente cuando existen déficits motores y alteraciones esfinterianas. **Material y Métodos**: Presentamos el caso de una paciente que, como resultado de un parto instrumental complicado, desarrolló una marcada debilidad en la flexión dorsal y eversión del pie izquierdo e hipoestesia de la cara lateral de la pierna, además de incontinencia urinaria sin sensación de urgencia. Fue derivada a nuestra consulta para un estudio neurofisiológico que incluyó electroenurografía (ENG) sensitivo/motora y EMG, en territorios del nervio femoral y ciático, así como ENG, EMG de esfínter anal, PESS de pudendo y reflejo bulbocavernoso. **Resultados**: La ENG mostró una disminución de amplitud motora del nervio peroneo profundo y ausencia de potencial sensitivo del peroneo superficial izquierdos, y la EMG objetivó denervación aguda en los músculos: tensor de la fascia lata, tibial anterior, peroneo largo y gastrocnemio medial, sin afectación paravertebral, y el estudio de suelo pélvico mostró un aumento de la duración y amplitud de los PUMs sin denervación, en el territorio del nervio pudendo izquierdo, lo que permitió confirmar una lesión extensa del plexo lumbosacro izquierdo. Se derivó precozmente a rehabilitación con buena evolución. **Discusión/Conclusiones**: Este caso subraya la necesidad de realizar estudios neurofisiológicos sin dilación, en mujeres con partos traumáticos, que debería incluir un estudio de suelo pélvico en aquellas pacientes con clínica compatible con lesión a este nivel. Dentro del mismo el uso de la EMG permite no solo establecer la topografía de la lesión, sino guiar tratamientos rehabilitadores, facilitando un abordaje precoz.

**Un Déficit Motor Inesperado**

María de los Ángeles Velázquez Gómez^1^, Carlos José Velásquez Rodríguez^1^, Roberto Ocón Quintial^1^, José Luis Fernández Torre^1^

^1^Hospital Universitario Marqués de Valdecilla, 39010 Santander, España

**Introducción y Objetivos**: La monitorización neurofisiológica intraoperatoria (MNIO) es imprescindible durante la cirugía de un tumor supratentorial en áreas motoras o próximo a ellas, con el objetivo de conseguir la mayor resección tumoral posible y de detectar cambios en las funciones neurológicas. Desafortunadamente no siempre es posible, de modo que hay que considerar algunas situaciones en las que el paciente puede despertarse con un déficit motor imprevisto. **Material y Métodos**: Describimos los hallazgos de la monitorización de un paciente de 44 años durante la exéresis de un glioblastoma frontal derecho, mediante registro de potenciales evocados somatosensoriales (PESS) de miembros superiores e inferiores y potenciales evocados motores (PEM), tanto transcraneales de hemicara izquierda, miembros superiores e inferiores, como corticales (tira de 4 electrodos sobre área motora de miembro superior izquierdo). Durante la estimulación cortical directa, el paciente presenta una crisis focal motora autolimitada de unos 20 segundos de duración, similar a las que motivaron el diagnóstico, tras lo cual se objetiva un aumento del umbral de estímulo. El incremento de los parámetros de estimulación consigue evocar PEM en todos los músculos; sin embargo, el paciente despierta con una hemiplejia izquierda. **Resultados**: Al aumentar los parámetros de estimulación para compensar el aumento de umbral, también evocan PEM por estimulación subcortical. La evolución del paciente y las pruebas complementarias orientan a la coexistencia, en este caso, de dos causas concomitantes: parálisis de Todd en miembro superior izquierdo y una lesión del área motora de miembro inferior izquierdo. **Discusión/Conclusiones**: Existen situaciones en las cuales un paciente puede despertar con un déficit motor a pesar de haberse conseguido evocar PEMs en todos los músculos registrados al cierre quirúrgico y haberse realizado una correcta técnica de MNIO.

**Afectación de Pares Craneales en Charcot Marie Tooth**

María de los Ángeles Velázquez Gómez^1^, Roberto Ocón Quintial^1^, José Luis Fernández Torre^1^

^1^Hospital Universitario Marqués de Valdecilla, 39010 Santander, España

**Introducción y Objetivos**: La enfermedad de Charcot-Marie-Tooth (CMT) engloba un espectro heterogéneo de polineuropatías sensitivo-motoras hereditarias, con un fenotipo típico consistente en atrofia y debilidad de músculos distales, asociando deformidades en los pies. Aunque menos frecuentemente descrita, en CMT también podemos encontrar afectación de pares craneales (PC), variable según el subtipo. **Material y Métodos**: Se presenta el caso de una paciente de 32 años con antecedente de CMT (neuropatía relacionada con la proteína mielínica zero, CMT1B probable), en la cual realizamos monitorización intraoperatoria durante la liberación del nervio trigémino derecho, en contexto de una neuralgia del trigémino. Se monitorizan, a parte del electromiograma de barrido libre, potenciales evocados somatosensoriales (PESS) de miembros superiores e inferiores, potenciales evocados auditivos de tronco (PEAT), reflejo de parpadeo, reflejo adductor laríngeo (RAL) y potenciales evocados motores (PEM) de músculos dependientes de nervio facial, trigémino y vago, así como de miembros superiores e inferiores a modo de control. **Resultados**: El estudio de monitorización neurofisiológica muestra PEAT, RAL y PEM en cuerdas vocales con respuestas bien definidas y reproducibles al inicio de la cirugía que se mantienen sin cambios al cierre. En el resto de señales monitorizadas no se consigue evocar respuestas consistentes. **Discusión/Conclusiones**: El CMT1B puede asociar afectación de los PC V y VII (nervios trigémino y facial), dando clínica en algunos pacientes de neuralgia del trigémino o espasmo hemifacial, lo que se traduce en una mayor incidencia de estas patologías en este grupo de pacientes.

**Optimización de Resultados en Espasticidad Infantil Mediante Rizotomía Dorsal Selectiva y Monitorización Intraoperatoria**

Gabriela Valeria Solís Chávez^1^, Manuel Luján Bonete^2^, David Mansilla Lozano^2^, María Esperanza Marín^2^, María Polvorosa Cáceres^1^

^1^Hospital Universitario de La Princesa, 28006 Madrid, España

^2^Hospital Universitario Infantil Niño Jesús, 28009 Madrid, España

**Introducción y Objetivos**: La rizotomía dorsal selectiva (RDS) es un procedimiento quirúrgico con beneficios significativos en el tratamiento de la espasticidad. No obstante, para maximizar su eficacia y minimizar el riesgo de secuelas funcionales irreversibles, es fundamental realizarla junto con la monitorización neurofisiológica intraoperatoria (MION). Se describen las características clínicas pre y postoperatorias de pacientes sometidos a RDS, así como la técnica de MION empleada en nuestro centro. **Material y métodos**: Se presentan dos casos de pacientes pediátricos de 8 y 10 años respectivamente, con diferentes etiologías de espasticidad, intervenidos en 2023 y 2024 en el Hospital Universitario Infantil Niño Jesús. En ambas intervenciones, se utilizó la técnica de MION descrita por Fasano. Inicialmente, se realiza un mapeo anatómico mediante estimulación de raíces ventrales para su identificación y preservación; posteriormente, se lleva a cabo un test de excitabilidad sobre las raíces dorsales. En función de la respuesta muscular obtenida, se procede a la rizotomía selectiva de aquellas raíces dorsales consideradas patológicas. **Resultados**: En ambos casos, la MION se realizó sin complicaciones. Tanto la monitorización de vías largas, como el reflejo bulbocavernoso, se mantuvieron estables durante toda la intervención. Se identificaron adecuadamente las raíces patológicas, respetando aquellas involucradas en reflejos sacros. Los pacientes mostraron una mejoría clínica significativa, evidenciada por la evolución funcional y en algunos parámetros del análisis cinemático de la marcha. **Discusión/Conclusiones**: Dada la heterogeneidad clínica de los pacientes candidatos a RDS, la incorporación sistemática de MION se justifica plenamente, ya que proporciona una guía intraoperatoria precisa para el neurocirujano, especialmente en aquellos casos de una anatomía medular distorsionada. Se destaca la importancia de establecer sesiones multidisciplinarias que faciliten la selección de nuevos candidatos susceptibles de beneficiarse de este procedimiento.

**Utilidad de PESS de Nervio Safeno en Monitorización de Raíces Lumbares Altas: Tres Casos con Implicaciones Pronósticas**

Marián Nicolás Vela^1^, Anna Rojas Contreras^1^, María de los Ángeles Sánchez Roldán^1^, Daniela Santa Cruz Arévalo^1^

^1^Hospital Universitario Vall D´Hebron, 08035 Barcelona, España

**Introducción y Objetivos**: Los abordajes lateral y anterior para la fusión intersomática lumbar (OLIF, LLIF, XLIF y ALIF) suponen un riesgo de lesión iatrogénica elevado para el plexo lumbar. La monitorización intraoperatoria (MIO) con potenciales evocados somestésicos (PESS) del nervio safeno permite evaluar la integridad sensitiva de raíces lumbares altas (L3–L4), no accesibles mediante los PESS clásicos del tibial posterior/peroneo. Presentamos 3 casos en los que el registro de safeno permitió detectar precozmente eventos intraoperatorios lesivos. **Material y Métodos**: Se incluyeron tres pacientes intervenidos mediante abordajes XLIF o ALIF con MIO multimodal incluyendo PES de safeno. Se realizó estimulación distal a 15 cm del maléolo medial y registro cortical. Se consideraron significativos descensos de amplitud ≥50%. Los cambios intraoperatorios fueron correlacionados con las maniobras quirúrgicas y la clínica postoperatoria. **Resultados**: Caso 1: Caída bilateral de amplitud de hasta el 60% de PES de safeno coincidiendo con la colocación de cajas intersomáticas, correlacionado con parestesias postoperatorias. Caso 2: Decremento bilateral del 50% durante la separación de grandes vasos que se normalizaron al retirar los separadores, sin déficits clínicos asociados. Caso 3: Caída transitoria del 80% del PES derecho en relación con posible tracción radicular por los separadores, con recuperación tras la recolocación, sin clínica postoperatoria. **Discusión/Conclusiones**: La monitorización del nervio safeno proporciona información util sobre la funcionalidad del plexo lumbar superior, siendo clave en cirugías con riesgo para raíces L3–L4. Frente a la baja sensibilidad del EMG libre, los PESS de safeno han demostrado mayor capacidad para detectar alteraciones intraoperatorias que puedan revertirse evitando secuelas. Nuestros casos ilustran cómo su registro permitió medidas correctoras y ayudó a la prevención de déficits postoperatorios. La inclusión sistemática del PESS de safeno en MIO en cirugía lumbar debería considerarse como estándar emergente.

**Neuromonitorización en Fusión Intersomática por Abordaje Lateral Transpsoas (“XLIF”) en Escoliosis Degenerativa**

Juan Sebastián Aller Álvarez^1^, Trinidad Blanco Hernández^1^, Ana Piedad Arias Balsalobre^1^, Fernando Prieto Prieto^1^, Sara Cors Serra^1^, Miguel Sanfeliu Giner^1^

^1^Consorcio Hospital General Universitario de Valencia, 46014 Valencia, España

**Introducción y Objetivos**: La escoliosis degenerativa adquirida es de predominio lumbar y se asocia a dolor refractario. La fusión intersomática por abordaje lateral transpsoas, “Extreme lateral interbody fusions” (XLIF), es un procedimiento mínimamente invasivo con menor riesgo vascular, pero con riesgo de daño del plexo lumbar. En nuestro centro comenzó a realizarse en 2014. Presentamos los resultados de nuestras monitorizaciones intraoperatorias (MIO). **Material y Métodos**: Análisis retrospectivo de pacientes intervenidos mediante XLIF en dos tiempos (decúbito lateral y decúbito prono), se excluyeron las escoliosis juveniles y los pacientes intervenidos previamente. Se realizaron potenciales evocados motores mediante estimulación eléctrica transcraneal (TcMEP) y electromiografía (EMG). En un 30% de los pacientes se les realizó potenciales evocados somatosensoriales (SSEP) de nervios safenos. **Resultados**: Se incluyeron 20 pacientes, 100% mujeres y 95% tenían ≥65 años (rango 56–79). Se realizó abordaje lateral izquierdo en el 75% y se realizó por XLIF 3 niveles en el 55% (45% <3). En el 60% no hubo ningún cambio significativo y sin déficits neurológicos. En el 20% hubo descargas de alta frecuencia en EMG, sin otros cambios asociados, de los cuales 1 presentó dolor glúteo de nueva aparición. En el otro 20%, hubo cambios asociados, en 2 alteraciones en SSEP de safenos (ambos con molestias sensitivas y en uno debilidad de psoas) y en 2 cambios transitorios en TcMEP (no tuvieron secuelas motoras, pero uno tuvo molestias sensitivas) Una cirugía tuvo que interrumpirse por sangrado venoso y otro paciente falleció por perforación colónica. El 60% de los pacientes recibió el alta en <7 días. **Discusión/Conclusiones**: En nuestra serie, el 20% de los pacientes tuvo algún tipo de secuela neurológica siendo la MIO útil en la detección de pacientes en riesgo y siendo requerida por nuestros cirujanos para disminuir la morbilidad. La XLIF es bien tolerada por la mayoría de nuestros pacientes, pero puede asociarse a complicaciones perioperatorias graves.

**Monitorización Neurofisiológica Intraoperatoia (MNIO) en Cirugía Endoscópica**

Adriana Gómez Domínguez^1^, Raidili Cristina Mateo Montero^2^, Tomasz Zbigniew Rumin^1^, Alicia Collado Gonsálvez^3^

^1^Hospital Infanta Elena, 28342 Madrid, España

^2^Neurotoc, 08025 Barcelona, España

^3^Hospital Clínico San Carlos, 28040 Madrid, España

**Introducción y Objetivos**: La cirugía endoscópica lumbar es un procedimiento mínimamente invasivo que, aunque ofrece ventajas, conlleva riesgos de lesión neurológica, especialmente durante la colocación de la cánula y la manipulación de las raíces nerviosas. La MNIO puede minimizar estos riesgos al proporcionar información en tiempo real sobre la integridad de las estructuras neurales. **Material y Métodos**: Se presenta un estudio prospectivo de 65 procedimientos endoscópicos lumbares, en los que se realizó una MNIO multimodal y continúa (TcMEPs, PESS, PRMR, RBC, EMG de barrido libre y mapeo). Los pacientes se dividieron en dos grupos según el tipo de abordaje: 24 procedimientos transforaminales y 41 interlaminares. En los transforaminales, se realizó mapeo de la cánula para valorar su proximidad a estructuras neurales y ajustar la trayectoria si era necesario. En los interlaminares, se priorizó el control de las raíces sacras mediante técnicas de MNIO. **Resultados**: En los abordajes transforaminales, el umbral de mapeo varió entre 7 y 56 mA (media: 21,47 mA). En tres casos se observaron umbrales superiores a 50 mA, en los cuales los registros basales (TcMEPs y PRMR) ya mostraban disfunción de la raíz afectada, lo que sugiere que valores elevados de mapeo pueden reflejar alteraciones funcionales previas. En los interlaminares, se registraron descargas transitorias en el EMG de barrido libre, lo que permitió ajustar la técnica quirúrgica y evitar daño neurológico. En tres casos, se observó un descenso temporal de TcMEPs sin déficit motor postoperatorio. **Discusión/Conclusiones**: La MNIO multimodal va ser una herramienta útil en la cirugía endoscópica lumbar, proporcionando información en tiempo real para la identificación de riesgos neurológicos y facilitando la toma de decisiones intraoperatorias. Su aplicación debe ajustarse a los riesgos específicos de cada tipo de abordaje: el mapeo de la cánula es crucial en los procedimientos transforaminales, mientras que el control de las raíces sacras es esencial en los interlaminares.

**Monitoreo Neurofisiológico Multimodal en Descompresión de Fosa Posterior sin Eventos de Alarma**

José Ramón Rosales Gutiérrez^1^, Gabriel Alfonso Carranco Toledo^1^, Héctor Humberto Gómez Acevedo^2^

^1^Centro De Rehabilitación Fisica Carranco SC, 06700 Ciudad de México, México

^2^Hospital Angeles Acoxpa, 14308 Ciudad de México, México

**Introducción y Objetivos**: El monitoreo neurofisiológico intraoperatorio (MIO) se ha consolidado como una herramienta fundamental para la prevención de lesiones neurológicas durante cirugías craneales complejas. El objetivo de este trabajo es presentar el caso clínico de una paciente sometida a descompresión de fosa posterior con vigilancia neurofisiológica multimodal, destacando su utilidad para preservar la integridad funcional de las vías motoras, sensitivas y de pares craneales. **Material y Métodos**: Se realizó MIO durante la intervención quirúrgica de descompresión de fosa posterior bajo anestesia TIVA. Se emplearon los siguientes procedimientos: PESS: estimulación de nervios tibial posterior y cubital bilateral, con registro cortical (C3, C4, Cz, Fz); PEM: estimulación transcraneal (C1/C2, C3/C4) con registro en músculos Abudctor del quinto dedo y gemelo medial; EMG continuo: registro en músculos periféricos (trapecio, deltoides, bíceps, triceps y gastrocnemio) y musculatura laríngea mediante electrodo endotraqueal Dragonfly. Se realizaron registros basales, intraoperatorios continuos y finales, con verificación de impedancias <5 kΩ. **Resultados**: PESS: sin cambios significativos en amplitudes ni latencias en comparación con los registros basales (no se superó el umbral de alarma <50% de disminución de amplitud o <10% de incremento en latencia); PEM: respuesta bioeléctrica mantenida en todos los miotomos evaluados, sin pérdida de señal ni modificaciones críticas intraoperatorias; EMG continua: sin registros de irritación nerviosa, con silencio eléctrico basal y estabilidad durante todo el procedimiento. **Discusión/Conclusiones**: El uso de monitoreo multimodal permitió la vigilancia funcional en tiempo real de las vías motoras, sensitivas y núcleos de pares craneales sin hallazgos de alarma intraoperatoria. La correlación entre hallazgos electrofisiológicos estables y el buen desenlace clínico postoperatorio refuerza el valor del MIO como herramienta preventiva en neurocirugía de base de cráneo y de la unión craneocervical.

**Monitorización Neurofisiológica Intraoperatoria en la Cirugía de Descompresión Microvascular por Espasmo Hemifacial**

Guillermo Martín Palomeque^1^, Olga Fedirchyk Tymchuk^1^, Liliana González Rodríguez^1^, Lidia Cabañes Martínez^1^, Ignacio Regidor Bailly-Bailliere^1^

^1^Hospital Universitario Ramon y Cajal, 28034 Madrid, España

**Introducción y Objetivos**: La descompresión microvascular es una de las opciones terapeúticas en los casos de espamo hemifacial no paralítico y de primera elección en aquellos en los que se evidencia un vaso comprimiendo al nervio facial. Este procedimiento busca aliviar la presión sobre el nervio facial al separar el vaso que lo comprime. Es un procedimiento seguro y efectivo aunque no exento de riesgos. El déficit neurológico postoperatorio más frecuente es la disfunción del VIII par. El objetivo de este trabajo es evaluar el valor de la monitorización neurofisiológica intraoperatoria (MNIO) en estos casos, no solo para evitar complicaciones neurológicas postoperatorias sino para tratar de asesorar sobre la eficacia del procedimiento quirúrgico. **Material y Métodos**: Se analizó la MNIO realizada en 9 casos de descompresión microvascular por espasmo hemifacial en el HU Ramon y Cajal durante los últimos 4 años. Se revisaron las técnicas neurofisiológicas utilizadas que además de las convencionales incluían el lateral spread response (LSR) y una modificación del blink reflex (BR). Se trató de establecer cual de ellas es la mas sensible a la hora de evitar complicaciones neurológicas y cuál la más útil para asesorar sobre la eficacia del procedimiento. **Resultados**: 5 de 9 pacientes tuvieron una afectación del VIII par aunque solo en un caso fue permanente. Sobre la eficacia de la descompresión, el LSR y el BR modificado se comportaron de una forma similar y todos aquellos pacientes en los que desaparecieron tanto el LSR como la respuesta anormal del BR (9/9) se despertaron sin espasmo. 7 pacientes están asintomáticos y en 2 el espasmo reapareció a las 24 horas. En estos dos pacientes las respuestas anormales de LSR y BR desaparecieron inmediatamente después de abrir la aracnoides y no tras separar el vaso ofensor. **Discusión/Conclusiones**: Es altamente recomendable la utilización de monitorización neurofisiológica intraoperatoria en estos casos, no solo para evitar complicaciones neurológicas sino para asesorar en la eficacia del procedimiento a corto plazo.

**Complicación Intraoperatoria Durante la Resección de un Glioma Insular**

Alba Sánchez Tudela^1^, Artem Kuptsov Kuptsov^1^, Cristina Gómez Revuelta^1^, Pedro Tomás Jerez García^1^, Pablo González López^1^, María Dolores Coves Piqueres^1^

^1^Hospital General Universitario Dr. Balmis, 03010 Alicante, España

**Introducción y Objetivos**: Los gliomas insulares se consideraban inoperables pero con los avances tecnológicos actuales se persigue un equilibrio entre resección y preservación funcional. Presentamos un caso donde un vasoespasmo en la arteria M2 causó pérdida de potenciales evocados motores (PEM) corticales justificando el uso de monitorización intraoperatoria neurofisiológica (MION) para detectar y tratar estas complicaciones intraoperatorias. **Material y Métodos**: Paciente de 43 años que ingresa por crisis tónico-clónica sin déficits neurológicos. Se diagnostica de lesión ocupante de espacio sobre ínsula derecha en resonancia magnética (RM) programándose cirugía con MION multimodal. **Resultados**: Durante la resección tumoral subcortical se registran cambios significativos de las respuestas motoras evocadas por estimulación cortical directa (PEM corticales). Se descarta causa técnica/anestésica y se avisa al neurocirujano realizándose angiografía intraoperatoria que evidencia vasoespasmo en la arteria M2. Se aplican medidas de neuroprotección intracampo recuperándose progresivamente las respuestas motoras correspondientes. En el postoperatorio inmediato presenta hemiparesia leve transitoria con resección casi total en RM, siendo dado de alta sin déficits motores. **Discusión/Conclusiones**: La complejidad de los tumores insulares radica tanto en el abordaje quirúrgico como en la elevada vascularización de esta zona, que incrementa el riesgo de accidentes vasculares intraoperatorios. Es por ello que, la resección tumoral debe detenerse antes de comprometer arterias cerebrales profundas para evitar lesiones. Los PEM transcraneales están desaconsejados para evaluar la vía corticoespinal en patología supratentorial debido al riesgo de pasar inadvertida una lesión isquémica cortical. Este caso pone de manifiesto la importancia de realizar PEM corticales en este tipo de intervenciones, no sólo para diagnosticar un evento isquémico/lesivo sino también aplicar medidas de neuroprotección *in situ* a fin de evitar déficits motores en el postoperatorio.

**Valor de la Monitorización Neurofisiológica en las Lesiones Intramedulares. Una Serie de Casos**

Carla Porto Fernández^1^, Iria Lagoa Labrador^1^, Alberto Ulloa Meijide^1^, Beatriz Soria Soriano^1^, Valeria Fra Mosquera^1^, Carina Diéguez Varela^1^, Elena Lojo Lendoiro^1^

^1^Hospital Álvaro Cunqueiro, 36312 Vigo, España

**Introducción y Objetivos**: La resección quirúrgica continúa siendo el tratamiento más efectivo en la mayoría de las lesiones intramedulares, pero a pesar de los avances en las técnicas quirúrgicas, la resección conlleva riesgos de daño neurológico. Con esta revisión se pretende valorar el papel de la monitorización intraoperatoria (MNIO) en la resección de dichas lesiones y su correlación entre los cambios intraoperatorios y los resultados clínicos neurológicos postoperatorios. **Material y Métodos**: Estudio retrospectivo de pacientes que se sometieron a una resección y/o biopsia quirúrgica de lesiones intramedulares entre 2017 y 2025 en nuestro centro. Las variables estudiadas fueron tamaño del tumor, nivel medular, técnicas neurofisiológicas empleadas, alarmas intraoperatorias y estado neurológico en el postoperatorio inmediato, a los 6 meses y a los 2 años. Se realizó monitorización neurofisiológica en todas las cirugías y los cambios en la MNIO se establecieron en función de los criterios publicados por V. Deletis y F. Sala en 2006. **Resultados**: Los pacientes identificados fueron 14, presentando un rango de edad de entre 17 y 80 años. De todos ellos, 6 eran hombres y 8 eran mujeres. En 4 de ellos la biopsia intraoperatoria no mostró rasgos morfológicos de neoplasia por lo que se detuvo la resección; en los 9 restantes, la anatomía patológica de la lesión correspondía a un ependimoma y en 1 de ellos a un astrocitoma. En 7 de las intervenciones hubo alarmas intraoperatorias. En el seguimiento postquirúrgico, se ha visto que en los pacientes con cambios en las señales de la MNIO durante el mismo presentan mayores déficits neurológicos que aquellos en los que no los hubo. **Discusión/Conclusiones**: Importancia de la implementación de la MNIO en el reconocimiento precoz de daño neurológico y mejora de los resultados postquirúrgicos. Se pone en evidencia la utilidad de la MNIO en la preservación de la función neurológica y, por tanto, de la calidad de vida del paciente.

**Comparativa en Trasplante Pulmonar con y sin Monitorización de Nervio Frénico y Estudio pre y Postquirúrgico**

Lucas Rafael Pérez-Cuesta Llaneras^1^, Virginia Russu^1^, Josué Alberto Montiel Salazar^1^, Patricia Eliana Sainz Vecino^1^, Edwin Ebrat Mancilla^1^, Luis Fernando López Pájaro^1^, Alberto Ignacio Pérez de Vargas Martínez^1^

^1^Hospital Universitario Puerta de Hierro Majadahonda, 28222 Madrid, España

**Introducción y Objetivos**: La disfunción del nervio frénico es una complicación potencial en pacientes sometidos a trasplante pulmonar, con implicaciones importantes en la función diafragmática y la recuperación respiratoria. Nuestro objetivo es valorar si los pacientes sometidos a monitorización intraoperatoria del nervio frénico tienen mejores resultados clínicos y neurofisiológicos. **Material y Métodos**: Durante el año 2023, se realizó estudio electromiográfico y de conducción nerviosa motora de nervio frénico pre y post trasplante pulmonar a 62 pacientes, de los cuales a 47 se les realizó monitorización de nervio frénico intraoperatoria, y a 15 se les realizó la cirugía sin monitorización. Posteriormente se recogen una serie de variables demográficas, clínicas y neurofisiológicas para comparar resultados entre ambos grupos. **Resultados**: Globalmente, los pacientes monitorizados mostraron mejores resultados en las diferentes variables estudiadas. De los pacientes monitorizados, el 38,3% precisó de traqueostomía durante el ingreso, en comparación con un 56,5% de los pacientes no monitorizados. Así mismo, hasta un 16% de los pacientes monitorizados tuvieron una estancia inferior a 4 días en la unidad de cuidados intensivos (media de estancia en UCI de 13,8 días), mientras que todos los pacientes no monitorizados tuvieron siempre una estancia mayor a 4 días (media de 23,5). En cuanto a estancia hospitalaria total, tuvieron una estancia menor a un mes (27 días), un 3,6% de los pacientes monitorizados (media de estancia hospitalaria 50,2) y todos los pacientes no monitorizados estuvieron más de un mes (media de 57,1). **Discusión/Conclusiones**: La monitorización intraoperatoria del nervio frénico no sólo es una técnica fácil, reproducible y sin aparentes complicaciones, sino que se relaciona a mejor resultado clínico y menos estancia hospitalaria, resultando en un beneficio para el paciente, limitando las posibles complicaciones de ingresos prolongados, y además resultaría un ahorro económico importante para la sanidad pública.

**Mapeo Cortical y Subcortical del Área Motora Suplementaria en Cirugía de Tumores Cerebrales**

Raidili Cristina Mateo Montero^1^, Gerardo Conesa Bertrán^2^, Marta Cicuendez López-Ocaña^2^, Cristóbal Perla y Perla^3^, Carlos Ascencio Cortez^3^, Mireia Illueca Moreno^3^, Estela Lladó Carbó^1^

^1^Neurotoc, HM Now Delfos, 08023 Barcelona, España

^2^Centro Médico Teknon, 08022 Barcelona, España

^3^HM Now Delfos, 08023 Barcelona, España

**Introducción y Objetivos**: La preservación del área motora suplementaria (AMS) es fundamental durante la resección de tumores cerebrales, dado su rol en el control motor, la fluidez verbal y las funciones ejecutivas. Su afectación puede generar déficits transitorios o permanentes que impactan la recuperación funcional y cognitiva del paciente. Aunque el AM puede identificarse con precisión mediante mapeo intraoperatorio, la AMS presenta mayor variabilidad anatómica, y el mapeo subcortical bajo anestesia general ha sido poco descrito. El objetivo de este estudio es describir una técnica reproducible para el mapeo cortical y subcortical de la AMS en pacientes dormidos. **Material y Métodos**: Se realizó monitorización neurofisiológica multimodal en 6 pacientes con tumores en regiones elocuentes, incluyendo tcmEP, SSEP, EEG, EMG, mapeo cortical y subcortical con estimulación monopolar (tren de 5 estímulos, 3–7 pulsos, 500 Hz, hasta 25 mA). Las respuestas se registraron en musculatura colateral e ipsilaterales. **Resultados**: En todos los pacientes se registró mapeo cortical directo de área motora y mapeo subcortical de área motora (registrando en musculatura colateral) y mapeo subcortical de área motora suplementaria (registrando en musculatura contralateral) y subcortical de AMS (registrando en la musculatura contraleteral e ipsilateral simultáneamente). Los pacientes despertaron un cuadro clínico transitorio (duró varias semana) de aquinesia del hemicuerpo contralateral al hemisferio cerebral lesionado y mutismo. **Discusión/Conclusiones**: El mapeo permite la identificación de la AMS reproducible y sus conexiones en pacientes bajo anestesia general, contribuyendo a su preservación. Dada su integración en redes funcionales fronto-subcorticales bilaterales, la identificación intraoperatoria de estas áreas resulta crucial para minimizar déficits y optimizar la recuperación funcional. La AMS y pre-AMS deben considerarse estructuras funcionales críticas en la planificación y ejecución quirúrgica de tumores cercanos a la corteza rolándica.

**Afectación De Los PEATC Según La Distinta Localización De Los Schwanomas Vestibulares**

Vicenç Pascual Rubio^1^, Adrián Solé Mialaret^1^, Vanesa Rius Costa^1^, Montserrat Roselló Foguet^1^, Leticia Carballo Lahoz^2^, Jorge Luis Meran Gil^2^, Dennis Harold Céspedes Torrez^2^

^1^Hospital Universitario Sant Joan de Reus, 43204 Tarragona, España

^2^Hospital Universitario de Tarragona Juan XXIII, 43005 Tarragona, España

**Introducción y Objetivos**: El Swhannoma vestibular (SV) se origina a nivel del octavo par craneal con preferencia por el nervio vestibular superior. Su origen generalmente se suele ubicar en la zona de Obersteiner-Redlich, en las proximidades del canal auditivo interno. El crecimiento del SV suele ser impredecible. La extensión intracanalicular provoca, en los estadios inciales, un daño axonal por compresión y una afectación de la arteria coclear. En cambio, el crecimiento extracanalicular produce, en estadios más avanzados con un mayor volumen tumoral, un daño por compresión del nervio e incluso del tronco cerebral. Objetivo: Determinar si los potenciales evocados auditivos de tronco cerebral (PEATC) pueden predecir la localización (intra o extracanalicular) de los SV. **Material y Métodos**: Estudio observacional y retrospectivo analizando los PEATC de 34 pacientes con SV unilateral, diagnosticados por resonancia magnética nuclear (RMN) y clasificados en dos grupos: intra y extracanaliculares. Se valoran las latencias y amplitudes de los PEATC. **Resultados**: Sospecharemos un SV extracanalicular cuando: el intervalo I–III sea mayor de 2,7 ms; cuando el intervalo I–V se encuentre por encima de 4,9 ms; y cuando la latencia de la onda V supere los 6,7 ms. Por el contrario, la amplitud de la onda I y el intervalo III–V no presentan resultados homogéneos en ambos grupos. Las limitaciones más importantes del estudio son: la necesidad de ampliar la muestra, la incorporación de información trascendente como el tiempo transcurrido entre la realización del PEATC y la RM; y el no tener en cuenta la posibilidad de SV con componente intra y extracanalicular. **Discusión/Conclusiones**: Los distintos patrones de afectación de los PEATCs podrían guardar relación con la localización intra o extracanalicular de los SV.

**Análisis de una Nueva Herramienta Para la Localización de Puntos en la Superficie Craneal en Pruebas Neurofisiológicas**

Vicenç Pascual Rubio^1^, Agnès Rigo Vidal^2^, Gisel Fabiola Montoya^3^, Montse Roselló Foguet^3^, Jordi Julvez Calvo^4^, Rosa Pàmies Vilà^5^, Albert Fabregat Sanjan^6^

^1^Hospital Universitario Sant Joan de Reus, 43204 Tarragona, España

^2^Hospital Univeritario Sant Joan De Dèu, 08950 Esplugues de Llobregat, Barcelona, España

^3^Hospital Universitari Sant Joan de Reus, 43204 Tarragona, España

^4^Institut d’Investigació Sanitària Pere Virgili, 43204 Tarragona, España

^5^Universitat Politècnica de Catalunya, 08034 Barcelona, España

^6^Universitat Rovira i Virgili, 43002 Tarragona, España

**Introducción y Objetivos**: La aparición de técnicas como la estimulación cerebral no invasiva o el estudio cuantitativo de la señal bioeléctrica cerebral hace necesario incrementar la precisión del posicionamiento de electrodos en la superficie craneal. El sistema convencional con cinta métrica es demasiado dependiente del personal sanitario y puede implicar errores de posicionado. Los sistemas avanzados como la neuro navegación o el escaneado 3D son complejos y tienen un coste elevado. Por estos motivos, se ha diseñado un dispositivo, EPlacement, para proporcionar, de forma rápida, sencilla, guiada y precisa, la localización de puntos craneales para las distintas pruebas neurofisiológicas. El objetivo del estudio es comparar el posicionamiento de electrodos convencional con EPlacement. **Material y Métodos**: Se realiza un estudio donde 10 profesionales sanitarios llevan a cabo 90 marcajes en 30 cabezas de maniquíes y en voluntarios. Cada uno realiza diferentes marcajes utilizando la cinta métrica y dos versiones de EPlacement, para 3 montajes de potenciales evocados: de 3, 5 y 9 puntos craneales. Se compara el tiempo utilizado, la precisión en la localización y la usabilidad de cada una de las técnicas de marcado. **Resultados**: EPlacement mejora la precisión del marcado en comparación con el método convencional. En las posiciones que se encuentran en los planos Ns-In y LPA-RPA, el dispositivo disminuye el error de posicionado, de media, en un 35% respecto la cinta métrica. En posiciones que no se encuentran en los planos Ns-In y LPA-RPA, esta mejora es del 75%. Además, reduce el tiempo de preparación por paciente en un 50% si se utiliza el método 10/20, y un 25% cuando se utilizan métodos aproximados. La encuesta de opinión demuestra que el personal sanitario está satisfecho con EPlacement y muestra una clara disposición a utilizar el nuevo dispositivo en la práctica clínica habitual. **Discusión/Conclusiones**: EPlacement reduce el tiempo de marcado, mejora significativamente la precisión en el posicionado de electrodos y facilita la práctica clínica.

**Neuropatía Auditiva: A Propósito de un Caso**

Shijia Li Chen^1^, Fernanda Romero Puertas^1^, Isabel Dolz Zaera^1^, Jorge de Francisco Moure^1^

^1^Hospital Universitario Miguel Servet, 50009 Zaragoza, España

**Introducción y Objetivos**: La neuropatía auditiva es un trastorno caracterizado por una percepción alterada del habla (hipoacusia retrococlear), a pesar de umbrales auditivos relativamente conservados. Los pacientes suelen presentar función preservada de las células ciliadas externas, evidenciada por otoemisiones acústicas (OEA) y/o microfónicos cocleares (MC) presentes, mientras que los potenciales evocados auditivos de tronco cerebral (PEATC) están ausentes o gravemente alterados, y los potenciales evocados auditivos de estado estable (PEAEE) pueden estar conservados. **Material y Métodos**: Se presenta el caso de una lactante con hipoacusia neonatal, nacida muy pretérmino (25 semanas, 600 g), que requirió ingreso en UCI neonatal con oxigenoterapia hasta los 67 días de vida. Ante ausencia de respuesta en las OEA, se realizaron estudios neurofisiológicos, incluyendo PEATC, PEAEE y MC. **Resultados**: Los PEATC realizados a las 38 semanas de edad corregida evidenciaron una hipoacusia profunda bilateral, identificándose únicamente la onda I en oído derecho. En un control tres meses después, los hallazgos se mantuvieron sin cambios. Estos resultados llevaron a otorrinolaringología a optar por la adaptación de audífonos y presentar el caso en la mesa de implantes cocleares, así como ampliación del estudio con PEATC, PEATEE y MC bajo sedación entre los 10 y 16 meses de edad corregida. En los PEATC, se siguió identificándose solo la onda I en oído derecho; los PEATEE mostraron umbrales superiores a 90 dB en todas las frecuencias; los MC estaban presentes en ambos oídos. Finalmente, se desestimó el implante coclear ante resultados compatibles con neuropatía auditiva. **Discusión/Conclusiones**: Las pruebas neurofisiológicas fueron cruciales para establecer el diagnóstico de neuropatía auditiva y orientar el manejo terapéutico. La paciente evolucionó favorablemente con el uso de audífonos, evidenciado en audiometrías conductuales posteriores con umbrales a 35 dB, confirmando la eficacia de la intervención no quirúrgica.

**Estudio Electrofisiológico de la Visión en Paciente con Bestrofinopatia Autosomica Recesiva**

Guillermo Ceide García^1^, Paloma Balugo Bengoechea^1^, Adela Fraile Pereda^1^, Lorena Iglesias Alonso^1^, Alejandro Melcón Villalibre^1^, Gloria Moreno García^1^, Andrea Mier San Juan^1^

^1^Hospital Universitario Clínico San Carlos, 28040 Madrid, España

**Introducción y Objetivos**: Variantes patogénicas en el gen *BEST1* se han asociado a distrofia hereditarias de la retina. La bestrofinopatía recesiva es una forma de distrofia retiniana macular heredada de forma autosómica recesiva con muy baja prevalencia, que a diferencia de la enfermedad de Best clásica, no presenta la típica imagen “en yema de huevo” en el fondo de ojo y asocia otras expresiones clínicas como mayor riesgo de glaucoma, por lo que su diagnóstico es complejo. **Material y Métodos**: Se describe el caso de una paciente de 44 años con baja agudeza visual, diagnosticada a los 20 años de glaucoma de ángulo abierto. Clínicamente la paciente progresa a un cuadro de nictalopía, dificultad para la visión de cerca, fotofobia y fotopsias, con fondo de ojo y autoflorescencia que evidencian una afectación macular severa, por lo que se cursan pruebas neurofisiológicas y estudio genético sospechando una distrofia hereditaria de retina. **Resultados**: En el ERGd se obtuvo un descenso moderado de amplitud b en respuesta combinada, una disminución severa del índice de Arden en el EOG, posteriores controles con hallazgos similares, así como una respuesta fotópica negativa (PhNR) patológica. Estos hallazgos son compatibles con una alteración severa del epitelio pigmentario y de las células ganglionares de la retina, sin alteración de fotorreceptores. **Discusión/Conclusiones**: El estudio genético confirmó una bestrofinopatía autosómica recesiva. Esta entidad presenta un espectro clínico más extenso y severo que su variante dominante, por lo que los hallazgos electrofisiológicos pueden verse alterados por solapamiento de entidades (glaucoma +/- distrofia retiniana). La paciente a pesar del tratamiento evolucionó con pérdida visual total en el ojo izquierdo por glaucoma terminal y baja agudeza visual en el derecho. Se trata de una patología rara, que suele acarrear retrasos diagnósticos importantes por lo que el estudio de electrofisiología de la visión es fundamental para una orientación diagnostica temprana y mejor abordaje pronóstico de los pacientes.

**Eficacia de EMTr en el Manejo del Dolor de Miembro Fantasma: Experiencia Clínica en una Serie de Casos**

Miguel Cobo Moreno^1^

^1^Hospital Universitario Virgen de las Nieves, 18012 Granada, España

**Introducción y Objetivos**: El dolor de miembro fantasma (DMF) representa un desafío terapéutico significativo, con frecuente resistencia a abordajes convencionales. Presentamos los resultados a largo plazo de estimulación magnética transcraneal repetitiva (EMTr) en una cohorte heterogénea del Hospital Universitario de Turku. **Material y Métodos**: Realizamos un análisis retrospectivo de 4 pacientes (15–83 años) con amputación de miembro superior y DMF refractario, tratados con protocolos personalizados de EMTr (10 Hz sobre corteza motora/somestésica contralateral) entre 2017–2024. Se evaluó evolución clínica mediante escalas numéricas de dolor (NRS), reducción de medicación analgésica, impresión clínica global (ICG) y parámetros funcionales durante seguimiento prolongado (3–7 años). **Resultados**: La intervención demostró eficacia sostenida en todos los casos, con reducciones significativas en intensidad dolorosa (50–100% en NRS), destacando la resolución completa en un paciente geriátrico (NRS 8→0), mejoría del 75% en un caso pediátrico (NRS 8→2) y efectividad en un caso complejo con neuropatía mediante terapia combinada con estimulación transcraneal por corriente directa (NRS 8→4). Se objetivó mejoría funcional global con aumento de horas de sueño (+2.5 h/día), reducción de crisis dolorosas (68%) y disminución/retirada de opioides en todos los casos. **Discusión/Conclusiones**: La EMTr constituye una alternativa eficaz en el manejo del DMF refractario, con resultados sostenidos a largo plazo. La optimización mediante protocolos individualizados y abordajes multimodales amplía sus perspectivas terapéuticas, ofreciendo una valiosa opción para reducir la carga farmacológica y mejorar la calidad de vida.

**Experiencia de una Consulta de Estimulación Magnética Transcraneal en Hospital Terciario Madrileño**

Diego Camargo Niño^1^, Julio Prieto Montalvo^1^

^1^Hospital General Universitario Gregorio Marañón, 28007 Madrid, España

**Introducción y Objetivos**: La estimulación magnética transcraneal repetitiva (rTMS) se presenta como técnica no invasiva útil en el tratamiento de múltiples trastornos neurológicos y psiquiátricos. Pese a su creciente uso, aún son escasos los centros que disponen de una consulta estructurada en el ámbito hospitalario público. Objetivo: Describir la estructura, funcionamiento y organización de una consulta hospitalaria de TMS, con el fin de aportar una guía práctica para su implementación en otros centros. **Material y Métodos**: Estudio descriptivo retrospectivo basado en la consulta de rTMS del Hospital General Universitario Gregorio Marañón, en funcionamiento desde 2015. Se describen los recursos materiales y humanos, el circuito asistencial, los protocolos aplicados, las áreas de estimulación utilizadas y los perfiles clínicos de los pacientes atendidos. **Resultados**: La consulta cuenta con dos puestos de tratamiento activos, equipados con dispositivos para rTMS. Las áreas tratadas y los protocolos se seleccionan individualmente según el diagnóstico y el objetivo terapéutico. El equipo está integrado por un médico neurofisiólogo responsable de la valoración inicial, determinación del umbral motor y prescripción del protocolo; y personal de enfermería encargado de la aplicación y seguimiento diario. Se han atendido más de 200 pacientes por patologías como depresión, dolor neuropático, ictus, afasia, entre otros. La planificación incluye escalas validadas antes y después del tratamiento. El modelo de consulta se basa en derivaciones directas desde distintas especialidades, con priorización según complejidad y urgencia. **Discusión/Conclusiones**: La experiencia acumulada ha permitido consolidar una consulta versátil y eficaz en el contexto hospitalario público. La combinación de protocolos personalizados, supervisión y seguimiento estructurado permite abordar un amplio abanico de indicaciones con garantías de seguridad y calidad asistencial. Esta experiencia puede servir como referencia para la creación de unidades similares en otros centros.

**Optimizar Desde el Primer Contacto: Criterios START/STOPP y Desprescripción en la Primera Visita de la Unidad de Sueño**

Octavio Jiménez Vega^1^, Inmaculada Rodríguez Ulecia^1^, Amanda Labrador^1^, Giada Buzzacchera^2^, Nayra Cabrera González^1^, José Juan Rodríguez Betancor^1^, Ayoze Nauzet González Hernández^1^

^1^Hospital Universitario de Gran Canaria Doctor Negrín, 35010 Las Palmas de Gran Canaria, España

^2^Consejería de Sanidad del Servicio Canario de Salud, 35071 Las Palmas de Gran Canaria, España

**Introducción y Objetivos**: La primera visita en la Unidad de Sueño (US) es clave para detectar la poliprescripción, ajustar tratamientos inadecuados y promover terapias no farmacológicas, especialmente en el insomnio. La poliprescripción dificulta nuestra práctica y margen clínico. El uso de criterios STOPP (uso desaconsejado)/START (buena práctica) permite guiar decisiones racionales. Este Audit analiza la carga farmacológica y estrategias de desprescripción en primeras visitas de nuestra unidad. **Material y Métodos**: Se realizó un estudio observacional tipo Audit analizando 100 primeras visitas de 2025 en la US de Neurofisiología. Se estableció un consensus de criterios STOPP/START específicos para las distintas patologías aferentes. Se recogieron datos sociodemográficos, diagnósticos, número y tipo de fármacos, hábitos tóxicos, medidas de higiene del sueño, efectos adversos e interacciones. Se analizaron también las especialidades derivantes, la realización de Polisomnografías (PSG) y la presencia de red flags. **Resultados**: La edad media fue de 54,1 años, con distribución equilibrada por sexo. Los pacientes tomaban una media de 6,7 fármacos, destacando hipnóticos (57%), analgésicos (53%) y antidepresivos (47%). El 70% presentaban posibilidad de desprescripción y el 59% cumplían criterios STOPP (8,2 vs 4,6; *p* < 0,001). El 65% recibió una intervención adecuada según criterios START. Las derivaciones más frecuentes fueron desde Neurología y Neumología. El 41% realizó PSG y un 46% tenía red flags de otros ámbitos. **Discusión/Conclusiones**: La primera visita en la Unidad de Sueño permite racionalizar tratamientos desde el inicio. El uso sistemático de criterios START/STOPP ayuda a detectar prescripciones inapropiadas y fomentar intervenciones adecuadas, integrando esta evaluación en la práctica clínica habitual. Es clave mejorar la colaboración con Atención Primaria y Salud Mental para derivaciones más precoces y establecer estrategias prescriptivas comunes, agilizando la realización de las PSGs y priorizar el uso racional de fármacos y medidas no farmacológicas.

**Trastorno de la Alimentación Relacionado con el Sueño (SRED), o Síndrome de la Cena Durante el Sueño**

Alexandra Margarita Navarrete Loza^1^, Walkiria Soto Cruz^1^, José Luis Relova Quinteiro^1^

^1^Hospital Clínico Universitario de Santiago de Compostela, 15706 Santiago de Compostela, A Coruña, España

**Introducción y Objetivos**: El SRED es una parasomnia NREM, caracterizada por episodios involuntarios de ingesta nocturna de alimentos, con combinaciones extrañas e incluso no comestibles, amnesia o memoria total/parcial de los episodios, y consecuencias derivadas para la salud. Hay que distinguirlo del síndrome de ingesta nocturna (NES), de S.Kleine-Levin o Klûver Bucy. El objetivo es analizar las características del SRED en contraste a otros posibles trastornos de alimentación nocturna. **Material y Métodos**: Historia clínica, PSG y revisión bibliográfica. **Resultados**: Mujer de 69 años, hipertensión, obesidad, dislipemia, fumadora. Desde 2010 tras la 1º hora de sueño, presenta episodios diarios de ingesta nocturna, 3–4 por noche, alguno con testigos, durante los que toma alimentación variada (gominolas, galletas, conservas, prepara café, etc.). Es consciente de ir a comer, no puede reprimirlo y la memoria es total o parcial. Despierta con comida en la boca o en la cama y afectación emocional. Riesgo de SAOS intermedio (STOP-BANG 4), menor calidad del sueño (T.Pittsburg 4). Sin otras características asociadas. PSG con sueño fragmentado, tiene 38 eventos respiratorios obstructivos (26 apneas, 12 hipopneas), por tanto, IAH 5,6/h con SaO_2_ mínima 79%. Además 271 movimientos periódicos de piernas (MPP) con índice 40,1/h. Sin episodios de ingesta observados. **Discusión/Conclusiones**: La paciente cuenta con los criterios ICSD-3 de SRED ya que sus episodios son involuntarios, sin hambre, a veces con memoria parcial; además, aunque no refiere consumir tóxicos, hay consecuencias para su salud: obesidad, anorexia matutina, riesgo de asfixia y quemaduras. Esto ayuda a distinguirlo de un NES, aunque es posible que compartan bases fisiopatológicas. No obstante, dado que tiene un SAOS leve y MPP moderado, su SRED es comórbido-secundario. El tratamiento inicial es corregir los mismos con la posibilidad de terapia cognitivo-conductual. Cabe mencionar que la PSG sin episodios no excluye el SRED, y que esta parasomnia podría ser un continuo con el grupo de trastornos del arousal.

**Estudio de Narcolepsia Tipo 1 en el Hospital Virgen del Rocío: Análisis Clínico y Neurofisiológico del Sueño**

Alicia Silva Cátedra^1^, Patricia Orive Rodríguez^1^, Paolo Porcacchia^1^, Francisco José Palomar Simón^1^, Rocío Vázquez Rodríguez^1^

^1^Hospital Universitario Virgen del Rocío, 41013 Sevilla, España

**Introducción y Objetivos**: Los trastornos del sueño son patologías infradiagnosticadas que afectan significativamente a la vida. Este estudio analiza pacientes diagnosticados de narcolepsia tipo 1, evaluando variables clínicas y neurofisiológicas en el Hospital Virgen del Rocío de Sevilla. **Material y Métodos**: Se seleccionaron 23 pacientes, sus datos clínicos y diagnósticos relacionados con polisomnografía (PSG) y prueba de latencia múltiple (TLM). **Resultados**: El 54% fueron hombres. La puntuación media en la Escala Epworth fue de 15,8, lo que refleja una somnolencia diurna significativa. La edad media de inicio de síntomas fue 19,5 años, siendo en la literatura de 25. La edad promedio de diagnóstico 27 años. Este retraso diagnóstico de 7,5 años fue menor que el presentado en otras publicaciones. La latencia media de sueño en el PSG fue de 4,9 minutos y en el TLMS de 1,8 minutos. Se obtuvieron resultados estadísticamente significativos: destacó una relación inversa entre la latencia de sueño en PSG y TLM con respecto a la frecuencia de episodios REM, indicando que a menor latencia de sueño en ambas pruebas, mayor número de episodios REM aparecían. Se obtuvo una menor latencia para alcanzar la fase REM en TLM en pacientes de menor edad. Sin embargo, fueron los más jóvenes los que experimentaron mayor dificultad para quedarse dormidos y alcanzar REM en PSG. Por último, un mayor índice de movimientos de miembros se asocia a un mayor número de despertares. **Discusión/Conclusiones**: Estos datos subrayan la importancia del diagnóstico precoz de narcolepsia tipo 1. Se observaron tiempos de latencia del sueño en PSG y TLM menores que los descritos en la literatura, que a su vez tenía relación con la aparición de episodios REM precoces. Los niños entran en fase REM en TLM antes que en PSG.

**A Propósito De Un Caso: Trastorno de Movimientos Rítmicos Durante el Sueño de Debut en la Edad Adulta**

Edgar Fernández Díaz^1^, Ana Isabel Fernández Bedoya^1^, Ana Martínez Zuluaga^1^, Ignacio José Martos Soto^1^, Silvia Taramundi Argüeso^1^

^1^Hospital Universitario de Cruces, Bilbao, España, 48903 Barakaldo, Bizkaia, País Vasco, España

**Introducción y Objetivos**: Los movimientos rítmicos durante el sueño se relacionan típicamente con la edad pediátrica y son considerados de naturaleza benigna, descritos como unas conductas motoras repetitivas, estereotipadas y rítmicas autolimitadas durante la infancia temprana, de predominio durante las transiciones vigilia-sueño. Si se produce una disrupción del descanso nocturno y/o un impacto significativo en la calidad de vida diurna, hablaríamos de un trastorno de movimientos rítmicos durante el sueño. El objetivo fundamental de esta comunicación es describir un caso atípico de trastorno de movimientos rítmicos durante el sueño de inicio en la edad adulta, pues se han descrito pocos casos de estas características en la literatura actual. **Material y Métodos**: Paciente de 55 años, sin AP previos de alteraciones del sueño, derivada por sospecha de hypnic jerks intensificados de debut hace 1 año, sin mejoría tras un intento terapéutico. No otros trastornos del sueño en la infancia. Se citó a la paciente para una consulta y posterior realización de video-Polisomnografía en el laboratorio de sueño. **Resultados**: Tras completar el estudio con v-PSG se orientó como un trastorno de movimientos rítmicos durante el sueño. No se identificaron otros trastornos de sueño concomitantes (como AOS o SPI, descritos en alguna de las publicaciones en población adulta). Al describir los movimientos, la paciente tampoco es plenamente consciente de los mismos, solo de una sensación de sobresalto ocasional a lo largo de la noche. Sí refiere una repercusión significativa en su calidad de vida, con síntomas diurnos limitantes derivados de un mal descanso nocturno. **Discusión/Conclusiones**: Al analizar este caso se pone de manifiesto el papel clave que juega el estudio con v-PSG a la hora de tipificar estos movimientos, su distribución a lo largo del sueño nocturno (para valorar la repercusión en el mismo) y a establecer un diagnóstico diferencial adecuado al tratarse de un trastorno muy poco frecuente en la edad adulta.

**¿Fracaso de los Antagonistas de las Orexinas?**

Andrea Aliaga Díaz^1^, Teresa Canet Sanz^1^, Alba Sánchez Tudela^1^, Lucía Soto Manzano^1^, Sara Giménez Roca^2^, Francis Sellés Galiana^1^, Sheila Picorelli Ruiz^1^

^1^Hospital General Universitario Dr. Balmis de Alicante, 03010 Alicante, España

^2^Hospital Clínico Universitario Virgen de la Arrixaca. 30120 Murcia, España

**Introducción y Objetivos**: El insomnio se caracteriza por una dificultad para la conciliación o mantenimiento del sueño, o por una mala calidad del mismo, que asocia una disfunción diurna. Una de las técnicas más empleadas en su estudio son las agendas de sueño. El principal inconveniente que plantean es que su evaluación, y con ello, los ajustes en el tratamiento del insomnio, se basan en los datos subjetivos percibidos por el paciente. Aunque la terapia cognitivo-conductual conforma la primera línea en el tratamiento del insomnio, también disponemos de un amplio abanico farmacológico, incluyendo los antagonistas de las orexinas. **Material y Métodos**: Presentamos el caso de una mujer de 56 años remitida a la Unidad de Sueño de nuestro hospital por sospecha de hipersomnolencia tras descartar AOS mediante poligrafía. Refiere inicio de la sintomatología hace 9 años, aquejando tiempo total de sueño insuficiente, con severa repercusión diurna (Epworth 17). Cumplimenta un primer DS que objetiva TTSE de 2,08 h, por lo que se inicia TCC asociando Daridorexant 50 mg. En la revisión del primer mes la paciente no refiere cambios significativos, persistiendo según su DS un TTSE de 2,12 h. Debido a la ausencia de mejoría con el tratamiento, se realiza estudio PSG manteniendo la misma dosis de Daridorexant. **Resultados**: La PSG muestra un TTS de 404,5 min (6,7 h), con una proporción de las fases de sueño dentro de la normalidad, a pesar de que la percepción de sueño de la paciente durante la noche del estudio fue de 3 h. **Discusión/Conclusiones**: Ante un paciente no respondedor a la terapia del insomnio debemos proponer estudio PSG para valorar otras patologías del sueño o, como en el caso presentado, una mala percepción de sueño. En estos casos, los DS no serán una herramienta útil para la monitorización del tratamiento, pudiendo ser de interés el estudio mediante actigrafía, no disponible en nuestro centro. Por otra parte, y concordando con lo descrito en la literatura, el tratamiento con Daridorexant no modificó la estructura del sueño en nuestra paciente.

**Discrepancia Entre Percepción Subjetiva de Sueño y Datos Objetivados Mediante Polisomnografía Nocturna (PSG-N)**

Raquel López-Carvajal Hijosa^1^, Gabriela Alejandra Naranjo Heredia^1^, Guillermo García-Rodríguez Lomas^1^, Fernando Neria Serrano^2^, Madeleyn Rodríguez Jiménez^1^, María Mercedes Picornell Darder^1^, Isabel del Río Bustillo^1^

^1^Hospital Universitario de Móstoles, 28935 Madrid, España

^2^Universidad Francisco de Vitoria, 28223 Madrid, España

**Introducción y Objetivos**: La percepción subjetiva de sueño explica como una persona evalúa y experimenta su propio sueño, independientemente de las medidas obtenidas en la PSG-N. Evaluar la concordancia entre la percepción subjetiva del sueño (encuesta) y los parámetros objetivados en la PSG-N. **Material y Métodos**: Se diseñó un estudio descriptivo observacional transversal en pacientes de una Unidad de Sueño evaluados mediante PSG-N. Un total de 158 pacientes fueron monitorizados, tras la cual se realizó una encuesta de sueño en la que se preguntó cuántas horas creen que han dormido durante el estudio y si ese sueño había sido reparador. Se recogieron las medidas habituales de eficacia y eficiencia del sueño durante la PSG-N. El coeficiente de correlación intraclase (ICC) y el coeficiente de Kappa de Cohen se emplearon para valorar la concordancia de estas medidas; entre lo percibido por el paciente y la realidad. **Resultados**: Un total de 158 participantes, con una edad media de 51 años (65,8% hombres). Los participantes tienden a subestimar significativamente la cantidad de horas que realmente duermen. La media de horas de sueño percibida es de 5 horas, mientras que la media real es de 6,4 horas (diferencia de 1,4 horas). El ICC obtenido es de 0,167 (IC 95%: 0,012–0,314), un valor muy bajo, lo que indica una baja concordancia entre las horas de sueño percibidas y las reales. En cuento a la eficacia, en la valoración objetiva se consideró eficaz si el valor era superior al 40%, mientras que, en la eficacia percibida, aquella que fue respondida como “sí” a la pregunta de si había tenido un sueño reparador. El Kappa de Cohen registrado fue de 0,072 (IC 95%: 0,085–0,228), un valor muy bajo que indica una falta de acuerdo entre la eficacia real y la percibida. **Discusión/Conclusiones**: La percepción individual subjetiva del sueño no es un buen predictor de la duración real ni de la calidad del mismo. Hay muy baja concordancia entre las horas de sueño y la calidad que las personas perciben comparando con los datos objetivos reales obtenidos en la PSG-N.

**DISE y PSG Simultánea: Optimizando la Terapéutica en AOS**

Claudia Perdomo Rubio^1^, Ainhoa Álvarez Ruiz de Larrinaga^1^, Jorge Andrés Núñez Rojas^1^, Erika Olea de La Fuente^1^, Carla Pía Martínez^1^, Alejandro José Horrillo^2^, Carlos Javier Egea Santaolalla^1^

^1^Hospital Universitario Araba, 01009 Vitoria‑Gasteiz, Álava, País Vasco, España

^2^Mayssonial

**Introducción y Objetivos**: La endoscopia del sueño inducido por fármacos (DISE) permite identificar niveles y patrones de colapso de la vía aérea superior en la apnea obstructiva del sueño (AOS). En nuestro hospital, el procedimiento lo realiza el Servicio de Otorrinolaringología, con Anestesia y de la Unidad Funcional del Sueño, con monitorización polisomnográfica (PSG) simultánea. Describir la experiencia con AOS sometidos a DISE con PSG simplificada simultánea. **Material y Métodos**: Estudio de cohorte retrospectivo que incluían pacientes diagnosticados de AOS relevante, que fueron sometidos a DISE desde octubre de 2023 hasta diciembre de 2024 en el Hospital Universitario Araba. Durante la DISE se registró monitorización simultánea con montaje de EEG con electrodos centrales, pulsioximetría, bandas torácicas y abdominales y posición. Se recogieron los datos antropométricos, índice de apnea-hipopnea (IAH), hallazgos endoscópicos (escala de VOTE) y actitud terapéutica adoptada posterior. **Resultados**: Se incluyeron 8 pacientes, 100% hombres, con una edad media de 52 ± 6,4 años, un índice de masa corporal medio de 29,3 ± 3,9 kg/m^2^, y un IAH medio de 30,5 ± 13,6 eventos/hora. La mitad presentaba AOS moderada y la otra mitad severa. En 7 pacientes la DISE permitió identificar niveles de colapso no evidentes en la exploración física previa. La monitorización simultánea facilitó la interpretación de los eventos respiratorios observados. En todos los casos se planteó una actitud terapéutica: DAM (18,8%), cirugía (50%), terapia posicional (18,8%) y medidas dietéticas (12,5%), independiente que siguieran o no con CPAP. **Discusión/Conclusiones**: La combinación de DISE con monitorización PSG simultánea aporta un valor diagnóstico adicional, ya que no solo permite una caracterización más precisa de la fisiopatología del AOS, sino que ayuda a visualizar objetivamente los eventos respiratorios, así como sus consecuencias en microdespertares y saturación. Todo ello, facilita la definición de la estrategia terapéutica más adecuada para cada paciente.

**Análisis de Patrones Lumínicos Hospitalarios Desde una Perspectiva Cronobiológica y su Impacto en la Calidad de Sueño**

Amanda Labrador Rodríguez^1^, Octavio Jiménez Vega^1^, Inmaculada Rodríguez Ulecia^1^, José Juan Rodríguez Betancor^1^, Víctor Melián Díaz^2^, David Cañizo García^1^, Ayoze Nauzet González Hernández^1^

^1^Hospital Universitario de Gran Canaria Doctor Negrín, 35010 Las Palmas de Gran Canaria, España

^2^Hospital Materno-Infantil de Canarias, 35016 Las Palmas de Gran Canaria, España

**Introducción y Objetivos**: Las condiciones lumínicas hospitalarias influyen de forma decisiva en la regulación circadiana del sueño, especialmente en pacientes vulnerables a disrupciones cronobiológicas. Las alteraciones de los ciclos de luz natural y artificial pueden favorecer insomnio, delirium y mayor uso de fármacos. Evaluar la adecuación lumínica desde una perspectiva neurofisiológica y del sueño aplicada permite orientar estrategias complementarias para mejorar el descanso. **Material y Métodos**: Se realizó un estudio prospectivo con mediciones de iluminancia (lux), temperatura de color (Kelvin) y espectro azul (DSP) en tres franjas horarias y distintos entornos hospitalarios (UMI, Urgencias, Neurología y áreas de trabajo), siguiendo criterios técnicos. Se evaluó el respeto al ritmo circadiano según la exposición a luz natural y la adecuación cronobiológica de la iluminación artificial. Además, se analizó retrospectivamente una cohorte de 100 pacientes de Neurología, comparando el uso de fármacos, alertas nocturnas y riesgo de delirium según su ubicación respecto a la fuente de luz. **Resultados**: El análisis técnico reveló que pocas zonas cumplían los criterios lumínicos nocturnos óptimos (<30 lux y <3000 K). Se observaron déficits de luz diurna y una sobreexposición nocturna a iluminación artificial por encima de lo fisiológicamente recomendable. Los pacientes con menor acceso a luz natural diurna presentaron un 30% más de alertas nocturnas y un leve aumento en el uso de sedantes frente a quienes estaban cerca de ventanas. **Discusión/Conclusiones**: La iluminación en áreas críticas y de hospitalización no respeta adecuadamente los ciclos luz-oscuridad, favoreciendo la cronodisrupción y posibles alteraciones del sueño y sobrecarga nocturna. La Unidad de Sueño, desde su enfoque neurofisiológico, puede asesorar un abordaje complementario y potencialmente eficaz basado en intervenciones estructurales y conductuales para mejorar la higiene circadiana, con un posible impacto en la reducción de delirium, trastornos del sueño y uso de fármacos.

**Estudio de Parámetros Respiratorios y Anatomoclínicos en la Malformación de Chiari Tipo 1 y 1,5 (MC) en Edad Infantil**

Douglas Alejandro Bolaños Purroy^1^, Alejandro Ferré Masó^1^, María José Jurado Luque^1^, Odile Romero Santo-Tomás^1^, Roser Elena Cambrodí Masip^1^, María Antonia Poca Pastor^1^, Joan Sahuquillo Barris^1^

^1^Hospital Universitari de la Vall D’Hebron, 08035 Barcelona, España

**Introducción y Objetivos**: Según series cortas de pacientes pediátricos, la MC puede asociarse a trastornos respiratorios del sueño (TRS), así como a alteraciones neurológicas estructurales y clínicas. El objetivo de este trabajo es realizar un estudio prospectivo en una cohorte amplia de pacientes pediátricos con MC, para describir las características respiratorias y clínicas. **Material y Métodos**: Estudio descriptivo prospectivo desde el año 2007 al 2023 donde se han recogido datos de 168 pacientes pediátricos diagnosticados con MC. Se incluyeron variables clínicas (edad, sexo, síntomas), presencia de siringomielia (SM), hidrocefalia (HC), pseudotumor cerebri (PTC) y odontoides retrocurva (OR). Los pacientes fueron evaluados mediante poligrafía cardiorrespiratoria o polisomnografía convencional, y se recogieron parámetros respiratorios del sueño. Se completaron los cuestionarios Sleep Disturbance Scale for Children de Bruni y Pediatric Quality of Life Inventory. **Resultados**: La media de edad fue de 9,74 años (rango 1–18), con predominio del sexo masculino (51,8%). El 63,7% presentaba Chiari tipo 1 y el 28,6% tipo 1,5. Un 30,4% presentó SM, un 14,3% HC, un 7,2% de PTC y 16,7% OR. Se identificaron, con un punto de corte de IAH de 3 e/h, un TRS en el 23,2% de los casos, con predominio de apneas centrales (58,9%). Existe una correlación positiva entre el descenso amigdalar y el IAH. Para un punto de corte de IAH 5 e/h, se asocia a mayor prevalencia de HC, PTC, OR y la presencia de síntomas. Los cuestionarios de Bruni y PedsQL mostraron puntuaciones elevadas en 31,8% de los casos. **Discusión/Conclusiones**: Los niños con MC presentan una alta prevalencia de TRS, mayoritariamente apneas centrales. Aquellos pacientes con un mayor descenso amigdalar, hidrocefalia, pseudotumor cerebri, odontoides retrocurva y síntomas, podrían presentar un mayor riesgo de TRS. El estudio del sueño debe considerarse en el abordaje integral de estos pacientes.

**Apnea del Sueño en Pacientes con Párpado Laxo Procedentes de la Consulta de Oculoplastia**

Cristina Moreno Jiménez^1^, Paula Arribas Pardo^1^, Mariano Aguilera Vergara^1^, Goran Josic^1^, Cielo González Mendoza^1^, Raúl Armas Zurita^1^, Fadi Hallal Peche^1^

^1^Hospital Central de la Defensa “Gómez Ulla”, 28047 Madrid, España

**Introducción y Objetivos**: El síndrome de párpado laxo se caracteriza por una hiperlaxitud palpebral y conjuntivitis papilar crónica, siendo una de sus características principales la fácil eversión de los párpados afectos, bien mediante tracción o bien espontáneamente durante el sueño. La apnea del sueño implica hipoxia tisular intermitente, estrés oxidativo y daño endotelial, aumentando con ello el riesgo cardiovascular a largo plazo, además de limitar la calidad de vida a corto plazo con síntomas como la hipersomnolencia diurna o el sueño no reparador. La prevalencia de la apnea del sueño puede ascender al 57,1% entre los pacientes con párpado laxo según la literatura. El objetivo del estudio fue comprobar si existe asociación entre el síndrome de párpado laxo y el diagnóstico de apnea del sueño en nuestra Unidad de Sueño. Descripción de los hallazgos polisomnográficos. **Material y Métodos**: Cribado de apnea del sueño en los pacientes con diagnóstico de síndrome de párpado laxo remitidos a nuestra Unidad de Sueño desde la consulta de Oculoplastia. **Resultados**: Se recopiló una muestra de 26 pacientes (21 varones y 5 mujeres, edades entre 55–87 años). 24 casos fueron positivos para apnea del sueño (92,3% de la muestra), refiriendo síntomas compatibles con clínica cardinal de apnea del sueño y al menos un factor de riesgo cardiovascular (obesidad, hipertensión arterial, dislipemia y/o diabetes mellitus). La eficiencia de sueño se encontraba reducida, con un valor medio del 70,5%, y una latencia de sueño media de 30,7 minutos. Arquitectura de sueño fragmentada, no se objetivó sueño REM en 5 casos, con un índice de número de cambios de fase por hora de sueño medio de 27, y tiempo de vigilia tras inicio del sueño de 77,8 minutos de media. **Discusión/Conclusiones**: La asociación entre párpado laxo y apnea del sueño se corroboró en nuestra muestra. El cribado de apnea del sueño en pacientes procedentes de la consulta de Oculoplastia reduciría la morbilidad de estos pacientes, mejorando su calidad de vida.

**“Epic Dreaming”: ¿Una Entidad Clínica Olvidada? Estudio Observacional de Cuatro Casos Desde la Neurofisiología del Sueño**

África Bueno García^1^, Christian Hernández Aranda^1^, Margely Abete Rivas^1^, José Miguel León Alonso-Cortés^1^, Sara Muniesa Lacasa^1^, Gonzalo Ferrer Ugidos^1^, María Martín Carretero^1^

^1^Hospital Clínico Universitario Valladolid, 47003 Valladolid, Castilla y León, España

**Introducción y Objetivos**: Explorar las características clínicas y neurofisiológicas del epic dreaming mediante cuatro casos con perfiles distintos, evaluando su relación con trastornos del sueño REM, NREM o psiquiátricos. **Material y Métodos**: Estudio descriptivo de cuatro pacientes remitidos por pesadillas, hipersomnia o sospecha de parasomnia, todos con reportes recurrentes de epic dreaming (sueños largos, complejos y exhaustivos). Se realizó polisomnografía (PSG) adaptada: una estándar y dos de 48 horas, analizando estructura del sueño, fases REM/NREM y eventos paroxísticos. **Resultados**: Paciente 1: Varón de 40 años con pesadillas y cansancio diurno. PSG normal, con leve disminución del sueño REM. Paciente 2: Mujer de 28 años con hipersomnolencia. PSG de 48 h sin alteraciones relevantes, sueño total de 10 h 38 min. Paciente 3: Mujer de 25 años con sospecha de parasomnia. PSG muestra episodios confusionales y patrón compatible con parasomnia NREM. Paciente 4: Varón de 33 años, con antecedentes de TOC y ánimo depresivo. PSG con parasomnia NREM y sueño REM fragmentado, con conductas motoras complejas, microdespertares, activación autonómica y recuerdo de pesadillas, sin pérdida de atonía. En todos los casos, los episodios de epic dreaming eran reiterados, intensos, con importante vividez y carga emocional, referidos como agotadores. **Discusión/Conclusiones**: El epic dreaming aparece en distintos perfiles clínicos, desde trastornos REM/NREM hasta hipersomnia y trastornos psiquiátricos, sin un patrón neurofisiológico común. Más que un trastorno estructural, refleja una experiencia subjetiva intensa ligada al procesamiento emocional onírico. Su impacto clínico (recuerdos vívidos, fatiga al despertar) suele subestimarse y podría indicar disfunción emocional o alteración en la integración de los sueños. Se recomienda su evaluación sistemática en pacientes con sueño no reparador o actividad onírica excesiva, incluyendo su anamnesis en unidades de sueño para mejorar diagnóstico y tratamiento.

**¿Epilepsia o Parasomnia? El valor de la PSG en el Diagnóstico de Episodios Nocturnos Complejos**

África Bueno García^1^, María Martín Carretero^1^, Christian Hernández Aranda^1^, Sara Muniesa Lacasa^1^, José Miguel León Alonso-Cortés^1^, Gonzalo Ferrer Ugidos^1^, Margely Abete Rivas^1^

^1^Hospital Clínico Universitario Valladolid, 47003 Valladolid, Castilla y León, España

**Introducción y Objetivos**: Describir un caso clínico con sintomatología nocturna compleja en el que la polisomnografía (PSG) permitió identificar simultáneamente hallazgos neurofisiológicos sugestivos de epilepsia temporal y de parasomnia NREM, subrayando su utilidad en el diagnóstico diferencial y en la orientación terapéutica. **Material y Métodos**: Se estudió a un paciente adulto con antecedentes de eventos nocturnos paroxísticos, sin hallazgos concluyentes en evaluaciones previas (EEG de rutina, RM cerebral y pruebas de sueño). Se realizó una polisomnografía de 48 horas con registro de vídeo y EEG ampliado con derivaciones temporales adicionales. Se analizaron parámetros cuantitativos de arquitectura del sueño, índice de despertares en ondas lentas durante N3, actividad epileptiforme interictal y presencia de eventos comportamentales anómalos. **Resultados**: El registro evidenció una arquitectura del sueño conservada, con ligero predominio de sueño N3 y sin eventos motores relevantes ni apneas. Se identificó un aumento patológico en el índice de despertares en ondas lentas durante N3 (4,4 y 4,7/h; normal <2,5), marcador electrofisiológico de parasomnia de sueño NREM. Adicionalmente, se objetivó un foco epileptiforme lateralizado en región temporal derecha, activado por el sueño y persistente durante el sueño profundo. No se registraron episodios comportamentales durante el estudio. **Discusión/Conclusiones**: La PSG permitió detectar simultáneamente marcadores específicos de epilepsia temporal y de parasomnia de sueño NREM, en ausencia de manifestaciones clínicas observables durante el registro. Este caso subraya la utilidad de la monitorización prolongada con EEG ampliado en la caracterización de trastornos del sueño de origen incierto, y respalda su papel en la toma de decisiones terapéuticas personalizadas en contextos de comorbilidad entre epilepsia y trastornos NREM.

**Agripnia Excitata: Más que un Estado Disociativo de Sueño. A Propósito de un Caso**

Alina Havrylenko Vynogradnyk^1^, María Carmen Lloria Gil^1^, Estefanía Rivas Navas^1^, Gricela María Sorto Bueso^1^, Diego Francisco Peña Olaya^1^, Fernando Vázquez Sánchez^1^, Joseba Soto Ibáñez^1^

^1^Hospital Universitario de Burgos, 09006 Burgos, Castilla y León, España

**Introducción y Objetivos**: Agripnia excitata o estatus disociatus: es la expresión extrema de disociación de los estados de sueño. Existen 3 tipos: desde la vigilia, desde el sueño REM y desde el sueño NREM. **Material y Métodos**: Presentamos el caso de un hombre de 64 años con movimientos atípicos durante la noche presenciados por la acompañante. Antecedentes médicos: espondiloartropatía seronegativa, migrañas y episodios confusionales con amnesia desde 2017. En estudio por Neurología. Parkinsonismo, deterioro cognitivo leve y trastorno ansioso-depresivo. En la anamnesis se recogen movimientos bruscos de vibración en extremidades durante el sueño tanto nocturno como diurno, que van aumentando en intensidad y se acompañan de somniloquios; el paciente no es consciente de los movimientos y aqueja clínica de cansancio durante el día, por lo que se realiza estudio de sueño nocturno. **Resultados**: En el estudio polisomnográfico se observa severa desorganización y fragmentación del sueño sin poder distinguir la estructura del mismo, asociándose con una conducta hipermotora prácticamente continua durante el registro. Sospecha diagnóstica: status dissociatus en estudio de la causa subyacente. Sin hallar enfermedad neurodegenrativa subyacente en el estudio diagnóstico subsiguiente. Se pauta tratamiento con Rivotril, observando mejoría significativa de la estructura de sueño en polisomnografía de control. **Discusión/Conclusiones**: El estatus disociatus, o agripnia excitata es un cuadro de ausencia o disminución severa de sueño a causa de trastornos orgánicos asociado a hiperactivación motora o autonómica. Se relaciona con enfermedades neurodegenerativas, autoinmunes, síndrome de abstinencia, entre otros de ahí la importancia de completar estudio, siendo los datos polisomnográficos claves para documentar el proceso de estructuración de sueño.

**Test de Latencias Múltiples: Descripción de una Cohorte en una Unidad de Sueño**

Romina Zapatel^1^, Beatriz Echeveste González^1^, Óscar Manzanilla Zapata^1^, Verónica Cortés Jiménez^1^, Carmen García Penco^1^, Manuel Alegre Esteban^1^, Elena Urrestarazu Bolumburu^1^

^1^Clínica Universidad de Navarra, 31008 Pamplona, España

**Introducción y Objetivos**: La hipersomnia diurna tiene múltiples causas que pueden ser primarias o secundarias. En el proceso diagnóstico el test de latencia múltiple durante el sueño (TLMS) suele emplearse como “gold estándar”. El objetivo de este estudio es describir una cohorte de pacientes a los que se les realizó un test de latencias múltiple. **Material y Métodos**: Estudio retrospectivo de una cohorte de pacientes a los que se les realizó un test de latencia múltiple con una PSG la noche previa, entre los años 2019–2024. Se analizó el motivo de la petición y el diagnóstico final obtenido, los síntomas de los pacientes, el número de siestas realizadas y, además, en función del diagnóstico final se valoró la estructura del sueño de la noche anterior. El análisis estadístico se realizó con SPSS con valor de *p* <0,50. **Resultados**: Se analizaron 101 pacientes, 47,5% eran mujeres. La edad media fue de 38 (6–84) años. El motivo de solicitud en el 74,2% fue por somnolencia diurna. 48 pacientes realizaron 4 siestas. 29 paciente tuvieron como diagnóstico final privación de sueño, 20 hipersomnia idiopática, 17 AOS leve, 17 narcolepsia, 9 hipersomnia residual. Los pacientes con diagnóstico final de narcolepsia entraban en sueño en más siestas y en menor tiempo que el resto de los grupos analizados (*p* < 0,010). Al analizar la estructura del sueño se evidencia mayor porcentaje de fase N3 en los pacientes con diagnostico final de narcolepsia frente a la de los pacientes con hipersomnia idiopática (*p* < 0,40). El resto de fases no muestra alteraciones significativas entre las diferentes patologías. **Discusión/Conclusiones**: La hipersomnia diurna es una queja frecuente, afectando tanto a niños como a adultos mayores y las causas de la misma pueden ser muy variadas, teniendo que tener en mente un diagnóstico diferencial amplio. En nuestra cohorte la privación de sueño fue la causa más frecuente. La estructura del sueño de pacientes con narcolepsia difiere respecto a la de los pacientes con hipersomnia idiopática.

**Guardias Médicas y Cronodisrupción: Un Estudio Sobre la Calidad del Sueño en Médicos Residentes de Canarias**

Víctor Melián Díaz^1^, Octavio Jiménez Vega^2^, Eva Amador Gil^1^, Alexander Baloira Armas^1^, Leticia del Carmen Santana Cáceres^3^, Irene Carratalá Nebot^1^, Beatriz Navarro Rivero^1^

^1^Complejo Hospitalario Univerisario Insular Materno Infantl de Las Palmas, 35016 Las Palmas de Gran Canaria, España

^2^Hospital Universitario de Gran Canaria Doctor Negrín, 35010 Las Palmas de Gran Canaria, España

^3^Adjunta Neurofisiologia, 35010 Las Palmas de Gran Canaria, España

**Introducción y Objetivos**: El ejercicio médico de los Médicos Internos Residentes (MIR) comporta una elevada exigencia física y cognitiva, a menudo en detrimento del descanso nocturno. La disrupción del sueño asociada al régimen de guardias de 24 horas constituye un factor de riesgo para la salud del profesional y compromete la seguridad asistencial. Este estudio evalúa la calidad del sueño en una cohorte de Médicos Internos Residentes (MIR) de la Comunidad Autónoma de Canarias mediante el Pittsburgh Sleep Quality Index (PSQI). **Material y Métodos**: Estudio observacional, transversal y multicéntrico realizado en 2024 con 233 MIR del Servicio Canario de Salud. Se recogieron datos sobre especialidad, año de residencia, número de guardias mensuales y percepción del sueño. Se utilizó el PSQI para evaluar calidad de sueño y sus dimensiones. **Resultados**: El PSQI medio global fue de 8,17 puntos, superando ampliamente el umbral patológico (puntuación <5). El 81,5% de los participantes presentó una calidad del sueño alterada. Los componentes más afectados fueron la calidad subjetiva del sueño (media: 1,52) y la disfunción diurna (media: 1,58). El uso de hipnóticos fue escaso. No se evidenciaron diferencias significativas entre la puntuación del PSQI y el número de guardias ni el año de residencia (*p* < 0,05), aunque los residentes de primer y segundo año manifestaron una mayor percepción de afectación. El 85,4% refirió que las guardias alteraban su descanso, y el 39% reportó alteraciones en la arquitectura del sueño. **Discusión/Conclusiones**: La alteración del sueño en los MIR es altamente prevalente y relevante, con especial impacto en los primeros años de residencia y en especialidades con alta carga asistencial. Aunque no se evidenciaron asociaciones estadísticamente significativas con el número de guardias, la percepción subjetiva y los ítems más afectados del PSQI reflejan una disrupción sustancial del descanso. Estos hallazgos respaldan la necesidad de estrategias organizativas que mitiguen el impacto de las guardias sobre la salud del médico en formación.

**SleepIA: Redefiniendo el Cribado del SAOS con el uso de la IA**

Inmaculada Rodríguez Ulecia^1^, Antonio Gabriel Ravelo García^2^, Octavio Jiménez Vega^1^, Amanda Labrador Rodríguez^1^, Víctor Melián Díaz^3^, Génesis Arteaga Requena^1^, Ayoze N. González Hernández^1^

^1^Hospital U. de Gran Canaria Doctor Negrín, 35010 Las Palmas de Gran Canaria, Las Palmas, España

^2^Universidad de Las Palmas de Gran Canaria, 35001 Las Palmas de Gran Canaria, Las Palmas, España

^3^Complejo Hospitalario Universitario Insular - Materno Infantil de Canarias, 35016 Las Palmas de Gran Canaria, Las Palmas, España

**Introducción y Objetivos**: La polisomnografía (PSG) es el estándar diagnóstico para la apnea obstructiva del sueño (SAOS), al permitir una evaluación integral de variables respiratorias, neurológicas y cardiovasculares. Sin embargo, técnicas ambulatorias como la pulsioximetría o la poligrafía respiratoria no incorporan parámetros como la actividad cortical, lo que puede llevar a una subestimación del índice apnea-hipopnea (IAH), especialmente en casos leves o sin desaturaciones evidentes. **Material y Métodos**: Desarrollar y validar un nuevo índice denominado Neuro-Integrated Sleep Event Index (NISEI), basado en inteligencia artificial (IA), entrenado directamente con PSG y capaz de detectar automáticamente eventos respiratorios clínicamente relevantes a partir de señales mínimas (pulsioximetría y ECG unicanal). El índice incorpora variables como desaturaciones, pulse transit time (PTT), variabilidad de la frecuencia cardíaca (HRV) y patrones de acoplamiento cardiopulmonar (CPC). Estudio observacional en curso con 40 pacientes con sospecha de SAOS. A cada paciente se le realizó una PSG completa y un registro simplificado en paralelo (oximetría, ECG y EEG). Mediante análisis espectral del CPC se identificaron patrones de sueño estable e inestable, correlacionados con eventos respiratorios anotados manualmente. La IA fue entrenada y validada en colaboración con ingenieros. **Resultados**: El índice NISEI mostró alta correlación con el IAH (r = 0,87; *p* < 0,001), con sensibilidad del 91% y especificidad del 90% para SAOS moderado-severo. Superó a la pulsioximetría convencional en la detección de SAOS leve, al integrar marcadores autonómicos y de fragmentación cortical. **Discusión/Conclusiones**: SleepIA, mediante el índice NISEI y señales mínimas, mejora la detección del SAOS, incluso sin desaturación. Ofrece un cribado inteligente y accesible, útil donde el screening rutinario puede infraestimar la patología. Acerca la medicina del sueño al entorno domiciliario e impulsa el uso de la inteligencia artificial en la práctica clínica diaria en medicina del sueño.

**Apnea Central del Sueño Como Manifestación de Malformación de Chiari Tipo I**

Jesús David Jaramillo Álvarez^1^, Shijia Li Chen^1^, Ramón Josep Gorgues Torres^1^, Fernanda Romero Puerta^1^

^1^Hospital Universitario Miguel Servet Zaragoza, 50009 Zaragoza, Aragón, España

**Introducción y Objetivos**: Se pretende describir el caso de un adolescente con apnea central del sueño secundaria a malformación de Chiari tipo I (MCI), con evolución clínica, diagnóstica y terapéutica, destacando la importancia del diagnóstico etiológico en jóvenes con apnea central. La apnea central del sueño (ACS) se caracteriza por la ausencia de esfuerzo respiratorio durante episodios de cese del flujo aéreo. Su prevalencia es baja en la población general, especialmente en jóvenes. Dentro de sus causas secundarias la MCI es una entidad rara pero relevante. **Material y Métodos**: Se presenta el caso de un varón de 16 años, con historia de síntomas respiratorios altos. Consultó por roncopatía intensa y apnea. Se realizaron estudios que incluyeron poligrafía y polisomnografía, así como pruebas de imagen. La evolución terapéutica incluyó distintos modos de presión positiva continua en la vía aérea (CPAP, BiPAP) y, finalmente, tratamiento quirúrgico. **Resultados**: Inicialmente se realiza una poligrafía respiratoria no vigilada que muestra IAH 86/h con repercusión oximétrica severa por lo que se inicia tratamiento con CPAP y posteriormente con BiPAP, sin remisión de los episodios por lo que acuden de manera particular a neurología y se realiza RMN con diagnóstico de MCI. La polisomnografía de noche partida evidenció un IAH de 10,4/h con eventos respiratorios de tipo central sin gran repercusión oximétrica. El paciente fue intervenido quirúrgicamente con gran mejoría clínica por lo que se suspendió el tratamiento y el paciente permanece asintomático. **Discusión/Conclusiones**: La MCI debe considerarse en el diagnóstico diferencial de ACS en niños y adolescentes. El tratamiento quirúrgico puede mejorar significativamente la clínica, aunque algunos casos pueden requerir soporte ventilatorio complementario. La integración multidisciplinar entre neumología, neurología, neurocirugía y neurofisiología es clave en estos casos.

**El Sueño de los Adolescentes a Estudio: ¿Duermen lo Suficiente Para Rendir en Clase?**

Carla Pía Martínez^1^, Larraitz Ortega Álvarez^2^, María Comas^3^, Almudena Fernández Vicente^3^, María Mercedes Hernández Hernández^3^, Jorge Ullate Mora^3^, Ainhoa Álvarez Ruiz de Larrinaga^1^

^1^Hospital Universitario Araba, 01009 Vitoria‑Gasteiz, Álava, País Vasco, España

^2^Universidad de Mondragón, 20500 Mondragón (Arrasate), Gipuzkoa, País Vasco, España

^3^Instituto de Investigación Sanitaria Bioaraba, 01009 Vitoria‑Gasteiz, Álava, País Vasco, España

**Introducción y Objetivos**: El sueño es básico para el desarrollo y aprendizaje. En la adolescencia, la tendencia a la vespertinidad y el horario lectivo en secundaria (ESO), nos hace plantearnos: ¿duermen los escolares lo suficiente para rendir en clase? Objetivo: Analizar el Tiempo total de sueño (TTS) en una muestra de estudiantes de ESO, comparando si existen diferencias durante la semana (TTS-LV) y los fines de semana (TTS-SD) así como la hora de acostarse entre semana (HS-LV) y los fines de semana (HS-SD) y comprobar si existe una correlación entre el TTS y la nota media (NM). **Material y Métodos**: Se analizaron en una muestra de estudiantes de 2º y 3º de ESO de Vitoria-Gasteiz, datos de actímetros registrados durante 2 semanas, recogiendo TTS, TTS-LV, TTS-SD, HS-LV y HS-SD. El análisis estadístico se realizó mediante prueba de rangos de Wilcoxon. La NM de los participantes se correlacionó con el TTS mediante coeficiente de Pearson para muestras paramétricas y Spearman para no paramétricas. **Resultados**: Participaron 70 estudiantes, siendo el 56,5% mujeres, con edad media de 13,6 ± 0,67 años. Se identificó un TTS inferior a 8 horas en el 85% en TTS-LV y el 62% en TTS-SD. La diferencia es significativa (*p* = 0,0044) y no hubo diferencias con respecto al sexo. Además, la HS-LV media fue de 22:25 horas (±49 min) y la HS-SD fue a las 23:25 (±112,2 min), siendo este hallazgo también significativo (*p* < 0,001). Obtuvimos una NM de 7,1 ± 1,27 (hombres 6,45 ± 1,07 y mujeres de 7,54 ± 1,23), existiendo una correlación significativa entre la NM y el TTS-LV con un índice de correlación de 0,253 (*p* = 0,034); siendo más potente en las mujeres 0,345 (*p* = 0,032). **Discusión/Conclusiones**: La mayoría de participantes duermen menos de 8 horas, siendo mayor este porcentaje durante la semana. Los voluntarios se acostaron hasta 1 hora más tarde los fines de semana. Por ello, el horario escolar no parece adecuado para esta edad. El sueño influye en el rendimiento escolar, sobre todo en las mujeres participantes. Futuros proyectos estarán enfocados a valorar si mayor TTS mejora las notas.

**Uso de Pitolisant en Pacientes con Narcolepsia: Experiencia Clínica en Vida Real en una Cohorte de 36 Pacientes**

Ana Eugenia Maza Jiménez^1^, María Aguilar Andújar^1^, Rocío López Álvarez-Ossorio^1^, Paula Romero González^1^, María del Carmen Menéndez de León^1^

^1^Hospital Universitario Virgen Macarena, 41071 Sevilla, España

**Introducción y Objetivos**: La narcolepsia es un trastorno crónico del sueño que cursa con somnolencia diurna excesiva y otros síntomas como cataplejía o alucinaciones hipnagógicas. Se clasifica en tipo 1 (con cataplejía y déficit de hipocretina) y tipo 2 (sin estos hallazgos). Pitolisant, un agonista inverso del receptor H3 de histamina, es una opción terapéutica eficaz y bien tolerada. Este estudio evalúa su uso en pacientes con narcolepsia en una unidad de sueño, valorando eficacia percibida, tolerabilidad y factores asociados. **Material y Métodos**: Se realizó un análisis retrospectivo de 36 pacientes narcolépticos tratados con pitolisant entre 2022 y 2025. Se recopilaron datos clínicos y terapéuticos, como tipo de narcolepsia, dosis máxima alcanzada, uso en monoterapia o combinado, efectos adversos y respuesta clínica. Se compararon subgrupos según si pitolisant fue tratamiento de primera línea o tras otros fármacos. **Resultados**: Pitolisant fue introducido como primera línea en el 44,4%, logrando una tasa de respuesta del 100%: el 62,5% mejoró parcialmente y el 37,5% alcanzó una mejoría completa. En segunda línea, la tasa de respuesta global fue del 75%, con un 25% sin mejoría, concentrados en pacientes bajo politerapia con oxibato sódico. El régimen combinado predominó (52,8%). La dosis más común fue 36 mg/día. Se notificaron efectos adversos en el 16,7%, destacando ansiedad y nerviosismo; un 13,9% precisó suspensión del tratamiento. Dos casos con eventos adversos relevantes presentaban comorbilidad psiquiátrica y uso simultáneo de psicofármacos. No hubo reacciones graves. **Discusión/Conclusiones**: Pitolisant mostró buena eficacia y tolerancia, sobre todo como tratamiento inicial. La respuesta más deficiente en pacientes con politerapia sugiere que la complejidad terapéutica o interacciones pueden afectar la eficacia percibida. Los efectos adversos fueron leves, con mayor riesgo en pacientes con antecedentes psiquiátricos. Estos resultados apoyan el uso precoz de pitolisant y destacan la necesidad de estudios prospectivos más amplios.

**Apnea Central de Sueño en Población Pediátrica de Edad Preescolar y Escolar: Una Serie de Casos**

Alberto Ulloa Meijide^1^, Valeria Fra Mosquera^1^, Beatriz Soria Soriano^1^, Sonia Fernández Gil^1^, Iria Lagoa Labrador^1^, Elena Lojo Lendoiro^1^

^1^Hospital Universitario Álvaro Cunqueiro, 36312 Vigo, España

**Introducción y Objetivos**: La apnea central del sueño (ACS) es un trastorno respiratorio caracterizado por la interrupción de la respiración durante el sueño, debida a diversas causas (fundamentalmente cardíacas, neurológicas, por fármacos/tóxicos o idiopática). Aunque más común en prematuros, lactantes y adultos, también puede presentarse en población pediátrica escolar. Nuestro objetivo es describir las características clínicas y polisomnograficos de una serie de pacientes pediátricos con ACS en estudios de PSG en nuestro centro. **Material y Métodos**: Se realizó un análisis retrospectivo de los estudios de PSG realizados en nuestro centro en pacientes pediátricos (2–13 años) entre 2022 y 2025, identificándose 21 pacientes con hallazgos compatibles con ACS según los criterios de ICSD-3 (clínica compatible, >5 apneas o hipopneas centrales/hora, representando éstas >50% de los eventos respiratorios totales). Se recopilaron datos sobre comorbilidades, estudios complementarios y evolución. **Resultados**: En la mayoría de nuestros pacientes (15 de 21) la ACS no se asociaba con comorbilidades evidentes. Dos de nuestros pacientes estaban diagnosticados de distrofia muscular de Duchenne; otro de neuropatía axonal gigante; cuatro presentaron un retraso global del desarrollo con discapacidad intelectual, sólo uno de ellos con diagnóstico genético y dos de ellos asociando malformaciones cardíacas y a otros niveles. Los episodios de apnea fueron más frecuentes durante el sueño REM, siendo los síntomas más comunes como motivo de derivación apneas presenciadas y ronquido. **Discusión/Conclusiones**: La apnea central del sueño en niños en edad preescolar y escolar es un trastorno poco frecuente pero relevante, a menudo asociado con comorbilidades cardiovasculares o genéticas. Un diagnóstico temprano, basado en la PSG y la evaluación clínica, es fundamental para su manejo adecuado.

